# Network pharmacology and experimental validation to investigate the mechanism of action of Zhilong Huoxue Tongyu capsule in the prevention and treatment of diabetic cardiomyopathy

**DOI:** 10.1371/journal.pone.0323745

**Published:** 2025-05-15

**Authors:** Fang Yang, Gang Luo, Meng-nan Liu, Ping Liu, Dan Wu, Hao-ling Chen, Shan Li, Si-Jin Yang, Li Dong

**Affiliations:** 1 National Traditional Chinese Medicine Clinical Research Base and Cardiology department of the Affiliated Traditional Chinese Medicine Hospital of Southwest Medical University, Luzhou, China; 2 Institute of Integrated Chinese and Western Medicine, Southwest Medical University, Luzhou, China; Georgia State University, UNITED STATES OF AMERICA

## Abstract

**Background:**

Diabetes cardiomyopathy (DCM) is a prevalent complication of diabetes, characterized by a multifaceted pathogenesis. Zhilong Huoxue Tongyu Capsule (ZL), a traditional Chinese medicine, is extensively employed for the treatment of cardiovascular diseases. Thus, this study aimed to comprehensively explore the mechanism of action of ZL on DCM.

**Method:**

Network pharmacology approaches were applied to predict the potential pathways and targets of ZL on DCM. Then, a DCM model mouse was constructed and divided into a control group, DCM group, DCM + ZL group, SB203580 group, and DCM + R group. The DCM + ZL group was administered 6.24g/kg/d ZL via gavage, the SB203580 group was given 1 mg/kg/d SB203580 (p38MAPK inhibitor) via intraperitoneal injection, the DCM + R group received 4 mg/kg/d rosiglitazone via gavage, and the control group and DCM group were given equal volume of physiological saline by gavage. The intervention period lasted for 6 weeks to verify these key targets.

**Result:**

Network pharmacology analyses identified 45 active ingredients in ZL linked to 719 potential targets, forming an herbal compound-target network. Screening of databases revealed 1032 DCM-related targets, with MAPK14, TNF, FOS, AKT1, and IL-10 emerging as key hub genes from PPI network analysis. Additionally, enrichment analysis indicated that the candidate targets were enriched in response to the MAPK signaling pathway. Finally, in vivo studies in DCM mice demonstrated that ZL significantly mitigated myocardial fibrosis and down-regulated the expression of p-P38MAPK, TNF-α, α-SMA, and Collagen-I proteins in myocardial tissue.

**Conclusion:**

Our results collectively indicated that ZL can effectively ameliorate diabetes cardiomyopathy, possibly by modulating the P38MAPK signaling pathway.

## Introduction

As is well documented, diabetes mellitus is a prevalent metabolic disease worldwide and has emerged as a significant global public health challenge. According to the latest projections by the International Diabetes Federation (IDF), the current prevalence of diabetes among adults is approximately 10.2%. It is estimated that the global diabetic population will reach 578 million by 2035 and 700 million by 2045 [1]. Diabetic cardiomyopathy is defined as a type of cardiomyopathy distinguished by structural and systolic-diastolic dysfunction of the heart in individuals with diabetes, excluding comorbidities such as hypertension, coronary artery disease, and valvular heart disease [2]. Epidemiological studies have established a close association between diabetes and heart failure. Indeed, findings from the Framingham study indicate that compared to non-diabetic individuals, diabetic men have a 2.4-fold higher risk of heart failure, while women have a staggering 5-fold higher risk [3]. Existing evidence suggests that the progression of DCM is associated with myocardial cell hypertrophy and fibrosis, as well as the activation of neurohumoral mechanisms such as hyperglycemia, metabolic disorders, insulin resistance, the renin-angiotensin-aldosterone system (RAAS), and the sympathetic nervous system (SNS). Moreover, the accumulation of free fatty acids (FFAs), impaired calcium homeostasis, and the depletion of glucose transporter proteins further exacerbate myocardial cell damage [4–8].

Despite significant advances in modern medicine for the treatment of diabetes and its complications, the complex pathogenesis of DCM, involving multiple intertwined systems and factors, renders its therapeutic strategies challenging and limited [9]. At present, the treatment of DCM primarily relies on angiotensin-converting enzyme inhibitors, angiotensin receptor blockers, aldosterone receptor antagonists, calcium channel blockers, beta-blockers, statins, cardiac glycosides, and sodium-dependent glucose transporters 2 (SGLT-2) inhibitors [10,11]. Notably, these drugs partially inhibit ventricular remodeling and alleviate symptoms of heart failure. However, these medications are typically associated with adverse effects, predominantly act on single targets, and may lead to interactions and contraindications when used in combination. Besides, patient compliance is generally poor, limiting their therapeutic efficacy. Therefore, there is a pressing need to identify new therapeutic mechanisms for myocardial fibrosis, conducting in-depth clinical research, and developing specific anti-fibrotic drugs. In this regard, traditional Chinese medicine (TCM) demonstrates unique advantages. Specifically, TCM formulations typically have minimal side effects, diverse components, multi-target effects, and multi-pathway regulatory mechanisms and have been universally used in early disease treatment [12,13]. In recent years, research on the use of traditional Chinese medicine in the prevention and treatment of DCM has garnered considerable attention, emerging as a focal point in international academic investigations.

ZL is primarily composed of the roots of huangqi (English name: Astragalus), the dried whole animal of dilong (English name: Earthworm), the stem/twig of daxueteng (English name: Sargentgloryvine), the stem/twig of guizhi (English name: Cassia), and the dried whole animal of shuizhi (English name: Leech) in a ratio of 8:4:4:3:1 [14,15]. Earlier studies indicated that it exhibits anti-inflammatory, anti-apoptotic, anti-necrotic, and anti-atherosclerotic effects and has been widely utilized in the treatment of cardiovascular and cerebrovascular diseases [16–18]. Nonetheless, its preventive effect and mechanism of action on DCM remain elusive, warranting further investigation.

This study utilized network pharmacology methods, integrating multiple database resources, to thoroughly investigate the therapeutic targets and potential mechanisms of ZL in the treatment of dilated cardiomyopathy (DCM). Key therapeutic targets were identified by identifying the active components of ZL and DCM-related targets. Additionally, GO and KEGG enrichment analyses were performed to reveal their biological functions and interaction networks. Ultimately, the role of ZL was validated in a mouse model of DCM to support targeted therapeutic strategies for the management of DCM.

## Materials and methods

### Collection of active compounds of ZL and their potential targets

Using the Traditional Chinese Medicine Systems Pharmacology Database and Analysis Platform (TCMSP, https://tcmspw.com/tcmsp.php), we retrieved the active ingredients of “*huangqi*”, “*guizhi*”, and “*daxueteng*” in ZL capsule. By setting the screening criteria as an oral bioavailability (OB) ≥ 30% and a drug-likeness (DL) index ≥ 0.18, we obtained the corresponding targets [19,20]. Since TCMSP does not include animal-derived medicinal ingredients, we searched for the components of the animal medicines “*shuizhi*” and “*dilong*” in the Bioinformatics analysis tools for the molecular mechanisms of traditional Chinese medicine (Batman-TCM, http://bionet.ncpsb.org.cn/batman-tcm/), using a significance level of P < 0.05 as the screening criterion. After obtaining the corresponding targets, the names of these targets were then standardized using the UniProt database (https://www.uniprot.org) [21].

### Screening of potential targets for DCM

The search term “diabetic cardiomyopathy” was used to identify targets in the GeneCards database (https://www.genecards.org/) with a relevance score of ≥ 10 [22]. Moreover, the OMIM database (https://www.omim.org/) [23], Pharmgkb (https://www.pharmgkb.org/) [24], and the DrugBank database (https://go.drugbank.com/) was utilized to retrieve relevant data [25]. Duplicate targets were eliminated to acquire diabetic cardiomyopathy-related targets.

### Constructing the database of intersecting drug-disease target genes

To identify the intersected DCM gene targets intervened by ZL active ingredients, the “Venn” editing package within R4.2.2 software was utilized to establish the intersection between the drug targets of ZL and the disease targets of DCM.

### Constructing the drug-target network interactions

To scientifically rationalize the relationship between compounds and predicted overlapping targets, we utilized Cytoscape v3.9.1 (https://cytoscape.org/) to construct a comprehensive drug-target network interactions model. Within this network, compounds exhibiting high degree values are identified as pivotal bioactive compounds critical for the therapeutic management of DCM.

### Construction and analysis of the protein-protein (PPI) interaction network

Overlapping targets of DCM and ZL bioactive compounds were imported into the STRING database (https://string-db.org/) [26], with the species set to “*Homo sapiens*” and interactions ≥ 0.9 as the minimum threshold. Following this, the acquired PPI data were imported into Cytoscape v3.9.1 in “tsv” format for network construction. Using R 4.2.2 software to obtain core targets. Core targets are filtered based on the following topological parameters, including betweenness centrality (BC), closeness centrality (CC), degree centrality (DC), eigenvector centrality (EC), network centrality (NC), and local average connectivity (LAC). The values of these parameters indicate the importance and influence of the relevant nodes in the network. The cutoff value is set as the median. The network topology of the PPI network was then assessed using the CytoNCA plugin to further identify and filter the core and key targets within the network [27]. Six key indices were computed, namely, betweenness centrality (BC), closeness centrality (CC), degree centrality (DC), eigenvector centrality (EC), network centrality (NC), and local average connectivity (LAC). Higher quantitative values of the parameters indicate higher importance of the nodes in the network. The target node with all 6 parameters above the corresponding median in the PPI network is selected to construct the new PPI network.

### GO and KEGG pathway enrichment analysis

GO and KEGG enrichment analyses were performed using R4.2.2 software and the “cluster Profiler” and “enrichplot” packages. The data obtained from KEGG pathway enrichment analysis were uploaded to an online data analysis and visualization platform (https://www.cnsknowall.com/) for further visualization. q ≤ 0.05 was considered statistically significant.

### Constructing drug-target-pathway networks

To establish the drug-target-pathway network, we employed Cytoscape v3.9.1 to construct the interaction relationships among ZL bioactive compounds, intersecting targets, and the top 20 KEGG pathways. In this constructed network, nodes represent bioactive compounds, targets, and pathways, whereas edges depict the interactions among these three entities.

### Molecular docking

To establish a molecular docking framework, we downloaded the two-dimensional (2D) structures of core drug ligands from the PubChem database (https://pubchem.ncbi.nlm.nih.gov/) and converted them into three-dimensional (3D) structures using Chem3D software, subsequently transforming them into “PDB” format as ligands with PyMOL [28]. We then searched the Protein Data Bank (https://www.rcsb.org/) for core proteins associated with DCM-related genes and prepared them for docking using AutoDockTools by adding hydrogen atoms, calculating charges, and eliminating free water molecules, designating them as macromolecules. Docking grid boxes were constructed for each target protein’s binding sites and saved in “pdbqt” format. AutoDock Vina software was utilized to perform the molecular docking [29]. PyMOL was employed for visualization [30]. Detailed information on protein targets, their PDB IDs, and central coordinates (x, y, z-centre) is provided in Table 1.

**Table 1:** Information on protein targets and their center coordinates.

Target protein	PDB ID	Centre coordinates (x, y, z centre)	Grid box size
MAPK14 TNF AKT1	2FST 1NCF 3O96	11.675, 5.162, 17.925 21.259, 14.649, 34.769 6.29, -7.942, 17.261	X = 40, Y = 40, Z = 40 X = 40, Y = 40, Z = 40 X = 40, Y = 40, Z = 40

## Experimental validation

### Drug preparation

ZL was processed in the preparation room of the Traditional Chinese Medicine Hospital of Southwest Medical University, with the Sichuan medical institution preparation number 20180216. Rosiglitazone Tablets, specifications of 4 mg * 7 tablets * 2, bearing the national drug approval number H20030569, provided by Chengdu Hengrui Pharmaceutical Co.,Ltd.

### Animals

Male SPF C57BL/6J mice, aged 6 weeks and weighing 19-21g, were procured from Chongqing Tengxin Biotechnology Co., Ltd. (Animal Generation Certificate of Conformity No. SCXK (Beijing) 2019–0010). All mice were housed under standard conditions, with a 12 h light/dark cycle, a temperature of 20–26°C, a humidity level of 40–60%, and provided with ad libitum access to food and water. This study was approved by the Animal Experiment Ethics Committee of Southwest Medical University (Approval No: SWMU20230081).

### DCM animal model

All the mice were adaptively fed for 1 week and then randomly divided into a control group and an experimental group. Those in the experimental group were intraperitoneally injected with streptozotocin (STZ, Solarbio, S8050-100mg) at a dose of 55 mg/(kg·d) for 5 consecutive days [31]. STZ was dissolved in a 0.1 mol/L citric acid-sodium citrate buffer solution with a pH of 4.0-4.5 to prepare a 1% solution, which was freshly prepared and used. The control group was simultaneously injected with an equivalent volume of citric acid-sodium citrate buffer solution. After one week, the fasting blood glucose levels of mice were measured through tail vein blood sampling. A fasting blood glucose level of mice reaching or exceeding 16.7 mmol/L, accompanied by symptoms such as polydipsia and polyuria, confirmed the successful establishment of the Type 2 Diabetes Mellitus (T2DM) model. Following the successful construction of the T2DM model, mice in the experimental group were maintained on a high-fat diet (Base 66.6%, sucrose 20%, lard 10%, cholesterol 2.5%, sodium cholate 1%, fat content ratio 35%, and total heat 3.95 kcal/g) for 12 weeks to induce the Diabetic Cardiomyopathy (DCM) model. H&E staining displayed that myocardial fibers were intact and evenly distributed in the Con group, whereas cardiomyocytes were hypertrophied and myocardial fibers were unevenly distributed in the DCM group, indicating the successful construction of the DCM model.

### Animal grouping and drug administration

The experimental mice were randomly assigned to four groups: DCM, DCM + ZL, SB203580 (P38MAPK inhibitor), and DCM+rosiglitazone (DCM + R), with each group comprising eight mice. Specifically, the DCM + ZL group received ZL (6.24 g/kg/d) via gavage [32]; SB203580 was intraperitoneally administered at a dose of 1 mg/kg/d in the SB203580 group [33], while a suspension of rosiglitazone at a dose of 4 mg/kg/d was given by gastric gavage in the DCM + R group [34]. The control (n = 8) and DCM groups were administered an equivalent volume of 0.9% normal saline by gavage. This procedure was performed once daily for six consecutive weeks. Following the final administration, all mice were anesthetized via intraperitoneal injection of 1% pentobarbital sodium and subsequently euthanized. The heart tissues were excised and either fixed in 4% paraformaldehyde or stored at -80°C for future experiments.

### Hematoxylin and Eosin (H&E) and Masson staining

The left ventricular myocardial tissue was fixed with 4% paraformaldehyde for 24 h, routinely dehydrated, paraffin-embedded, and then sectioned to a thickness of 3 μm. Next, these sections were subjected to H&E and Masson staining and then observed under a light microscope.

### Real-time fluorescence quantitative polymerase chain reaction (RTq-PCR) detection

The left ventricular myocardial tissue was harvested from mice, and total RNA was extracted using TRIzol reagent (Invitrogen, 15596026). RNA concentration and purity were assessed, and subsequently, the total RNA was reverse-transcribed into cDNA using an RT kit (Toyobo, FSQ-201). This cDNA served as a template for amplification under the following reaction conditions: pre-denaturation at 95°C for 60 seconds, followed by a cycling reaction consisting of 95°C for 15 seconds, 60°C for 15 seconds, and 72°C for 45 seconds, repeated for a total of 40 cycles. Subsequently, the Ct value was determined through general dissolution curve (95°C for 15 seconds, 60°C for 60 seconds, and 95°C for 15 seconds) using a PCR instrument. Relative expression levels were determined utilizing the 2^-△△Ct^ method, with GAPDH serving as an internal reference control. The primer sequences for GAPDH, TNF-α, α-SMA, and Collagen-I are listed in Table 2.

**Table 2:** Primer sequences for real-time RTq-PCR.

Symbol	Primer sequence (Forward, Reverse)
GAPDH	Forward: GGACCTCATGGCCTACATGG
TNF-α Collagen-I α-SMA	Reverse: TAGGGCCTCTCTTGCTCAGT Forward: GGTGCCTATGTCTCAGCCTCTT Reverse: GCCATAGAACTGATGAGAGGGAG Forward: ACAGTCGCTTCACCTACAGC Reverse: TTCGATGACTGTCTTGCCCC Forward: GTCCCAGACATCAGGGAGTAA Reverse: TCGGATACTTCAGCGTCAGGA

### Western blot analysis

20mg of the left ventricular myocardial tissue was weighed, washed with pre-cooled PBS 3 times to remove blood stains, and placed in a 2 mL EP tube. Next, 200 µ L RIPA lysis solution (Beyotime, P0013B) and 2 µ L protease inhibitor PMSF (100mM) (Servicebio, G2008-1ML) and 2 µ L phosphorylated protease inhibitors (Servicebio, G2007-1ML) was introduced into the tube, and a high-speed, low-temperature tissue grinder was employed for homogenization. The tube was then placed in an ice bath for 30 min, followed by centrifugation at 4°C and 12000 r/min for 10 min. Afterward, the supernatant was collected, and the BCA protein concentration measurement kit was used to determine protein concentration. Prior to electrophoresis, the protein samples were denatured by mixing with 2x Laemmli loading buffer (absin, abs9237), followed by boiling at 100°C for 10 min and cooling to room temperature. 20 μg of protein was loaded for electrophoresis (SDS-PAGE, 5% and 10% separator gel), and the protein was transferred onto the membrane, which was blocked with 5% BSA sealing solution (Solarbio, SW3015–100ml) at room temperature for 1h. Thereafter, the membrane was incubated with primary antibodies against p-P38MAK (1:1,000, ab195049, Abcam), P38MAK (1:1,000, ab170099, Abcam), TNF-α (1:2,000, AF7014, Affinity), and GAPDH (1:2,000, GB12002, Servicebio) overnight at 4°C. Then, the membrane was rinsed with 1x TBST buffer (Solarbio, T1085-500ml) 5 times, the membrane was rinsed with TBST buffer 5 times, followed by incubation with goat anti-rabbit IgG (1:40,000, S0001, Affinity) conjugated with horseradish peroxidase (HRP) at room temperature for 2 hours. Finally, the membrane was washed 5 times, and protein bands were visualized using a chemiluminescent ECL kit (Affinity, kf8005). Bands were densitometrically analyzed using Image J software (National Institutes of Health, USA).

### Immunofluorescence analysis

Paraffin sections were routinely deparaffinized and rehydrated in water, followed by antigen retrieval and delineation with a histochemical pen. Subsequently, a drop of tissue autofluorescence quencher was added, and the sections were incubated at room temperature for 30 minutes. Following blocking with goat serum, the sections were incubated with primary antibodies against p-P38MAK (1:200, AF4001, Affinity), TNF-α (1:200, ab220210, Abcam), α-SMA (1:200, AF1032, Affinity), and Collagen I (1:200, AF7001, Affinity) overnight at 4°C. They were then washed with PBS and incubated with species-specific secondary antibodies (labeled with CY3) corresponding to the previously used primary antibodies for 50 minutes at room temperature. After PBS washing, DAPI stain was applied, and the sections were incubated for 10 minutes in the dark at room temperature. Thereafter, tissue autofluorescence quencher B was added, and the sections were incubated for an additional 5 minutes in the dark at room temperature. Finally, the sections were rinsed with water, mounted with an antifluorescence quenching sealant, and observed under a fluorescence microscope (Leica DM500, Japan).

### Statistical analysis

All data were presented as mean ± standard (SD) and analyzed and visualized using GraphPad Prism 9.4.1. Statistical comparisons were conducted using One-way ANOVA followed by Tukey’s test. *p* ＜ 0.05 was considered statistically significant.

## Results

### Active ingredients and predicted targets of ZL

**Fig 1:**
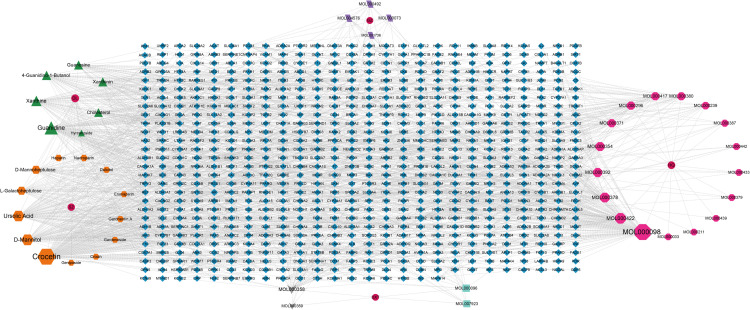
The active ingredients-targets diagram of ZL capsule The red circle represents the drug name, the magenta hexagon denotes the target of *huangqi* (HQ), the purple quadrilateral represents the target of *guizhi* (GZ), the green triangle denotes the target of *dilong* (DL), the orange hexagon indicates the target of *shuizhi* (SZ), the light green square represents the target of *daxueteng* (DXT), and the gray inverted triangle represents the common target of all drugs. The central blue rhombus represents the gene target.

A total of 45 active ingredients of Zhilong Huoxue Tongyu Capsule were identified through screening in the TCMSP and BATMAN-TCM databases. Among them, there are 17 components from *huangqi*, 6 components from *guizhi*, 13 components from *shuizhi*, 7 components from *dilong*, and 4 components from *daxueteng*. *daxueteng* and *guizhi* share two common components. After standardizing the target names through the UniProt database, we obtained predicted targets, among which there were 197 targets for *huangqi*, 52 targets for *guizhi*, 48 targets for *Daxueteng* 286 targets for *dilong*, and 342 targets for *shuizhi*. After removing duplicates, a total of 719 predicted targets were obtained. (Fig 1). The active ingredients corresponding to the targets of each drug are detailed in Table 3.

**Table 3:** The active ingredients for drug’s corresponding targets.

Herb	MOL ID	Molecule name	OB (%)	DL
*huangqi*	MOL000211 MOL000239 MOL000296 MOL000033 MOL000354 MOL000371 MOL000378 MOL000379 MOL000380 MOL000387 MOL000392 MOL000417 MOL000422 MOL000433 MOL000439 MOL000442 MOL000098	Mairin Jaranol hederagenin (3S,8S,9S,10R,13R,14S,17R)-10,13-dimethyl-17- [(2R,5S)-5-propan-2-yloctan-2-yl]-2,3,4,7,8,9,11,12,14,15,16,17-dodecahydro-1H-cyclopenta [a]phenanthren-3-ol isorhamnetin 3,9-di-O-methylnissolin 7-O-methylisomucronulatol 9,10-dimethoxypterocarpan-3-O-β-D-glucoside (6aR,11aR)-9,10-dimethoxy-6a,11a-dihydro-6H-benzofurano [3,2-c]chromen-3-ol Bifendate formononetin Calycosin kaempferol FA isomucronulatol-7,2’-di-O-glucosiole 1,7-Dihydroxy-3,9-dimethoxy pterocarpene quercetin	55.38 50.83 36.91 36.23 49.6 53.74 74.69 36.74 64.26 31.1 69.67 47.75 41.88 68.96 49.28 39.05 46.43	0.78 0.29 0.75 0.78 0.31 0.48 0.3 0.92 0.42 0.67 0.21 0.24 0.24 0.71 0.62 0.48 0.28
*guizhi*	MOL001736 MOL000358 MOL000359 MOL000492 MOL000073 MOL004576 MOL011169	(-)-taxifolin beta-sitosterol sitosterol (+)-catechin ent-Epicatechin taxifolin Peroxyergosterol	60.51 36.91 36.91 54.83 48.96 57.84 44.39	0.27 0.75 0.75 0.24 0.24 0.27 0.82
*daxueteng*	MOL007920 MOL000359 MOL000358 MOL000096 MOL007923	meso-1,4-Bis-(4-hydroxy-3-methoxyphenyl)-2,3-dimethylbutane sitosterol beta-sitosterol (-)-catechin 2-(4-hydroxyphenyl)ethyl (E)-3-(4-hydroxyphenyl)prop-2-enoate	31.32 36.91 36.91 49.68 93.36	0.26 0.75 0.75 0.24 0.21
*dilong*		Crocin Gardenoside Ursolic Acid Geniposide Dulcitol Gardnerilin A D-Mannitol Enoxaparin Crocetin Heparin Nadroparin D-Mannoheptulose L-Galactoheptulose	*p *< 0.05 *p *< 0.05 *p *< 0.05 *p *< 0.05 *p *< 0.05 *p *< 0.05 *p *< 0.05 *p *< 0.05 *p* < 0.05 *p *< 0.05 *p *< 0.05 *p* < 0.05 *p *< 0.05	
*shuizhi*		Xanthine Guanidine Guanosine Xanthinin 4-Guanidino-1-Butanol Hyrcanoside Cholesterol	*p *< 0.05 *p *< 0.05 *p* < 0.05 *p *< 0.05 *p *< 0.05 *p *< 0.05 *p *< 0.05	

### Collection of target genes associated with DCM

**Fig 2:**
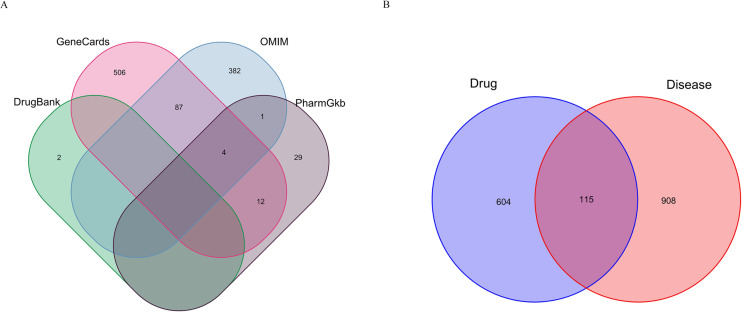
Identification of the core targets for ZL against DCM. (A) Compilation (A) Compilation of DCM-related predicted targets. (B) Venn diagram depicting the intersection of targets associated with ZL and those related to DCM.

A total of 4524 targets were identified from the GeneCards database, with 609 targets having a relevance score of ≥ 10. Furthermore, 474 targets were sourced from the OMIM database, 46 targets from the PharmGkb database, and 2 from the DrugBank database. After de-duplication, a total of 1023 disease-related targets were obtained (Fig 2A).

### Screening of potential targets for ZL treatment of DCM

Utilizing the “Venn” package in R4.2.2 software, the drug targets of ZL were intersected with the disease-related targets of DCM, yielding a total of 115 overlapping targets for the treatment of DCM with ZL. This finding suggests potential therapeutic applications of ZL for DCM (Fig 2B).

### The drug-target network interactions

**Fig 3:**
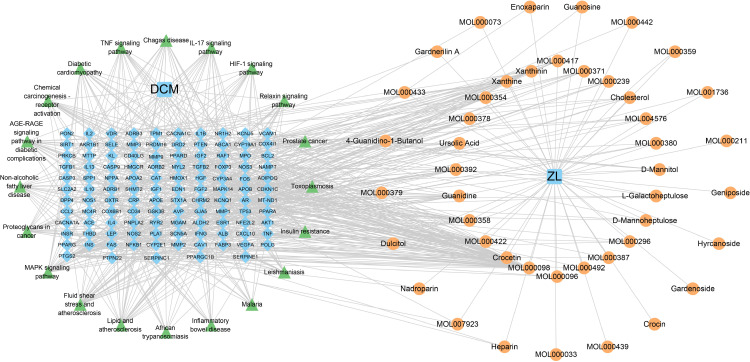
The drug-target network interactions for ZL against DCM. The The drug-target network comprises 166 nodes and 298 edges. The blue diamond-shaped nodes represent potential targets of DCM. Within the blue square nodes, HQ stands for *huangqi*, GZ for *Guizhi*, SZ for *shuizhi*, DL for *dilong*, DXT for *daxueteng*, and CF represents the common component shared by *guizhi* and *daxueteng*. The external magenta-colored nodes reflect the 45 bioactive substances of ZL. The larger the node size, the greater the degree of the bioactive compound within the network.

To illustrate the relationship between compounds and predicted overlapping targets, we utilized Cytoscape to construct a drug-target network interactions. 115 potential targets and 45 biologically active compounds were entered into Cytoscape to construct a network of biologically active targets. The network analyzer was used to identify the number of nodes and edges in the network. The constructed network has 166 nodes and 298 edges (Fig 3). The greater the degree of bioactive compounds, the greater the size of the node.The highest levels of bioactive compounds in ZL against DCM were Quercetin, Crocetin, Kaempferol, Guanidine, Formononetin and 7-O-methylisomucronulatol, which were associated with 50, 27, 19, 18, 13 and 13 genes respectively. It clearly demonstrates the potential of these bioactive compounds to be key components of ZL for the treatment of DCM.

### Construction of the PPI network and screening of Hub targets

**Fig 4:**
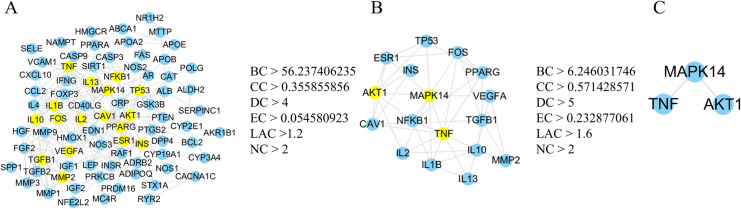
Hub gene target screening process. **(A)** (A) PPI network of predicted targets for the treatment of DCM using ZL. (B) Key proteins from the PPI network were extracted from (A). (C) Three key proteins for the treatment ofDCM were extracted from (B).

In order to analyze the function between known and predicted proteins, the identified 115 intersecting targets were incorporated into the String website to construct a PPI network. The connecting lines between the nodes in the graph represent interactions between targets, with colors signifying interactions and a higher number of connecting lines denoting closer interactions. In the constructed network, there were 115 nodes and 234 edges, with an average node degree of 4.07 and an average local clustering coefficient of 0.394. In addition, the PPI data in tsv format were downloaded and imported into Cytoscape v3.9.1 software. Next, CytoNCA was employed to perform a topological analysis of the network, and the R language procedures were executed twice. The criteria for the first round of screening were BC > 56.237406235, CC > 0.355855856, DC > 4, EC > 0.054580923, LAC > 1.2, NC > 2, whereas the second round used criteria of BC > 6.246031746, CC > 0.571428571, DC > 5, EC > 0.232877061, LAC > 1.6, NC > 2. Finally, a total of three core targets were identified, namely MAPK14, TNF and AKT1 as presented in Table 4 and Fig 4.

**Table 4:** Target parameters of ZL in the treatment of DCM.

Gene	Betweenness	Closeness	Dgree	Eigenvector	LAC	Network
MAPK14	42.47936508	0.695652174	9	0.395042151	2.444444444	5
TNF AKT1	38.51904762 23.64126984	0.695652174 0.592592593	9 7	0.395984441 0.283565849	2.444444444 2	5.416666667 4.166666667

### GO and KEGG enrichment analyses

**Fig 5:**
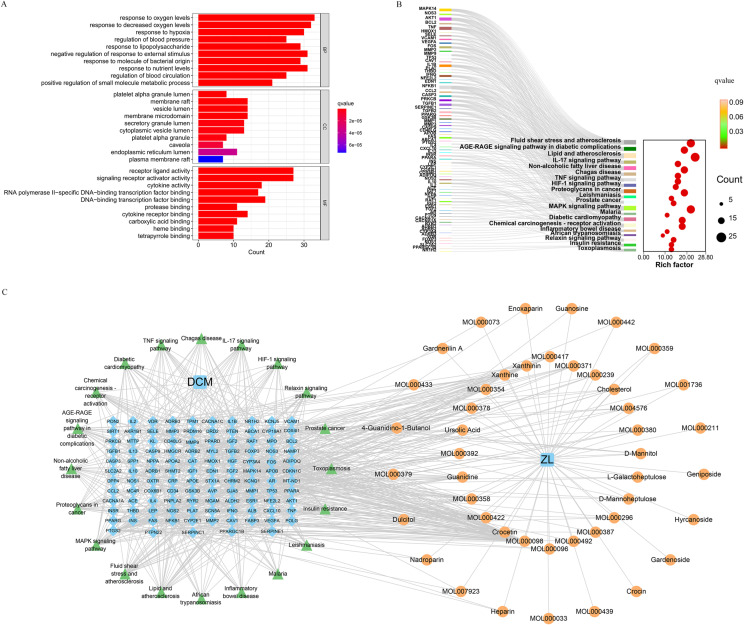
GO and KEGG pathway enrichment analyses. **(A)** (A) Top 10 results from GO enrichment analysis in Biological Process (BP), Cellular Component (CC), and Molecular Function (MF). (B) Sankey diagram of KEGG pathways for analysis of ZL targets in DCM therapy. The left rectangle represents the target, the right rectangle represents the target, and the line represents the pathway in which the target acts. (C) The drug-target-pathway network. Orange circles represent the bioactive components of ZL, blue diamonds represent cross-targets, and green triangles represent the top 20 KEGG pathways..

**Fig 6:**
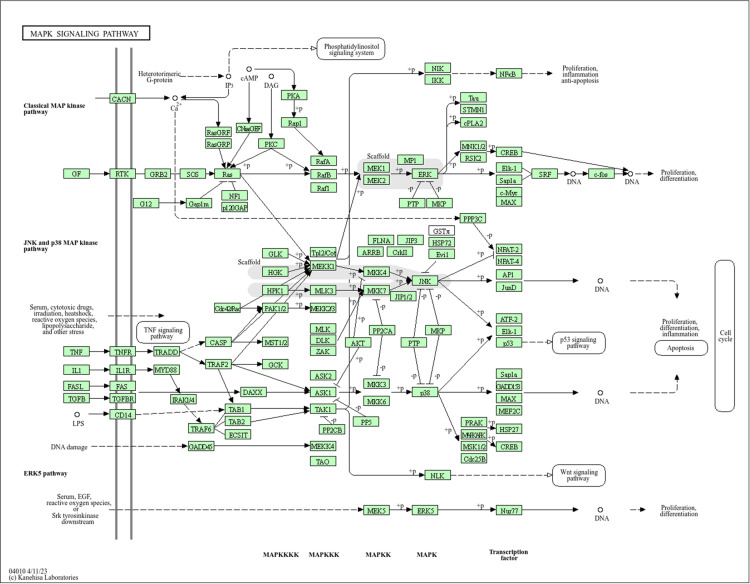
Distribution of targets in the MAPK signaling pathway.

GO and KEGG analyses were performed on the 115 intersecting targets utilizing the “cluster Profiler” and “enrichplot” package in R 4.2.2 software, the filtering condition was q-value ≤ 0.05. The GO analysis revealed 2616 biological processes (BP), predominantly enriched in responses to oxygen levels, decreased oxygen content, and hypoxia, among others. Furthermore, 50 cellular components (CC) were enriched, primarily including membrane rafts, vesicle lumens, and membrane microregions. Additionally, 156 molecular functions (MF) were enriched, related to receptor-ligand activity, signaling receptor activator activity, and DNA-binding transcription factor binding, among others. Finally, Selected the first 10 results to display (Fig 5A). Meanwhile, a total of 168 KEGG pathways were enriched. The top 20 pathways were selected to plot Sankey bubble plots, and the filtering condition was q-value ≤ 0.05 (Fig 5B). The KEGG pathway enrichment analysis demonstrated significant enrichment of the common targets in the MAPK, PI3K-Akt, AGE-RAGE, and IL-17 signaling pathways. We used Cytoscape v3.9.1 to construct a drug-target-pathway network between ZL bioactive compounds, cross-targets and the top 20 KEGG pathways (Fig 5C). The network consists of 182 nodes and 655 edges. We Visualized the MAPK signaling pathway (hsa04010) in KEGG enrichment analysis using the “pathview” package in R4.2.2 (Fig 6).

### Molecular docking

**Fig 7:**
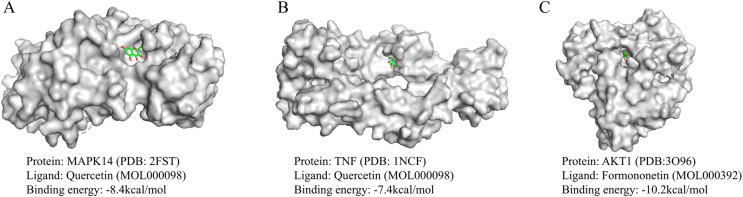
Molecular docking results for the main chemical components of ZL.

To validate the network pharmacology findings, molecular docking was used to assess the binding affinity between the screened compounds and the targets. Molecular docking simulations were performed based on three Hub genes and potential bioactive compound ligands (Quercetin, Kaempferol, Crocetin, Guanidine, Formononetin, 7-O-methylisomucronulatol) obtained from the PPI network. The binding energies (kcal/mol) of each compound ligand for all the selected targets are shown in Table 5. In addition, three representative models were selected for visualization (Fig 7). A lower intermolecular binding energy implies a higher binding affinity between molecules. Generally speaking, when the binding energy is below -1.2 kcal/mol, it is often considered indicative of good binding activity between the molecules [35].

**Table 5:** The binding energy (kcal/mol) of each phytoligand against all the selected targets.

Phytoconstituent name	MAPK14	TNF	AKT1
Quercetin Kaempferol Crocetin Guanidine Formononetin 7-O-methylisomucronulatol	-8.4 -8.3 -6.8 -3.5 -8.4 -6.7	-7.4 -7.1 -6.0 -3.5 -6.8 -7.1	-9.8 -9.5 -8.5 -4.1 -10.2 -8.4

### H&E staining and Masson staining

**Fig 8:**
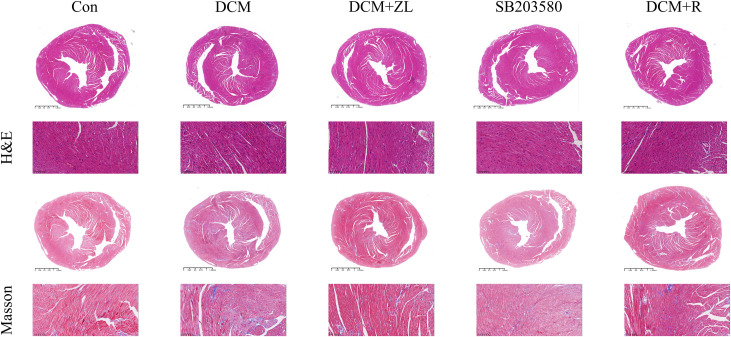
Effect of ZL on myocardial histopathologic changes in DCM mice. H H&E staining analysis and Masson staining analysis of the effect of ZL on myocardial tissue in DCM mice (X200).

H&E staining results illustrated that in the control group, the myocardial structure appeared normal, with an orderly arrangement of myocardial fibers and no evidence of swelling or rupture. In comparison to the control group, mice in the DCM group exhibited myocardial hypertrophy, accompanied by a disorganized arrangement of myocardial fibers. However, in the DCM + ZL, SB203580, and DCM + R groups, the degree of myocardial hypertrophy and fiber disarray was improved to varying degrees compared to the DCM group. Masson’s trichrome staining results revealed the absence of blue-stained collagen fibers in the myocardial interstitium of mice in the control group. In contrast, a higher amount of blue-stained collagen fibers was observed in the myocardial interstitium of mice in the DCM group compared to the control group. Furthermore, in the DCM + ZL, SB203580, and DCM + R groups, the presence of blue-stained collagen fibers in the myocardial interstitium was reduced to varying degrees compared to the DCM group (Fig 8). Taken together, these results demonstrated that ZL can mitigate DCM-induced myocardial fibrosis, thereby exerting protective effects on the heart.

### RT-qPCR

**Fig 9:**
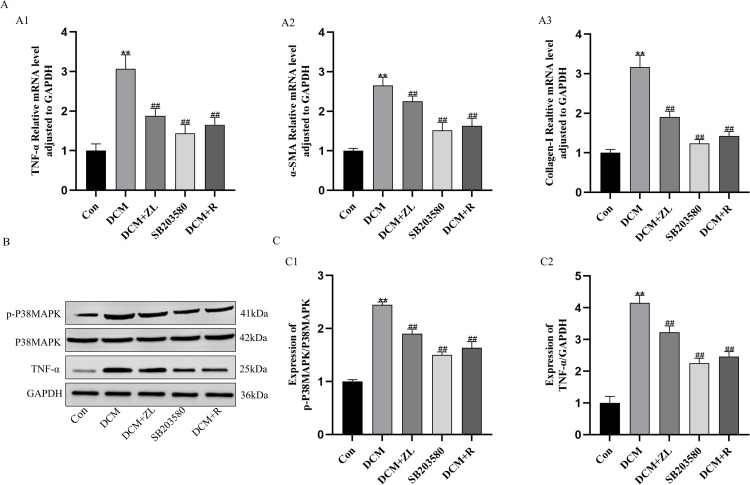
ZL up-regulates the expression of p-P38MAPK, TNF- **α,** **α-SMA, and Collagen-I in DCM cardiomyocytes.** **(A)** The relative (A) The relative mRNA expression level of TNF-α (A1), α-SMA (A2), Collagen-I (A3). (B - C) Western blot analysis of p-P38MAPK (C1), TNF-α (C2). Data are presented as means±SD, from 3 independent experiments. (*P < 0.05, **P < 0.01 compared with the control group; #P < 0.05, ##P < 0.01 compared with the DCM group, n = 3).

To investigate the impact of ZL on the expression of TNF-α, α-SMA, and collagen-I mRNA in the myocardial tissue of DCM mice, RT-qPCR was carried out to assess the mRNA expression levels in the myocardial tissue of mice from various groups. On the one hand, the mRNA expression levels of TNF-α, α-SMA, and Collagen-I were higher in the DCM group (*P* < 0.01) compared to the control group. On the other hand, the expression levels of TNF-α, α-SMA, and Collagen-I were significantly lower in the DCM + ZL, SB203580, and DCM + R groups compared to the DCM group (*P* < 0.01, Fig 9A).

### Western blot analysis

To investigate the effects of ZL on the expression of p-P38MAPK and TNF-α proteins in the myocardial tissues of DCM mice, Western blot analysis was performed. The results exposed that compared to the control group, the protein levels of p-P38MAPK and TNF-α were significantly higher in the myocardial tissues of DCM mice (P < 0.01). In contrast, compared to the DCM group, the expression levels of p-P38MAPK and TNF-α proteins were significantly lower in the DCM + ZL group, SB203580 group, and DCM + R group (*P* < 0.01, Fig 9B-9C).

### Immunofluorescence analysis

**Fig 10:**
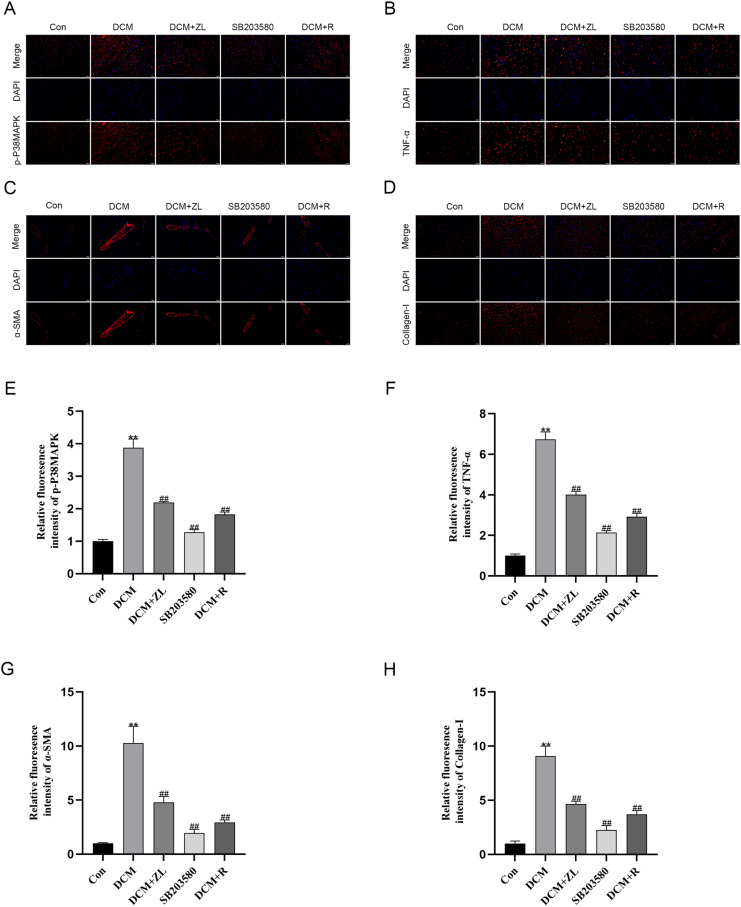
The effect of ZL on myocardial tissue fibrosis in DCM mice. (A) Immunofluorescence analysis of p-P38MAPK expression in DCM mice (X400). (B) Immunofluorescence analysis of TNF-α expression in DCM mice (X400). (C) Immunofluorescence analysis α-SMA expression in DCM mice (X400). (D) Immunofluorescence analysis of Collagen-I expression in DCM mice (X400). (E-H) Quantitative analysis of immunofluorescence of p-P38MAPK, TNF-α, α-SMA, and Collagen-I expression in myocardial tissue. Data are presented as means±SD, from 3 independent experiments. (*P < 0.05, **P < 0.01 compared with the control group; #P < 0.05, ##P < 0.01 compared with the DCM group, n = 3).

Immunofluorescence analysis was performed to examine the protein expression levels of p-P38MAPK, TNF-α, α-SMA, and Collagen-I in the myocardial tissues of mice in each group. As anticipated, the results showed that the protein levels of p-P38MAPK, TNF-α, α-SMA, and Collagen-I in the myocardial tissues of the DCM group were significantly higher compared to the control group. Meanwhile, the protein expression levels of p-P38MAPK, TNF-α, α-SMA, and Collagen-I were significantly lower in the DCM + ZL, SB203580, and DCM + R groups compared to the DCM group (*P* < 0.01, Fig 10A-10E). These findings collectively suggested that the treatment of DCM with ZL may be associated with the downregulation of these inflammatory and fibrosis-related markers.

## Discussion

Despite significant advances in the treatment of DCM, its intricate and complex pathogenesis has resulted in a scarcity of interventions that can produce significant therapeutic effects. Traditional Chinese medicine, with its unique advantages of multiple components, targets, and pathways, has shown promising therapeutic effects for DCM. To the best of our knowledge, this is the first study to uncover the cardioprotective effects of ZL in a DCM mouse model, specifically highlighting its efficacy in alleviating myocardial fibrosis. Furthermore, a protein-protein interaction network was constructed for ZL in the treatment of DCM, and an in-depth pathway enrichment analysis was conducted. Importantly, the results of the animal experiments preliminarily validated the key targets of ZL in the treatment of DCM, providing valuable insights for unraveling the therapeutic mechanisms of ZL on DCM.

DCM is a complication of diabetes, with myocardial fibrosis being its primary pathological mechanism. Herein, significant collagen accumulation and fibrosis were observed in the myocardium of mice with diabetic cardiomyopathy. However, treatment with ZL resulted in a marked improvement and reversal of these pathological changes. According to a previous study, ZL can modulate the PI3K-AKT and AGE-RAGE signaling pathways, leading to the upregulation of the anti-inflammatory factor IL-10 expression and concomitant downregulation of the expression of the pro-inflammatory factors Cox-2 and the adhesion molecule ICAM-1. This protective effect of ZL against hypertension-induced myocardial fibrosis enhances myocardial structure and function [32]. ZL, in combination with atorvastatin, can inhibit the activation of the NF-κB/NLRP3 signaling pathway and limit the release of p-NF-κB, NLRP3, caspase-1, IL-1β, and IL-18, thereby alleviating vascular inflammation and significantly attenuating atherosclerosis in rabbits [36]. Interestingly, ZL effectively alleviates vascular endothelial cells in atherosclerosis by regulating the miR-30b-5p/NLRP3 axis and significantly reduces the AS-related mRNA expression levels of NLRP3, ASC, Caspase 1, IL-1 β, and IL-18 [17]. Our study conjointly demonstrates that ZL can down-regulate the expression of p-P38MAPK, TNF-α, α-SMA, and Collagen-I, thereby relieving myocardial fibrosis in mice with DCM, consistent with the findings of earlier studies.

P38MAPK is an integral component of the mitogen-activated protein kinase (MAPK) signaling pathway, encompassing four homologous proteins: P38α (also known as MAPK14), P38β, P38γ, and P38δ [37]. Of note, this pathway can be activated by various inflammatory stimuli, including tumor necrosis factor (TNF) and interleukin-1 [38]. Previous studies have demonstrated that the p38MAPK signaling pathway is intimately associated with diabetic cardiomyopathy, myocardial interstitial fibrosis, cardiomyocyte apoptosis, oxidative stress, and microvascular pathology [39]. In diabetic conditions, abnormal activation of p38MAPK is observed, and inhibiting its activity may delay the progression of diabetic cardiomyopathy [40]. Notably, in mouse models of diabetic cardiomyopathy, elevated expression levels of TNF-α and p-P38MAPK were noted. Suppression of the p38MAPK pathway by nesfatin-1 has been reported to ameliorate diabetic-induced cardiac damage [41]. In diabetic cardiomyopathy, factors such as hyperglycemia and insulin resistance may exacerbate inflammatory responses. Meanwhile, inflammation can cause degeneration and necrosis of cardiomyocytes, impair the function of vascular endothelial cells, and activate and proliferate fibroblasts, eventually leading to myocardial interstitial fibrosis [42]. TNF-α emerges as a pivotal inflammatory cytokine that plays a determinant role in inflammatory responses. Prior research evinced that TNF-α mediates multiple pathological processes in the myocardium through inflammatory reactions [43]. Specifically, TNF-α promotes inflammatory damage in tissues by activating upstream MKK3 and MKK4, thereby facilitating the phosphorylation and subsequent damage of the p38MAPK signaling pathway [44]. It is worthwhile emphasizing MK2 and MK3 double-knockout down-regulates both TNF synthesis and P38MAPK expression levels in mice [45]. Studies unveiled that inhibiting p38MAPK activity in diabetic cardiomyopathy mouse models effectively lowers the levels of inflammatory cytokines such as TNF-α, IL6, IL1-β, and TGF-β1 in myocardial tissue, thus improving the left ventricular function of DCM mice [33]. Herein, network pharmacology analysis identified the MAPK signaling pathway as one of the critical pathways in the therapeutic effect of ZL on DCM, with P38MAPK and TNF-α emerging as key targets. Our findings corroborated the phosphorylation of P38MAPK and the upregulated expression of TNF-α in DCM mice. Administration of ZL suppressed the activation of P38MAPK and the expression of its upstream inflammatory factor, TNF-α. Our results suggest that ZL’s effects on DCM are associated with the P38MAPK signaling pathway.

Alpha-smooth muscle actin (α-SMA) is a cytoskeletal protein and a well-established marker of myofibroblasts. In response to cardiac tissue damage or inflammatory stimuli, fibroblasts can differentiate into myofibroblasts and express α-SMA [46]. Noteworthily, myofibroblasts in cardiac tissue secrete and synthesize high amounts of extracellular matrix (ECM) [47]. Collagen Type I (Collagen-I) is the most abundant protein in the ECM of nearly all human tissues, including the myocardium [48]. Excessive deposition of collagen and ECM in damaged hearts leads to myocardial fibrosis, disrupting myocardial structure and impairing cardiac function [49]. Studies have shown that a significant reduction in the expression levels of α-SMA and Collagen-I in fibroblasts and proliferative scar tissue can be achieved by inhibiting the activity of p38MAPK [50]. This suggests that p38MAPK plays a critical role in regulating the synthesis of these fibrotic-related proteins, and thus, inhibiting its activity may serve as a promising anti-fibrotic therapeutic strategy. This study noted that ZL administration downregulated the expression of α-SMA and Collagen-I in diabetic cardiomyopathy mice, thereby alleviating myocardial fibrosis and preserving cardiac function.

## Conclusion

This research identified a close association between P38MAPK and DCM. Activation of the P38MAPK signaling pathway significantly aggravated inflammation and fibrosis in DCM cardiomyocytes. However, ZL administration ameliorated myocardial damage in DCM, with its effects partially mediated by the P38MAPK signaling pathway. Future studies are necessitated to elucidate the detailed mechanisms of ZL in the treatment and prevention of DCM. Nevertheless, our study identified ZL as a potential strategy for the prevention and treatment of DCM, warranting further investigation.

## Supporting information

S1 Data. (XLSL)

S2 Raw images. (PDF)

S3 R PDF. (PDF)
