# Supplementary material for: Network pharmacology and experimental validation to investigate the mechanism of action of Zhilong Huoxue Tongyu capsule in the prevention and treatment of diabetic cardiomyopathy
Source: PLoS One. 2025 May 15;20(5):e0323745. doi: 10.1371/journal.pone.0323745 (PMC12080927; doi:10.1371/journal.pone.0323745)
Supplement: S2 — (PDF) [file pone.0323745.s002.pdf]

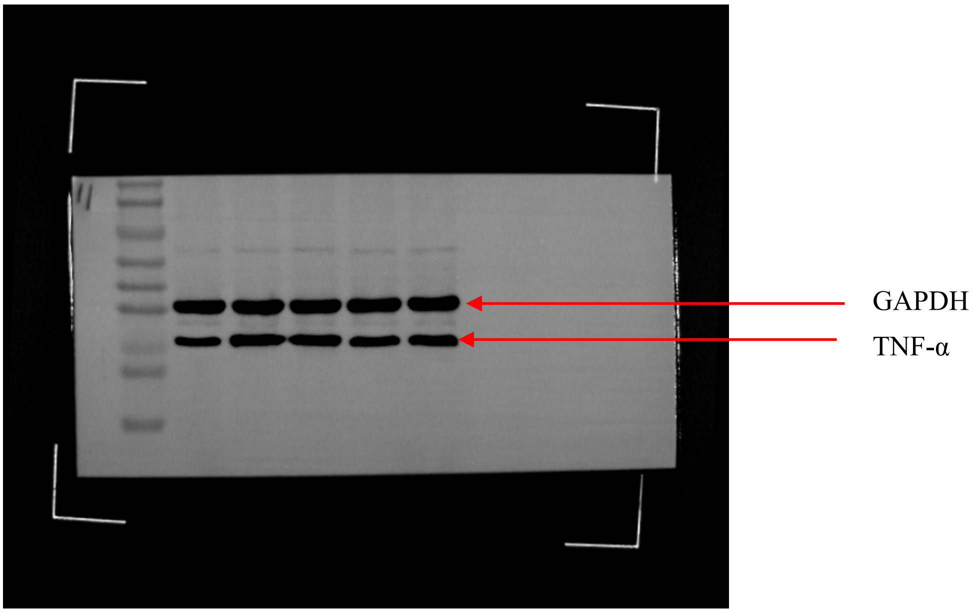
GAPDH
TNF-α

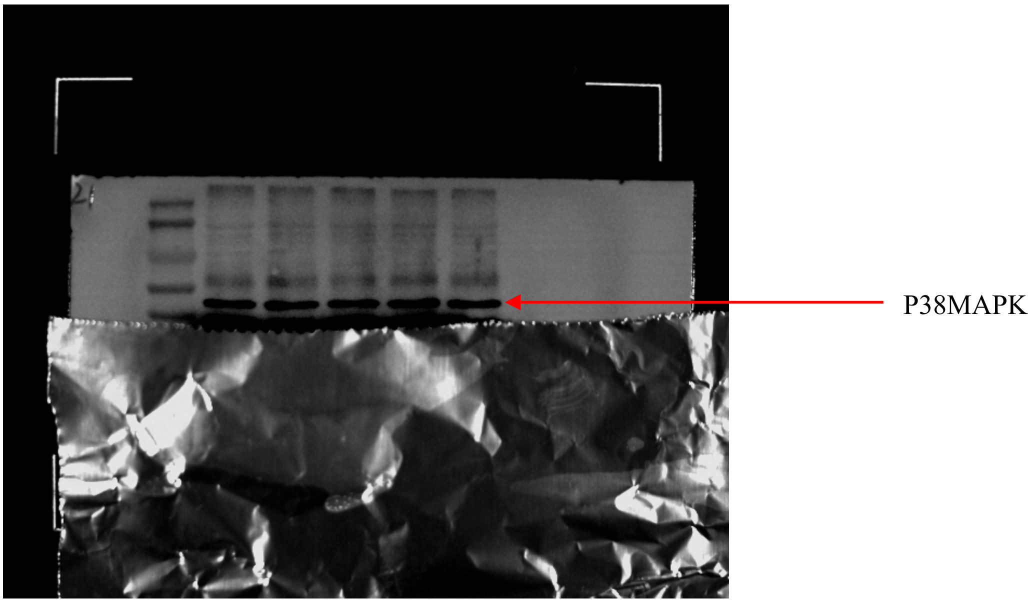
P38MAPK

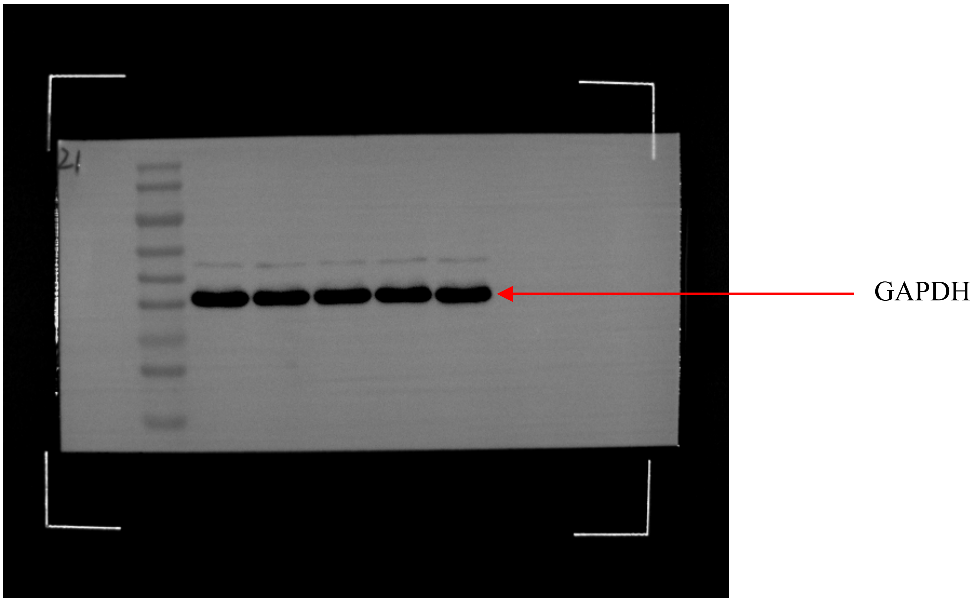
GAPDH

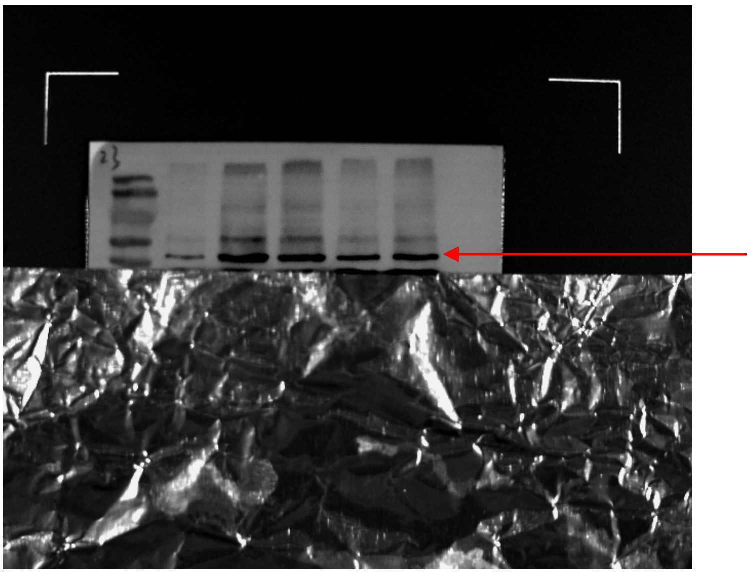

P-P38MAPK

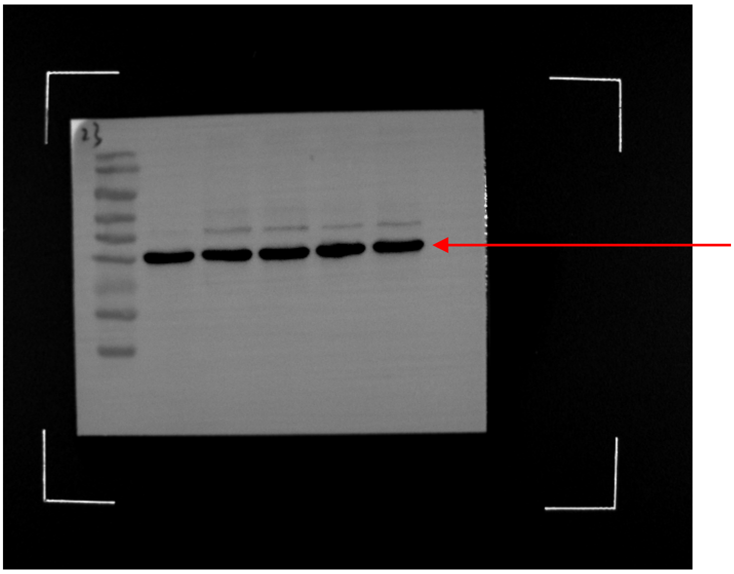

GAPDH

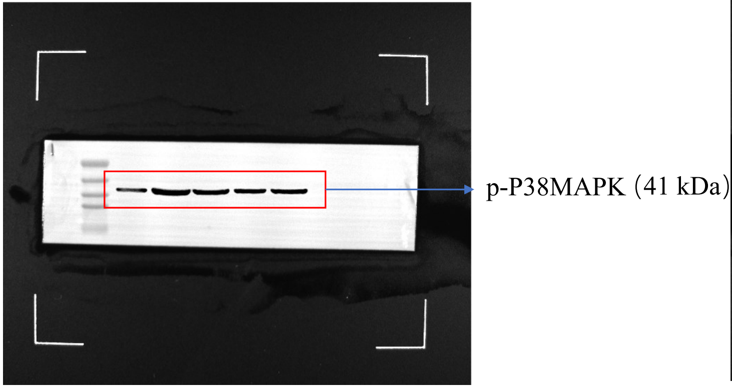

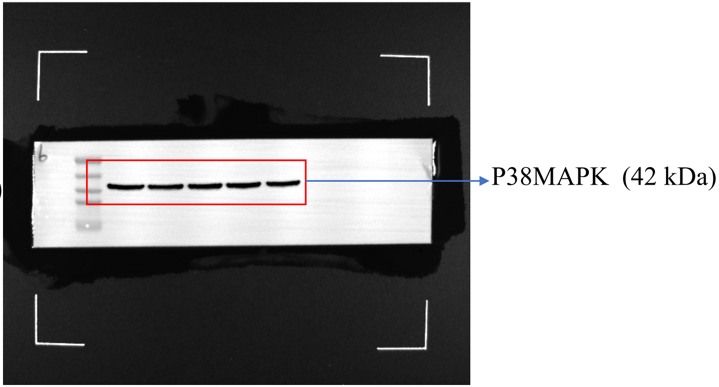

The labeled places represent p-P38MAPK/P38MAPK in the western blot analysis of Fig6

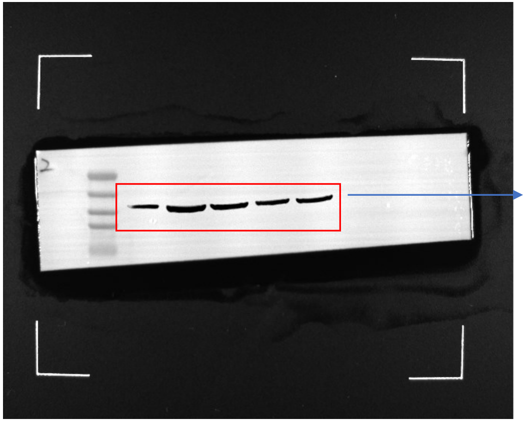

p-P38MAPK (41 kDa)

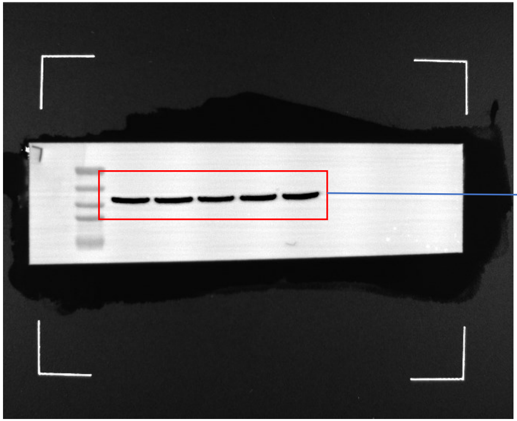

P38MAPK（42 kDa）

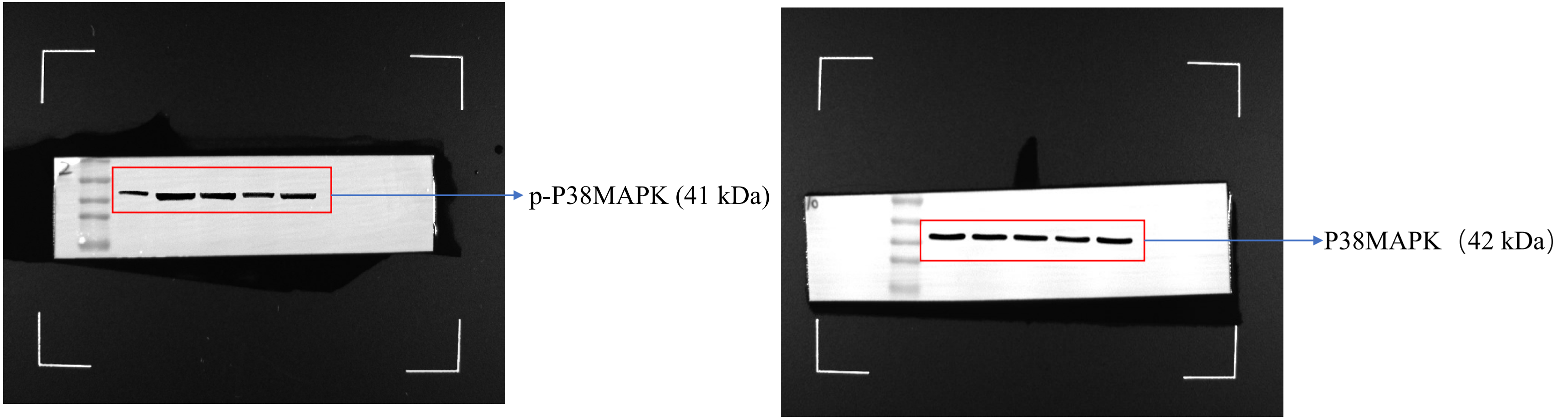
p-P38MAPK (41 kDa)
P38MAPK （42 kDa）

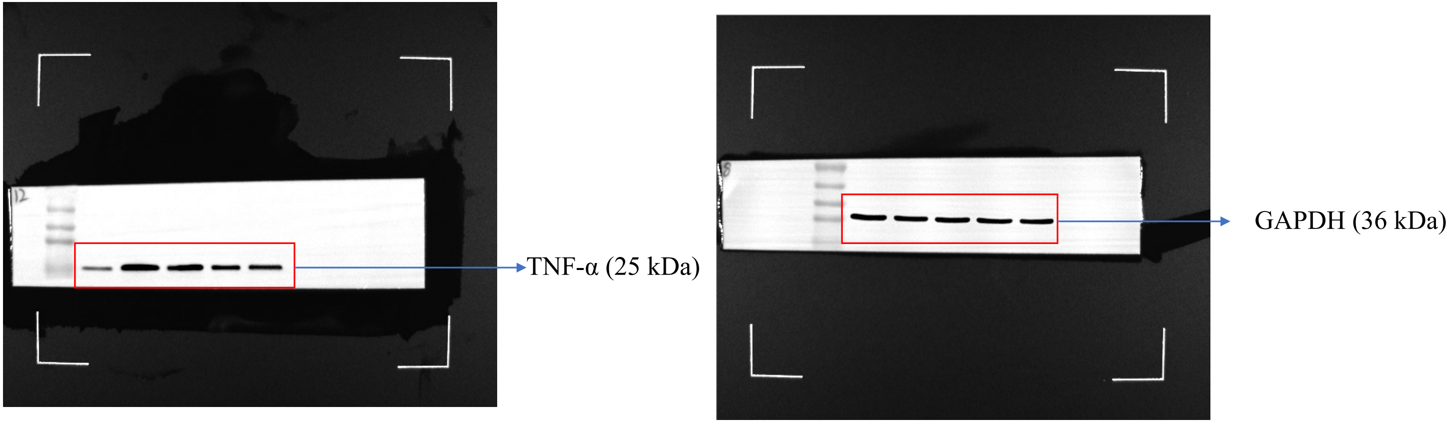

The labeled places represent TNF-α in the western blot analysis of Fig6

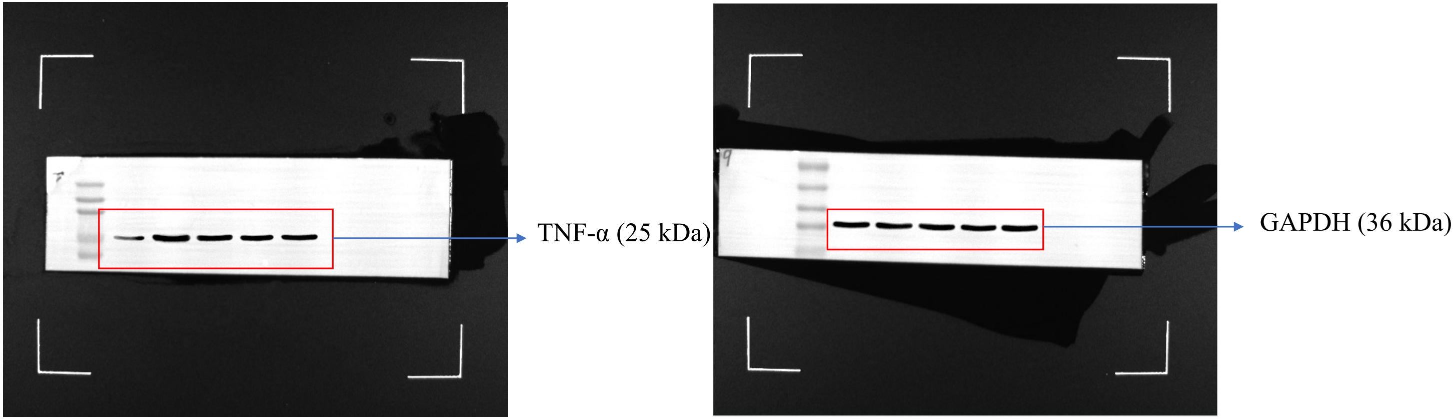
TNF-α (25 kDa)
GAPDH (36 kDa)

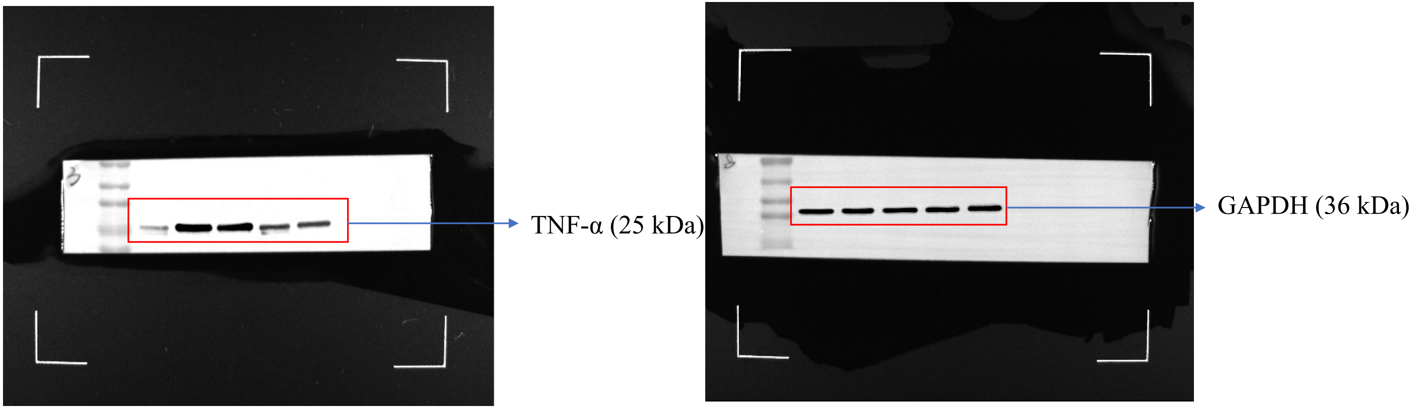
TNF-α (25 kDa)
GAPDH (36 kDa)

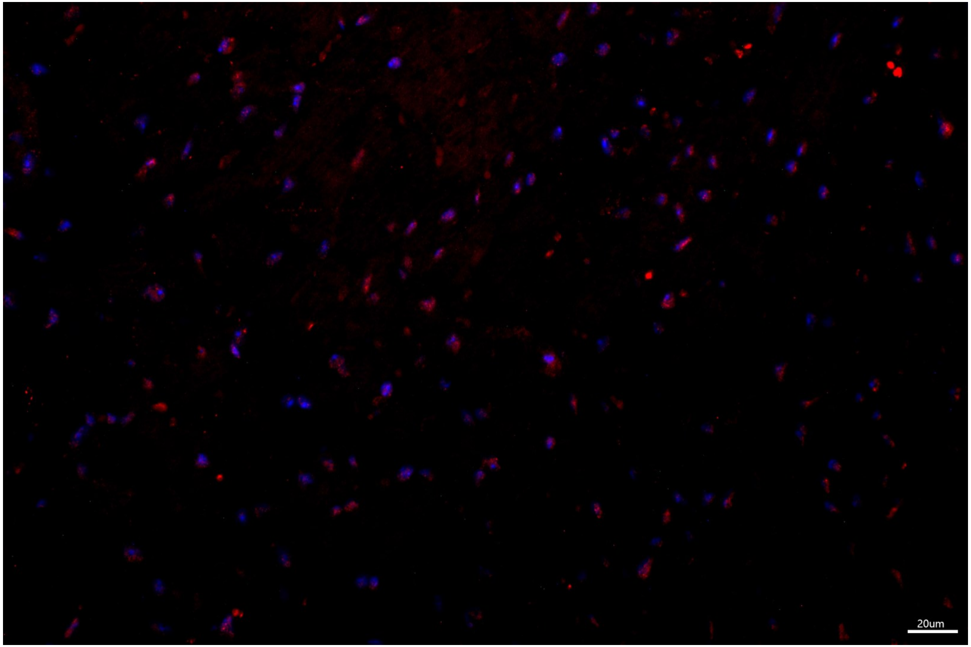

Original Image for Fig 10

(Con, Merge)

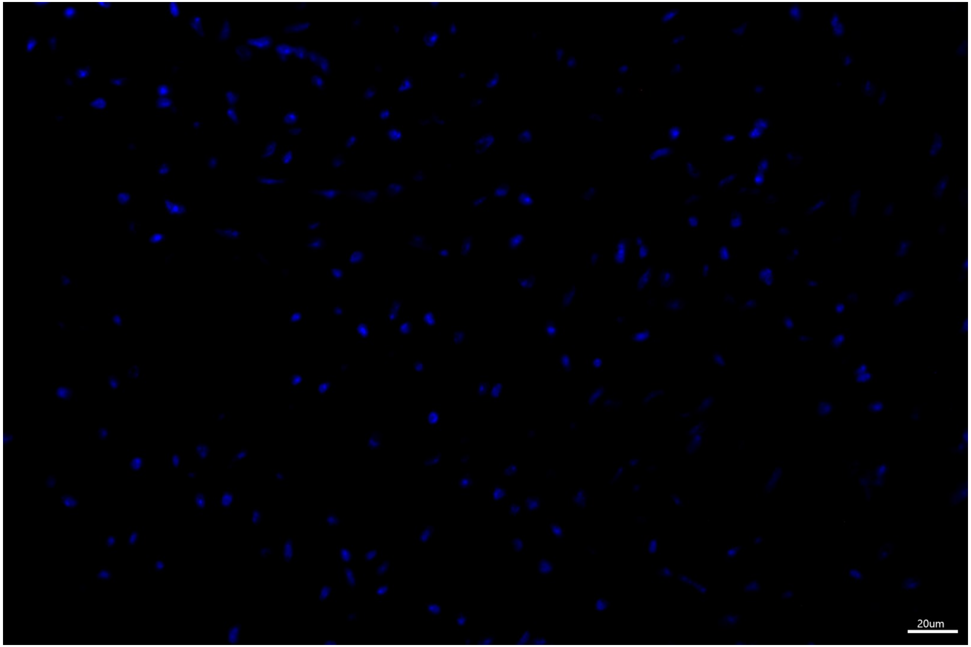

Original Image for Fig 10

(Con, DAPI)

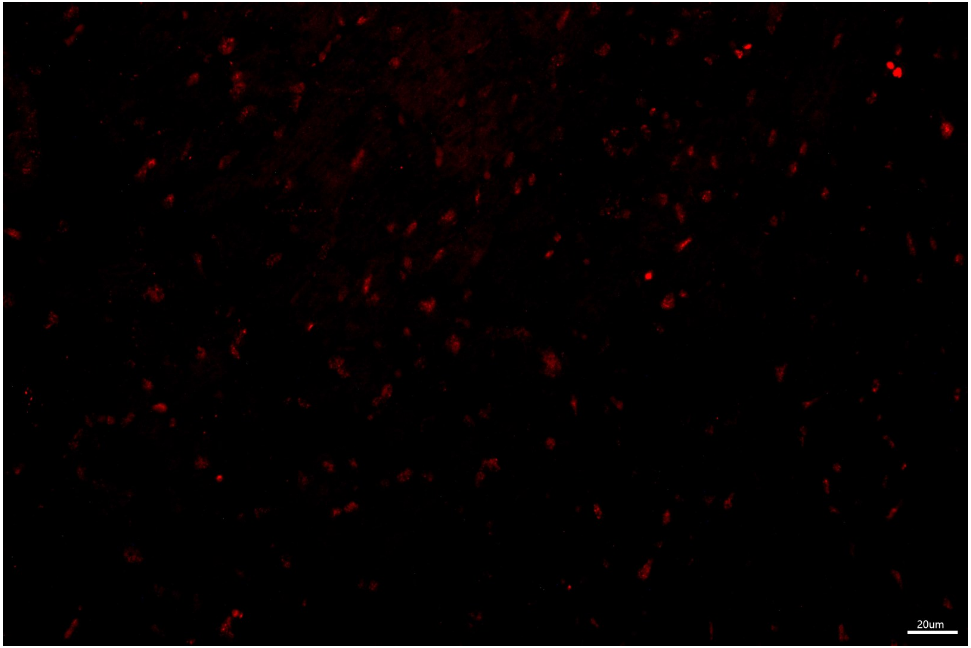

Original Image for Fig 10

(Con, p-P38MAPK)

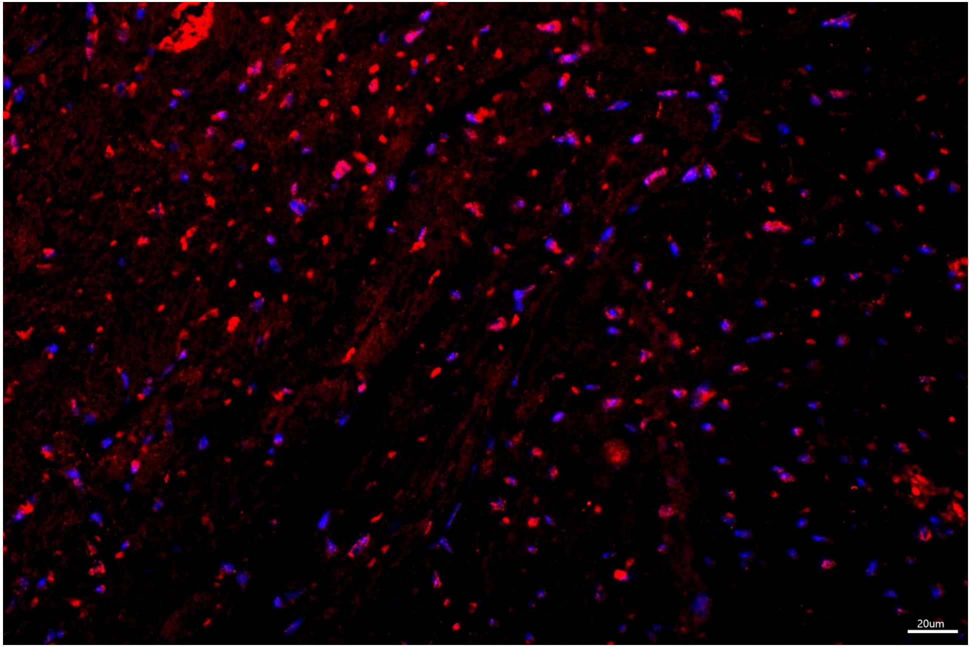

Original Image for Fig 10

(DCM, Merge)

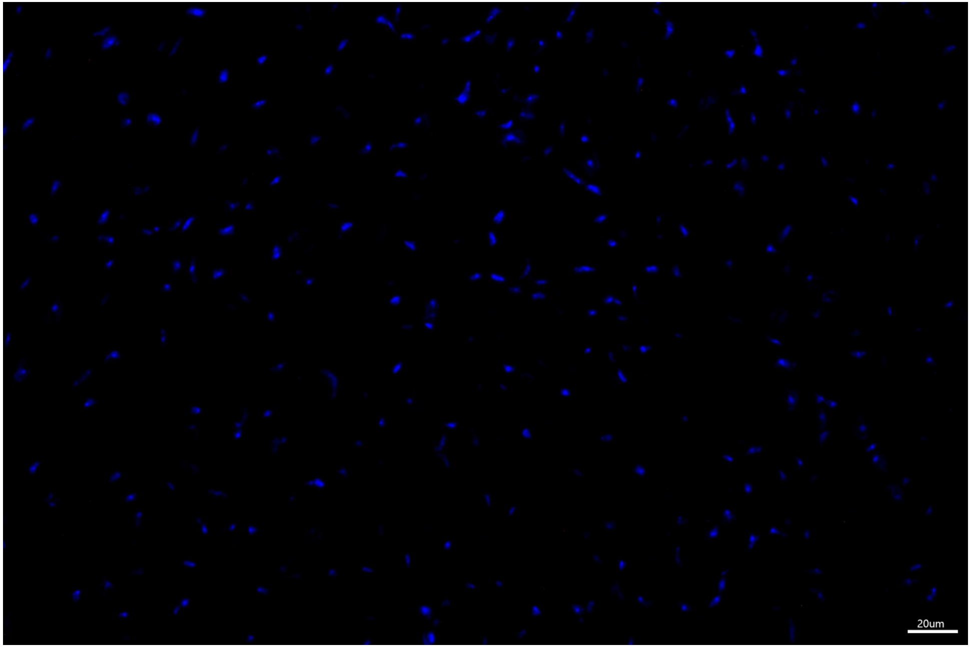

Original Image for Fig 10

(DCM, DAPI)

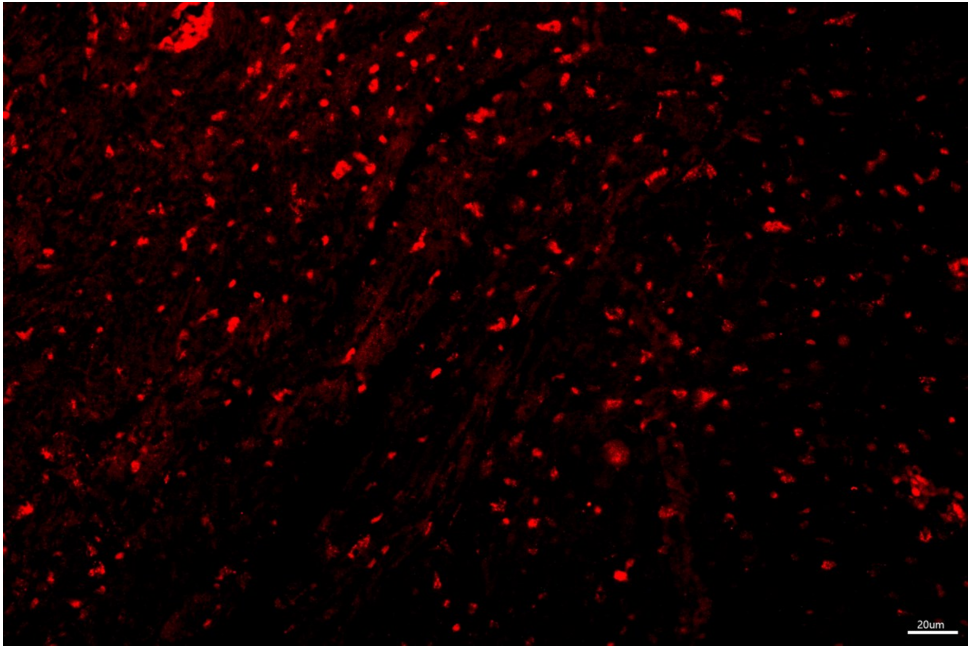

Original Image for Fig 10

(DCM, p-P38MAPK)

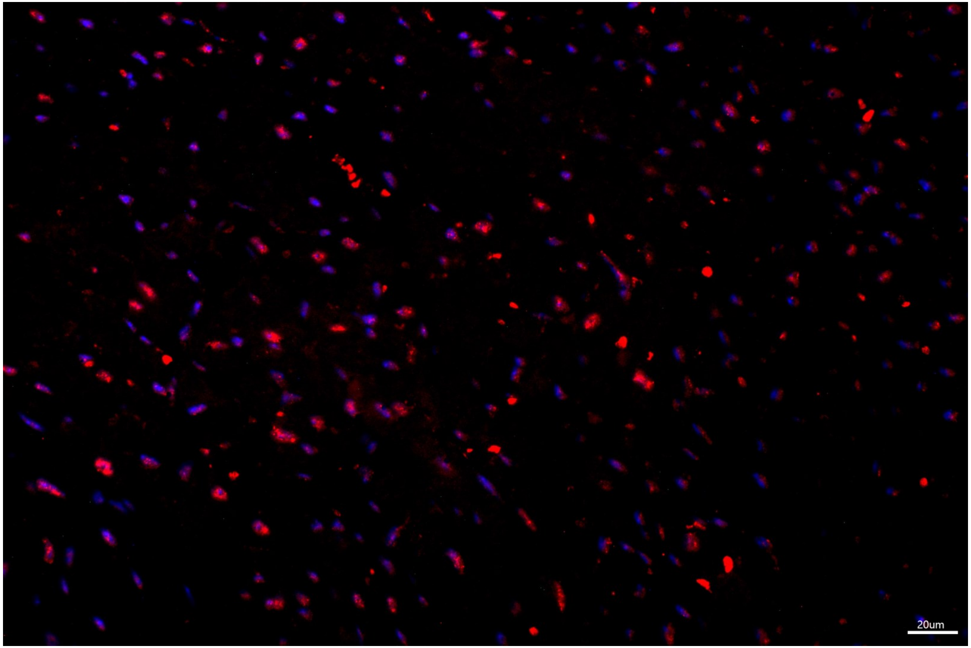

Original Image for Fig 10

(DCM+ZL, Merge)

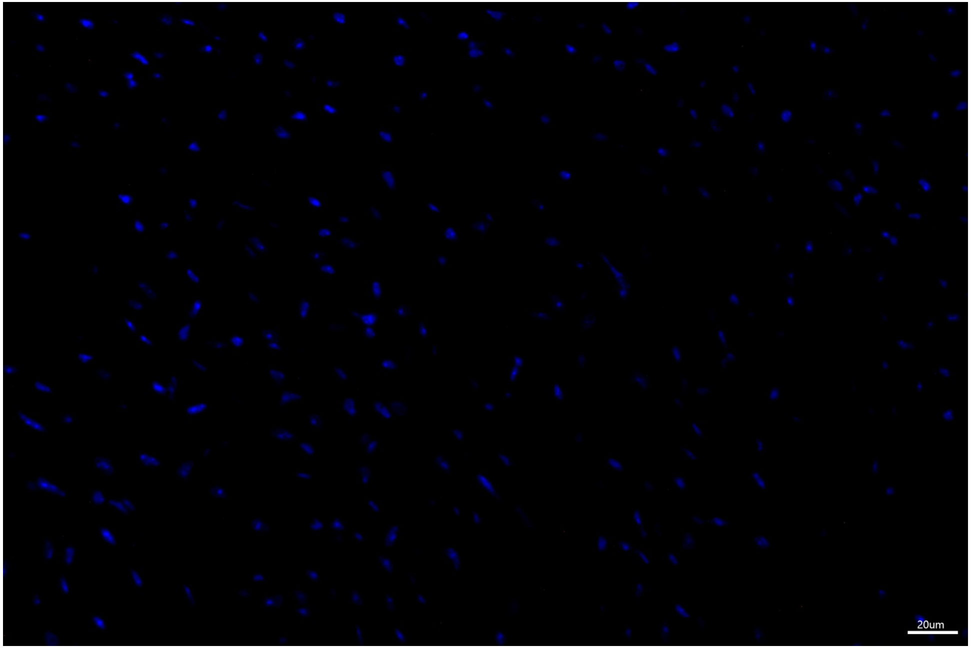

Original Image for Fig 10

(DCM+ZL, DAPI)

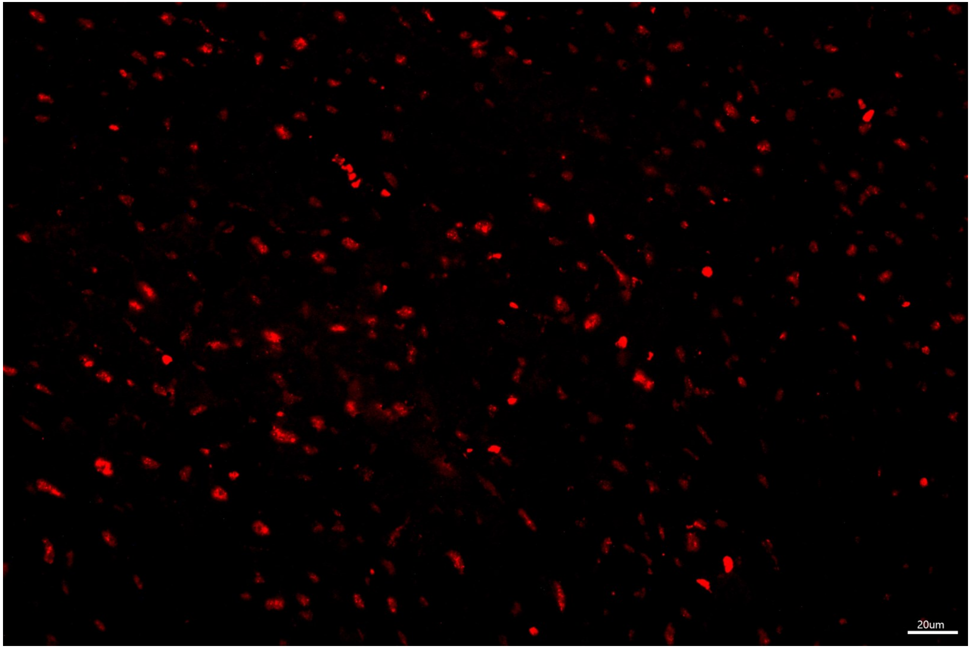

Original Image for Fig 10

(DCM+ZL, p-P38MAPK)

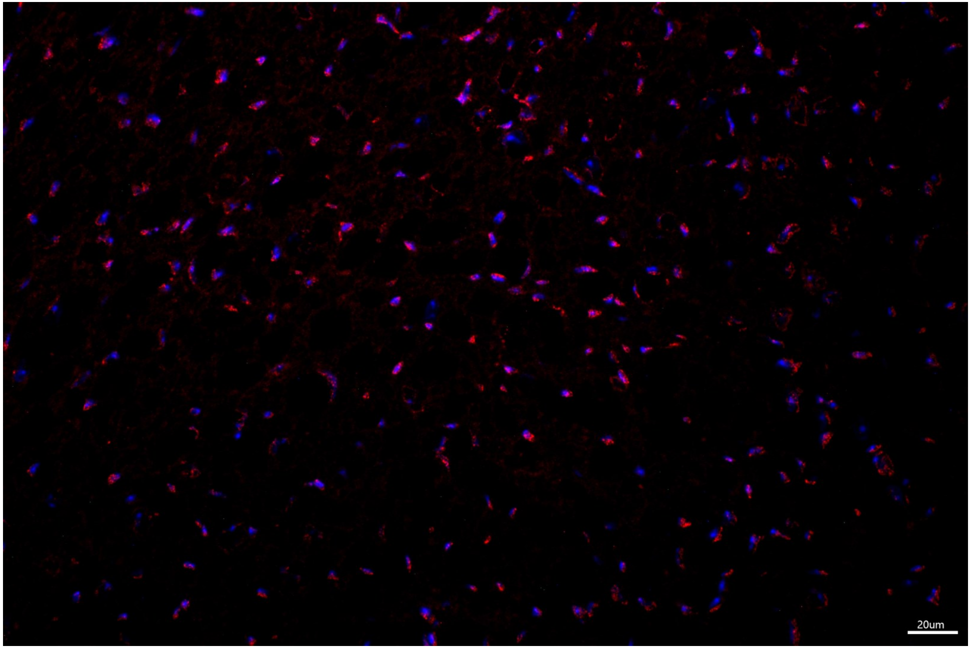

Original Image for Fig 10
(SB203580, Merge)

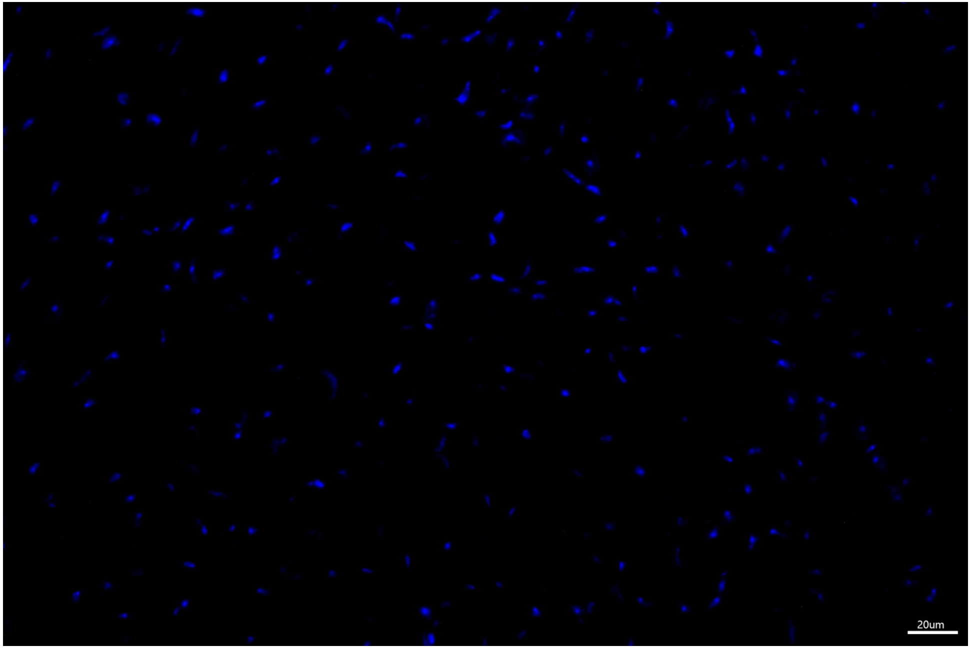

Original Image for Fig 10

(SB203580, DAPI)

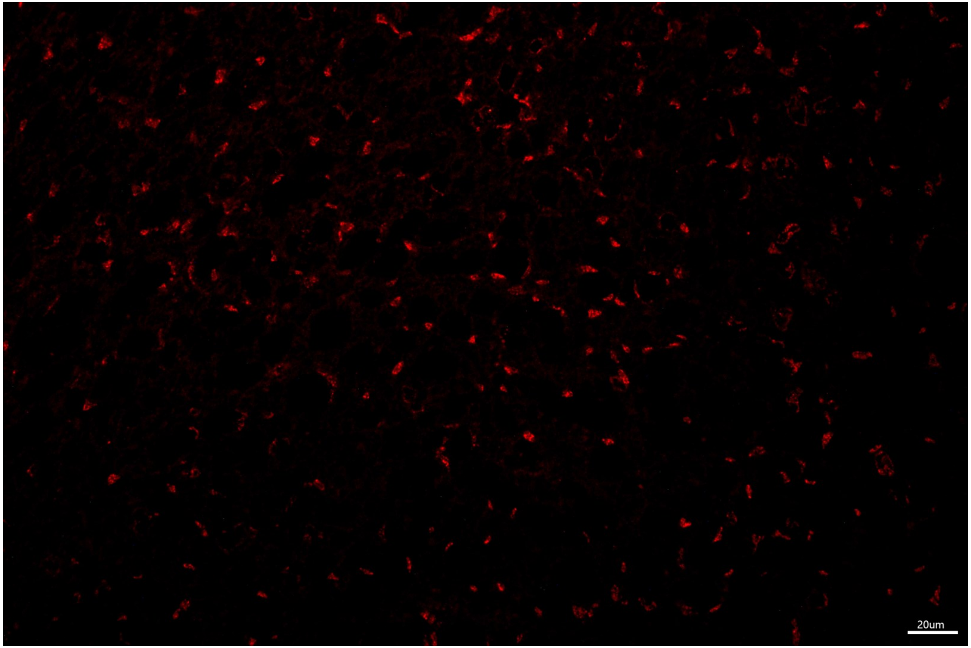

Original Image for Fig 10

(SB203580, p-P38MAPK)

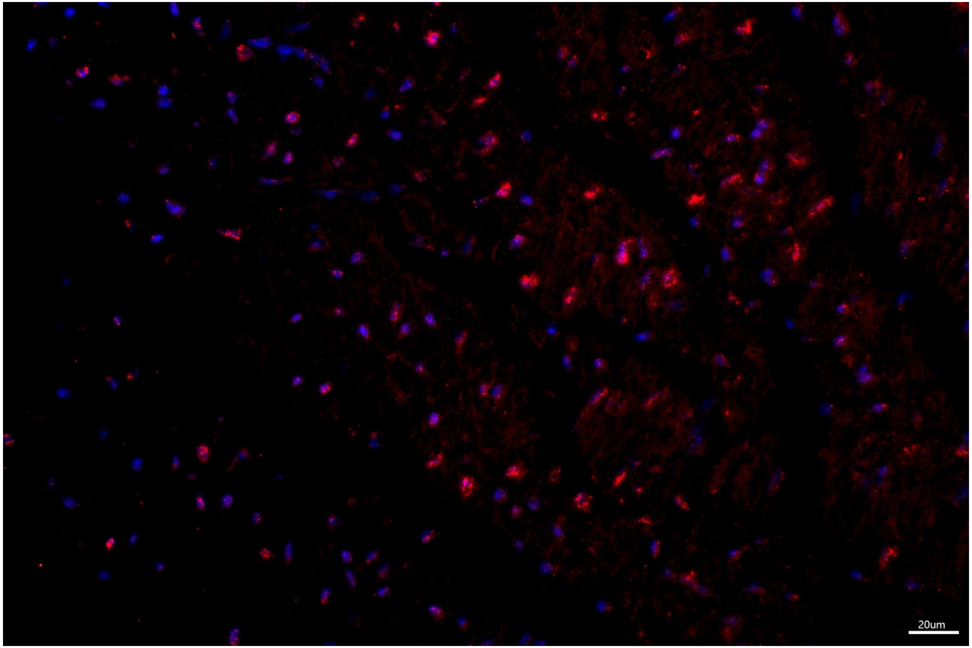

Original Image for Fig 10

(DCM+R, Merge)

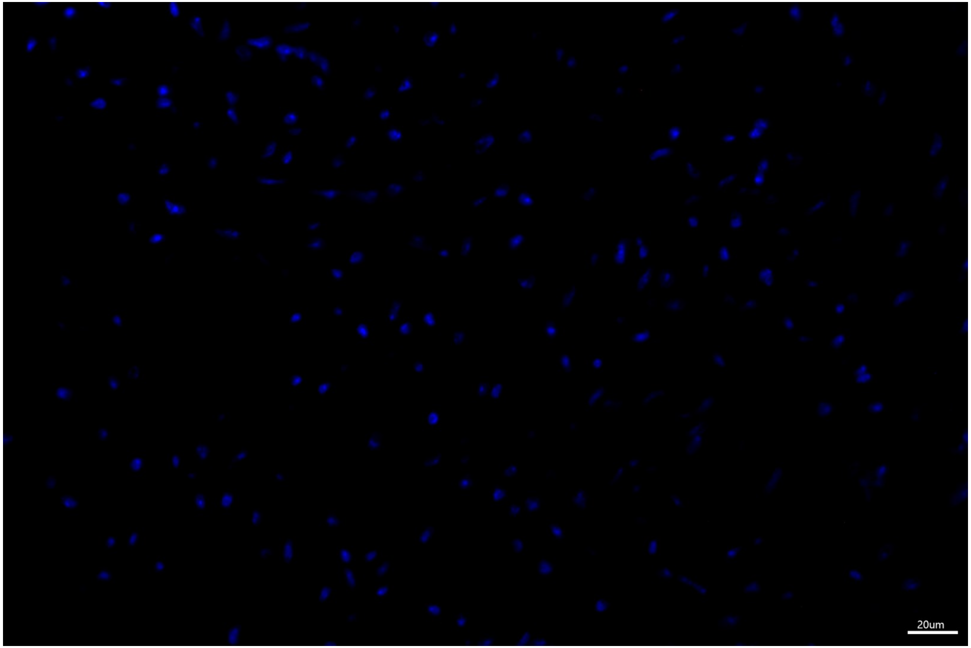

Original Image for Fig 10

(DCM+R, DAPI)

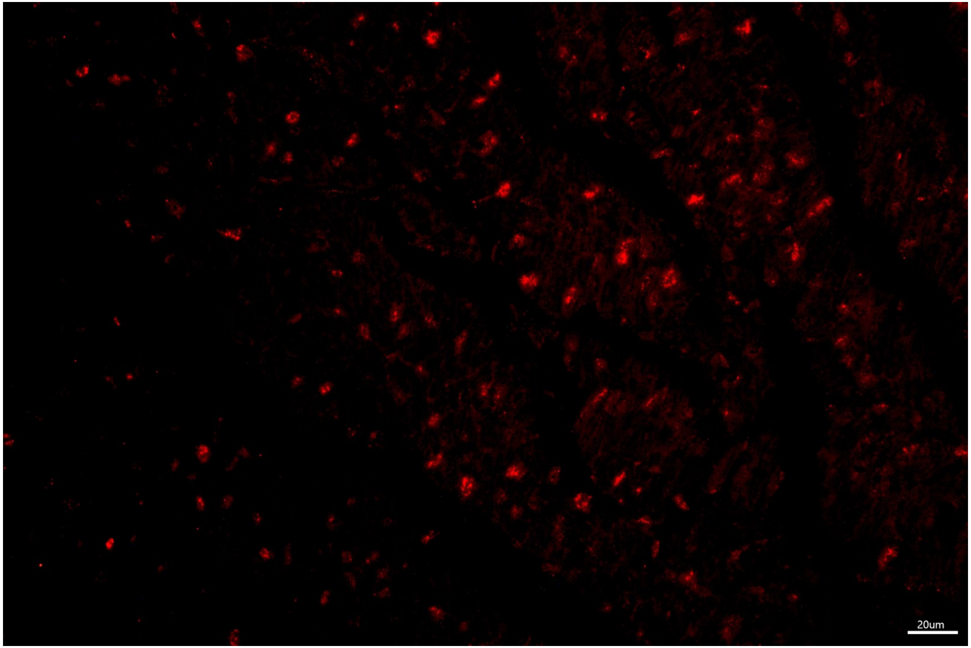

Original Image for Fig 10

(DCM+R, p-P38MAPK)

TNF-α

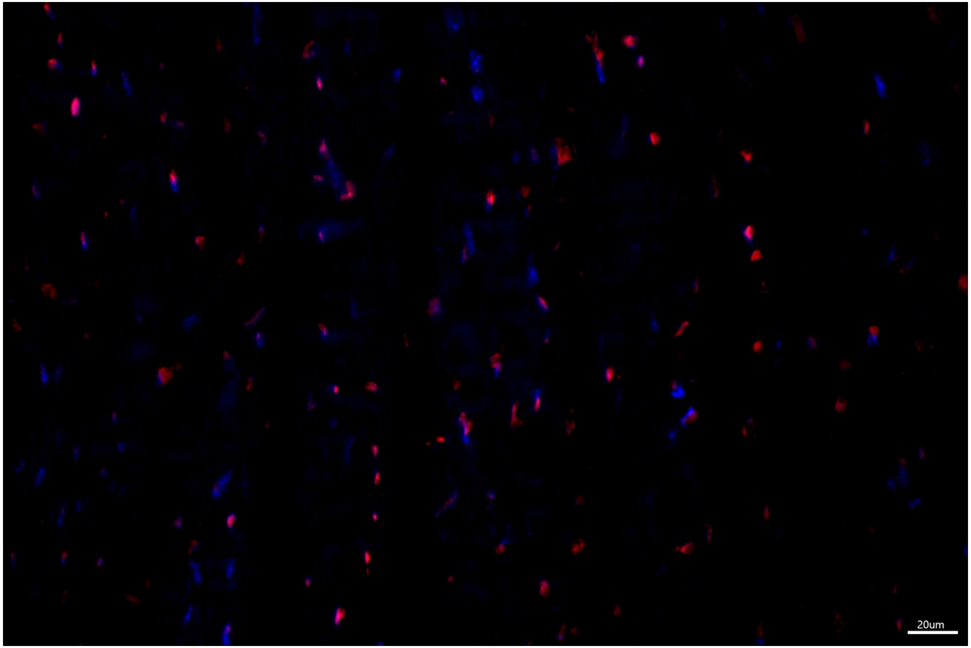

Original Image for Fig 10

(Con, Merge)

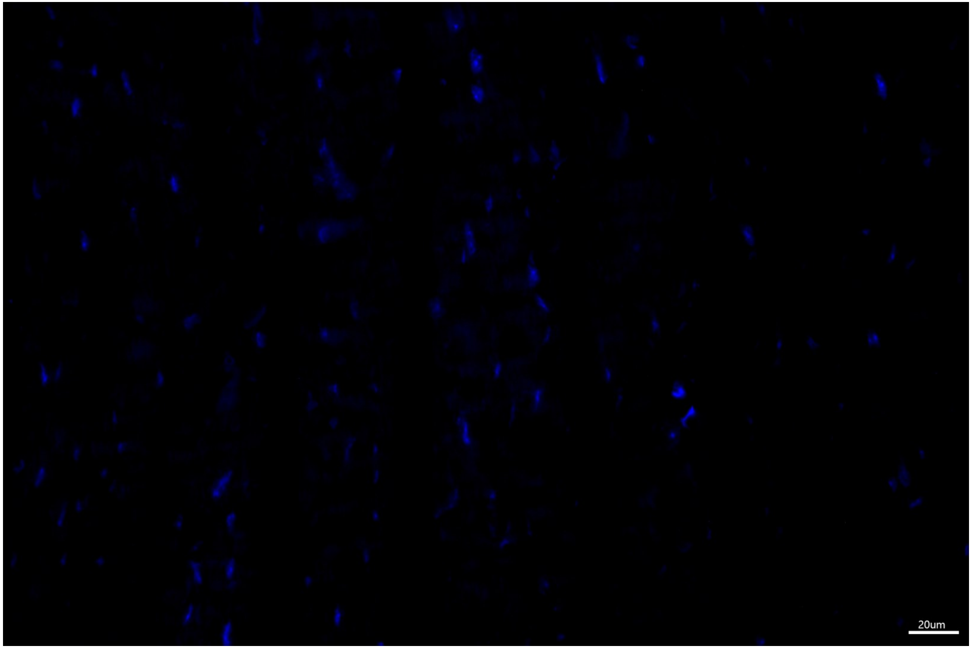

Original Image for Fig 10

(Con, DAPI)

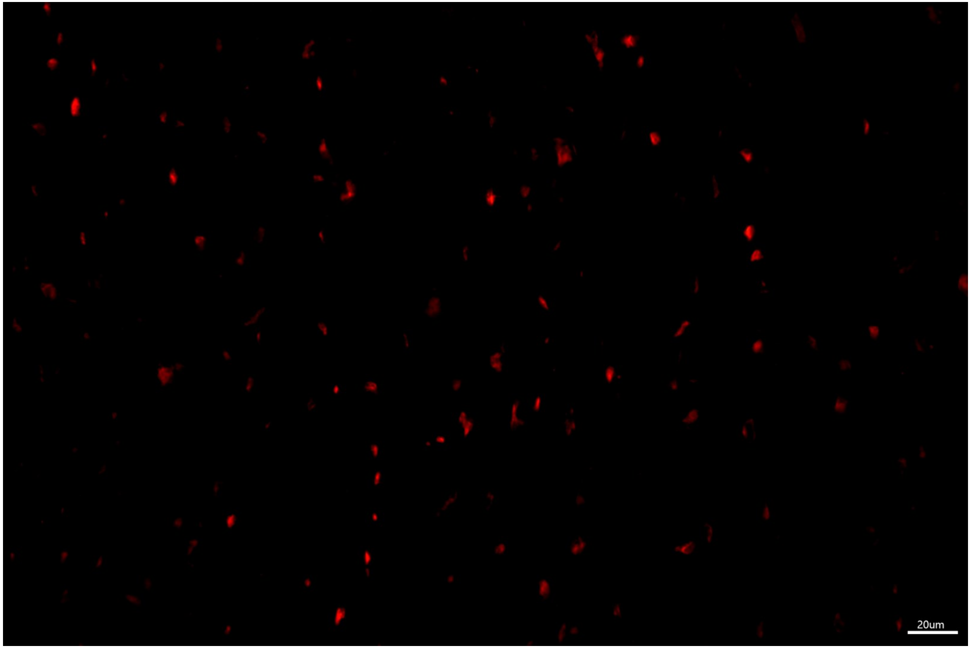

Original Image for Fig 10

(Con, TNF-α)

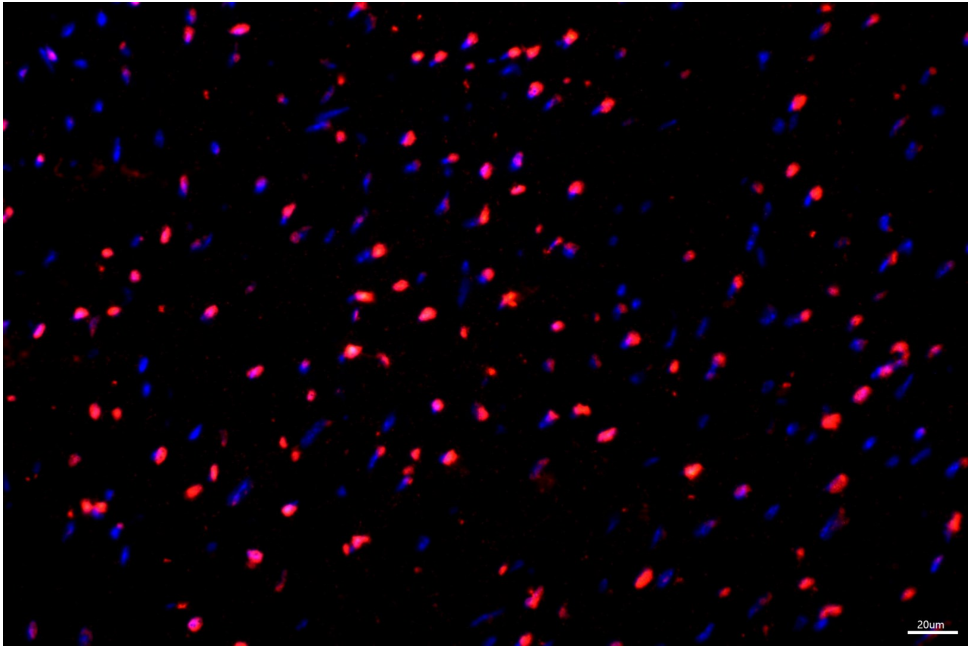

Original Image for Fig 10

(DCM, Merge)

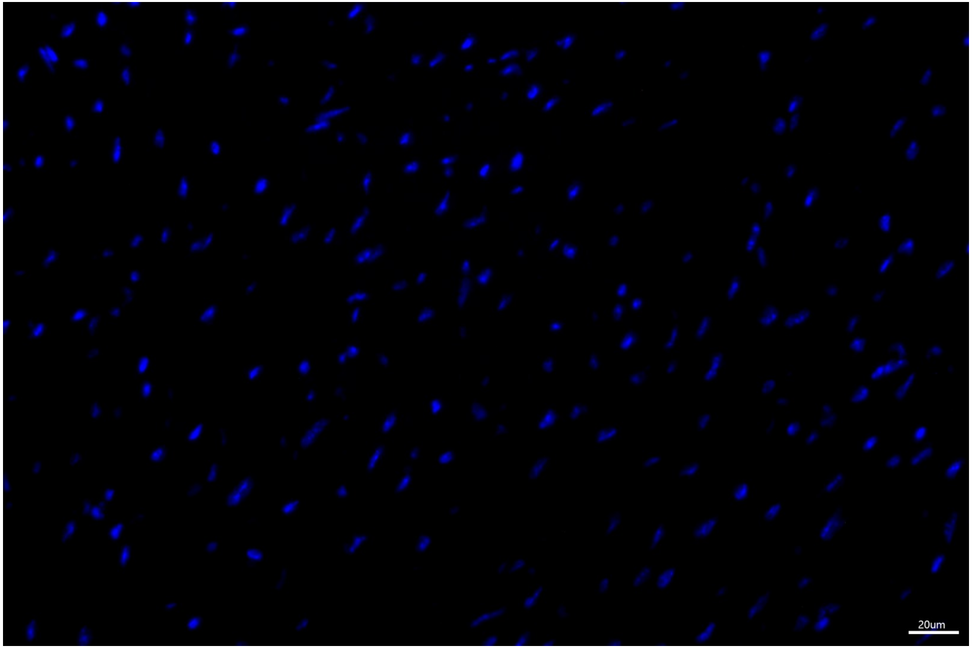

Original Image for Fig 10

(DCM, DAPI)

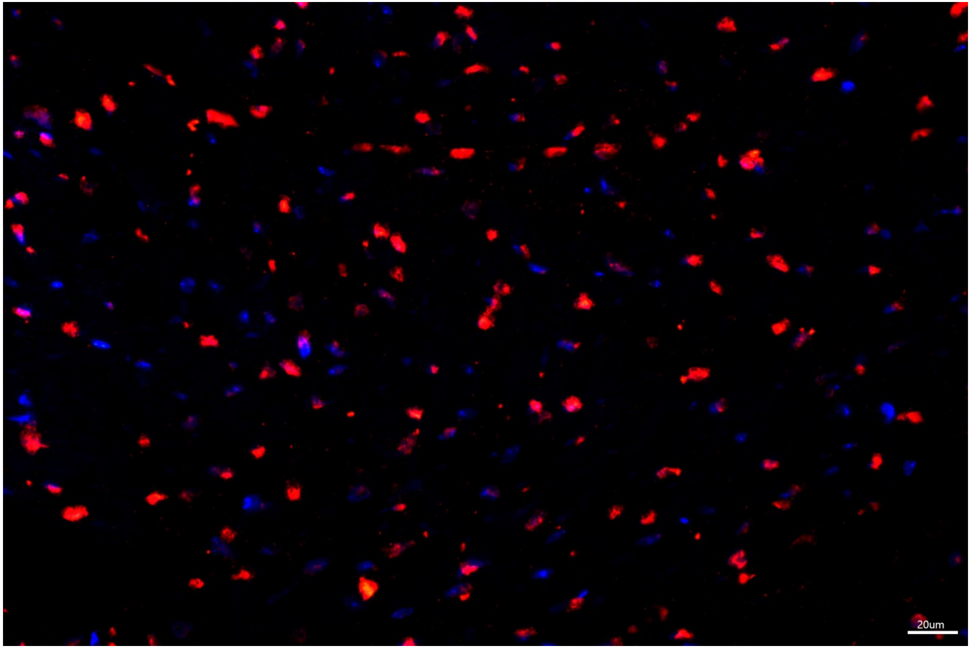

Original Image for Fig 10

(DCM, TNF-α)

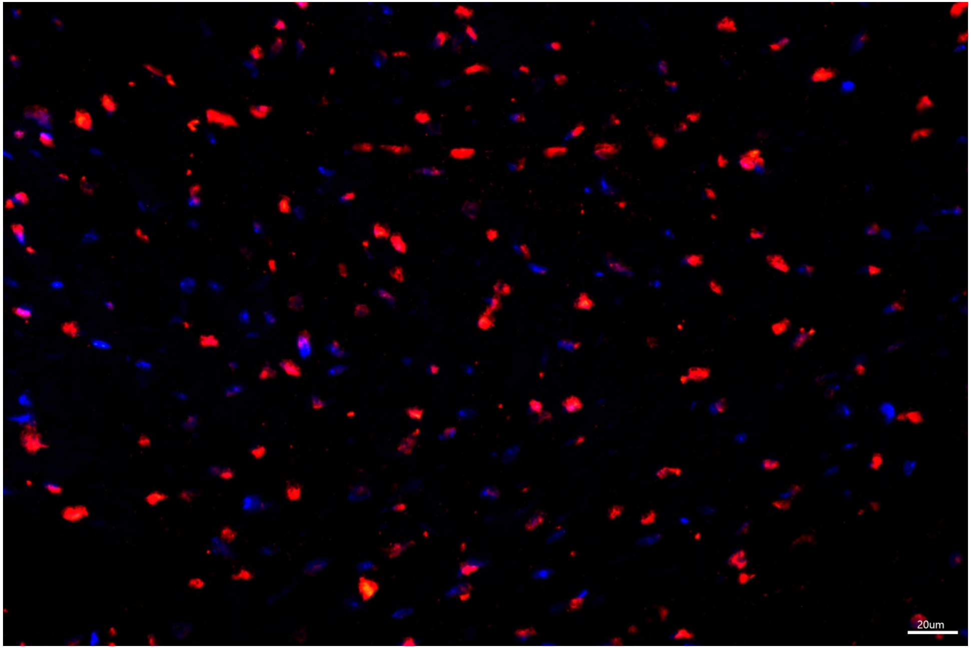

Original Image for Fig 10

(DCM+ZL, Merge)

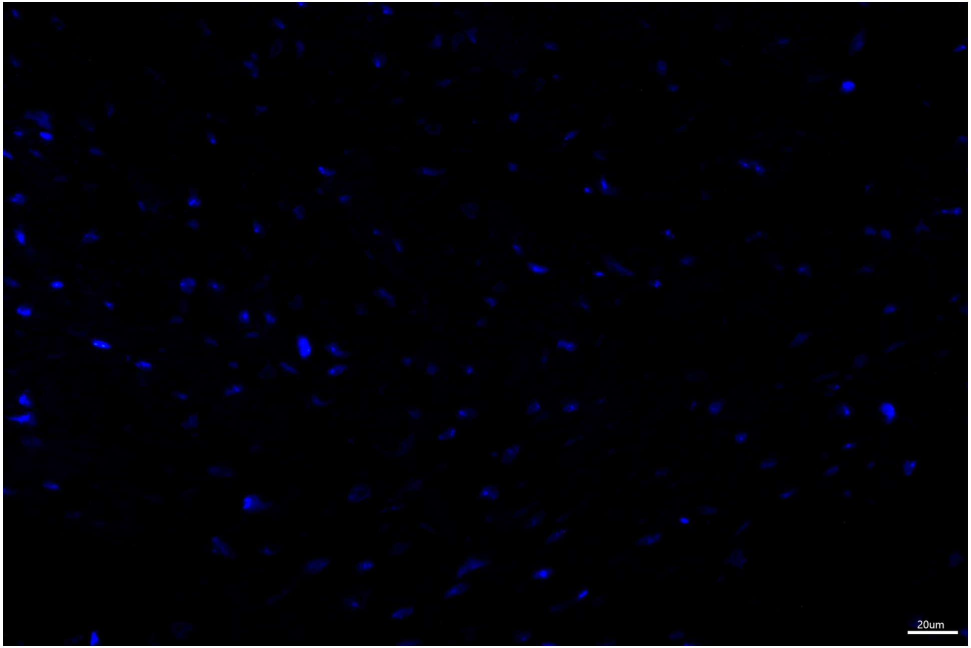

Original Image for Fig 10

(DCM+ZL, DAPI)

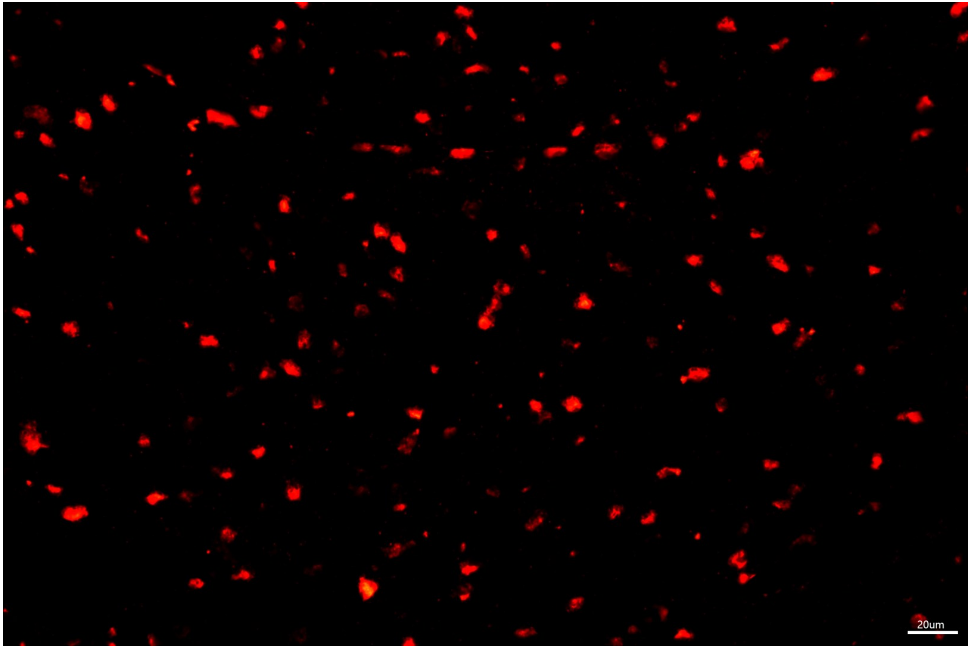

Original Image for Fig 10

(DCM+ZL, TNF-α)

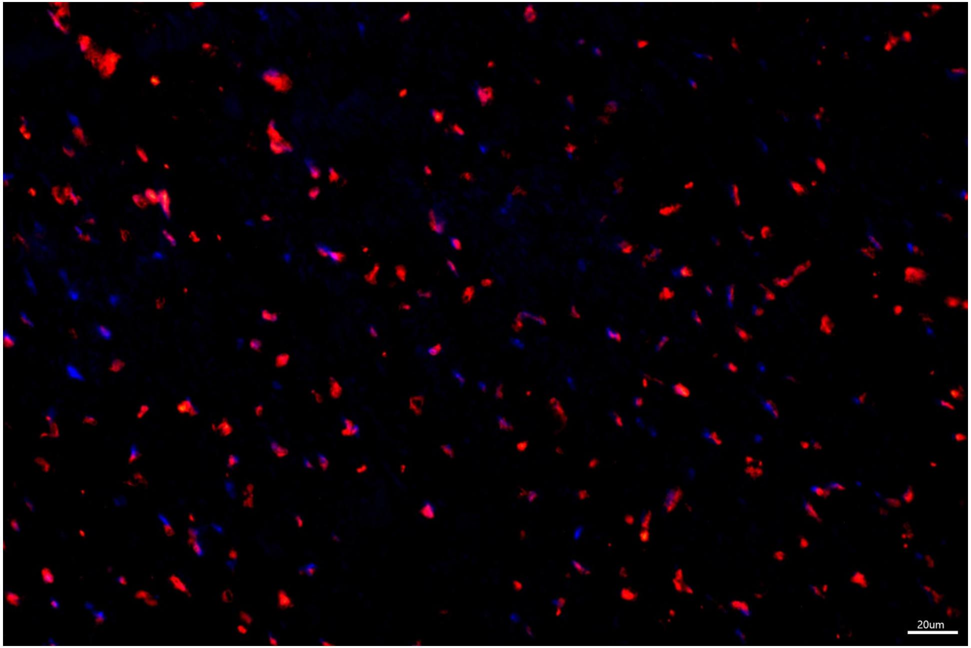

Original Image for Fig 10

(SB203580, Merge)

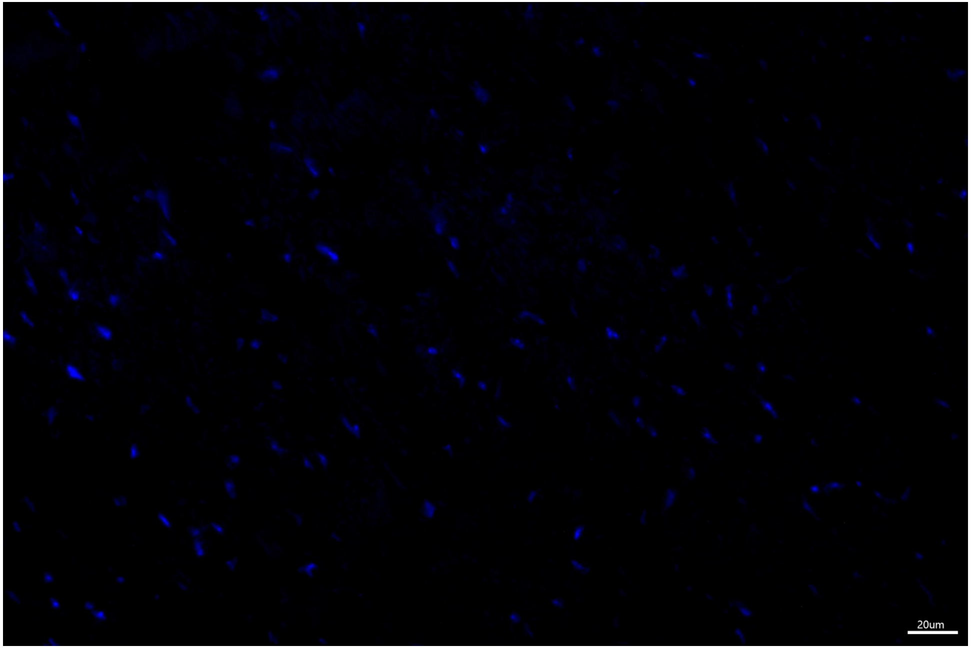

Original Image for Fig 10

(SB203580, DAPI)

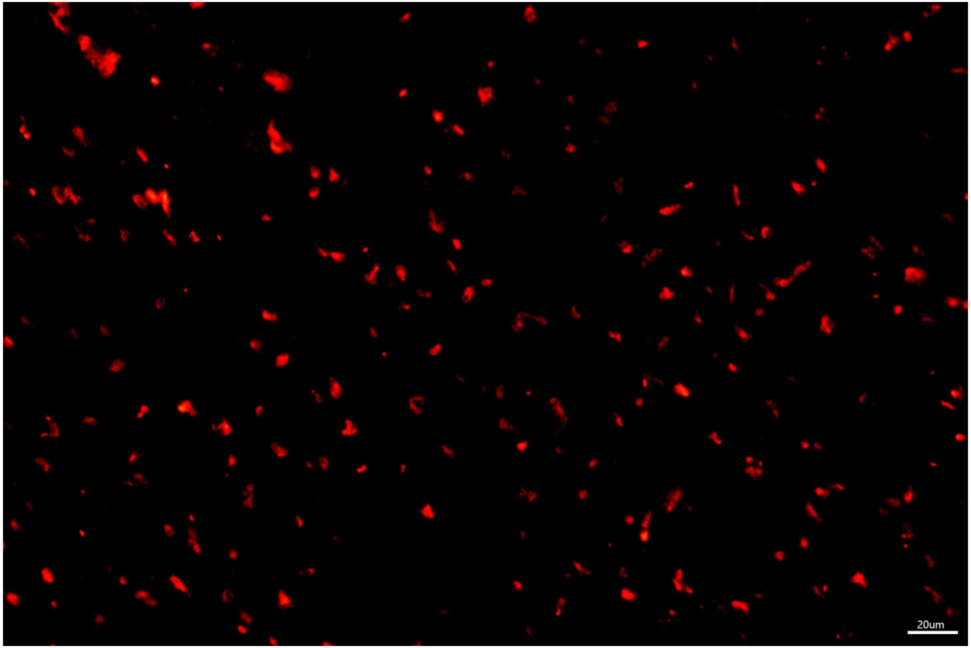

Original Image for Fig 10

(SB203580, TNF-α)

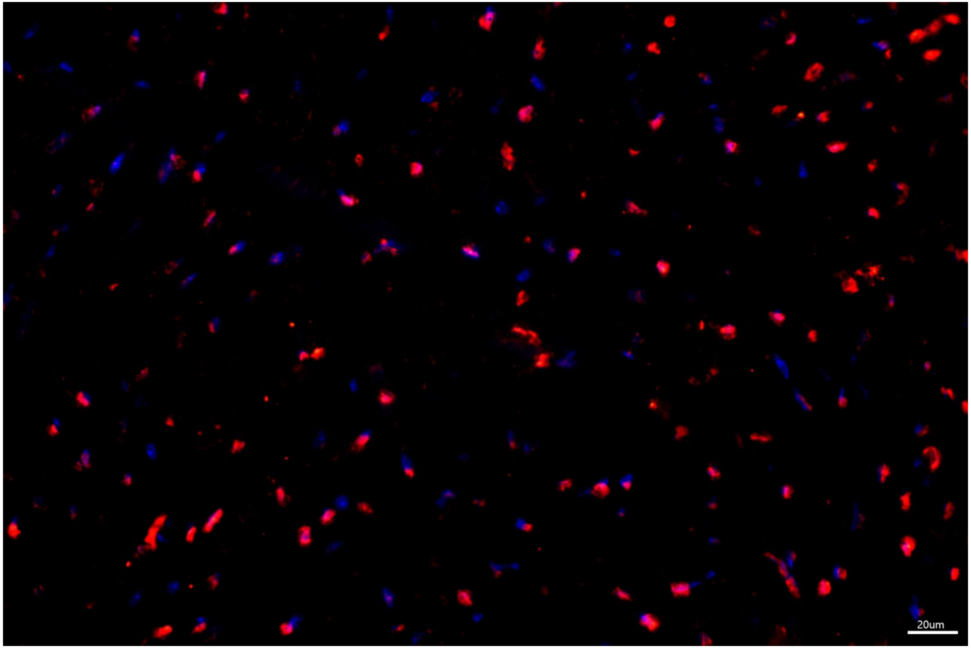

Original Image for Fig 10

(DCM+R, Merge)

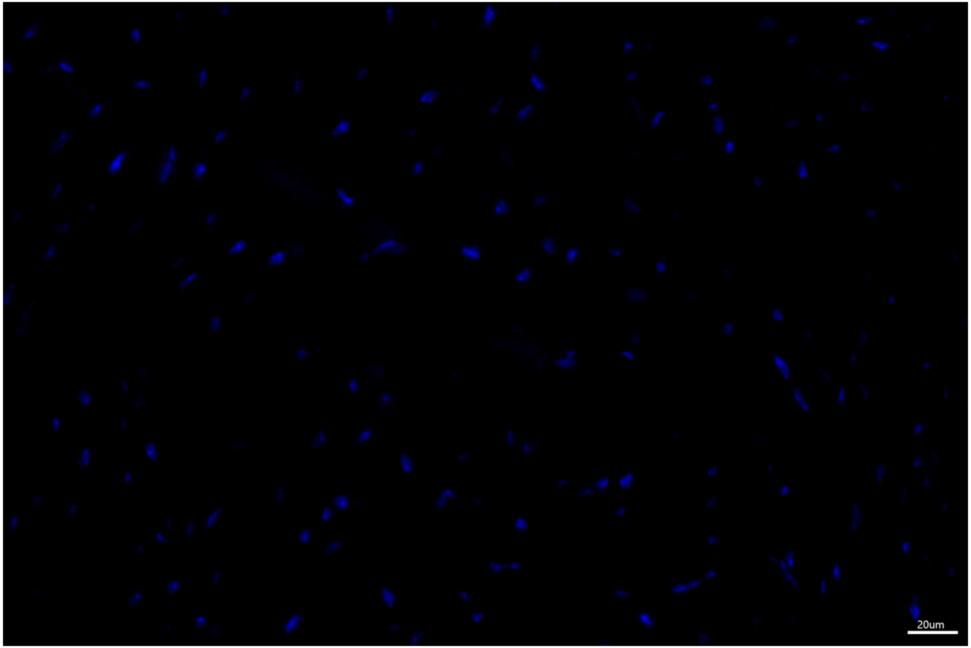

Original Image for Fig 10

(DCM+R, DAPI)

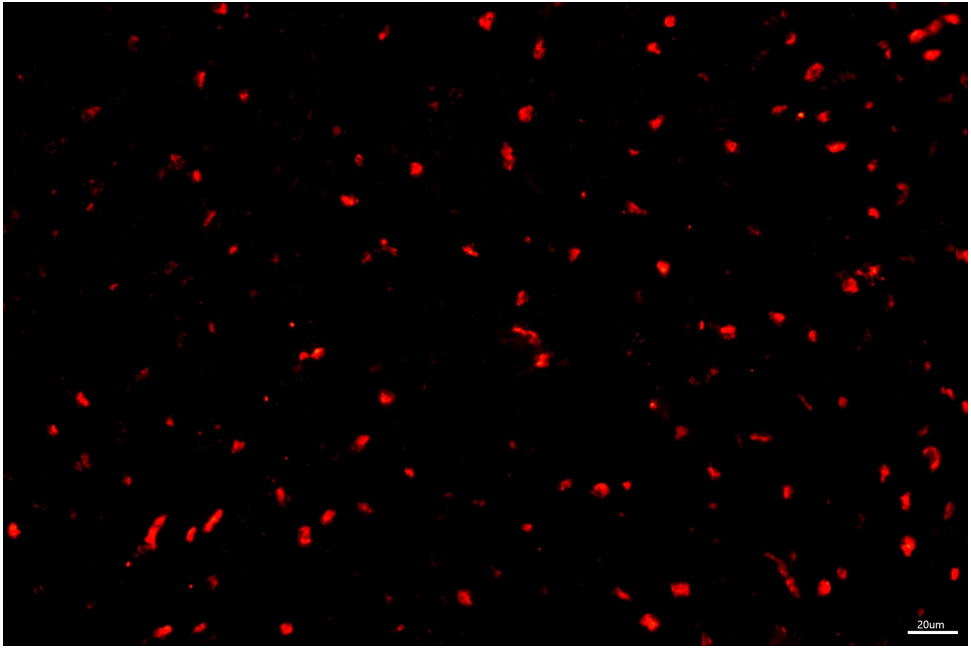

Original Image for Fig 10

(DCM+R,TNF-α)

α-SMA

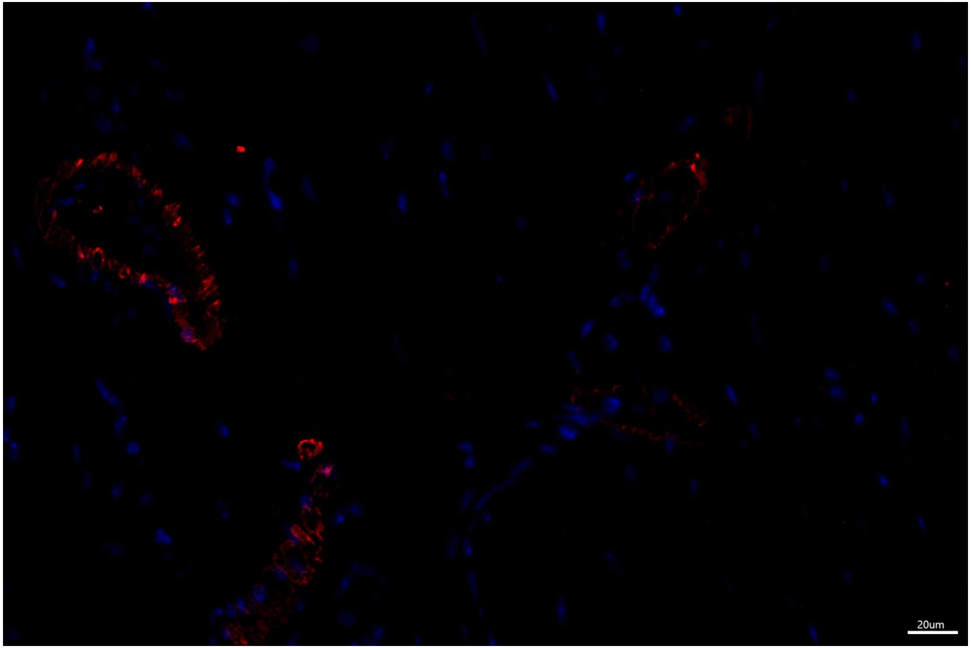

Original Image for Fig 10

(Con, Merge)

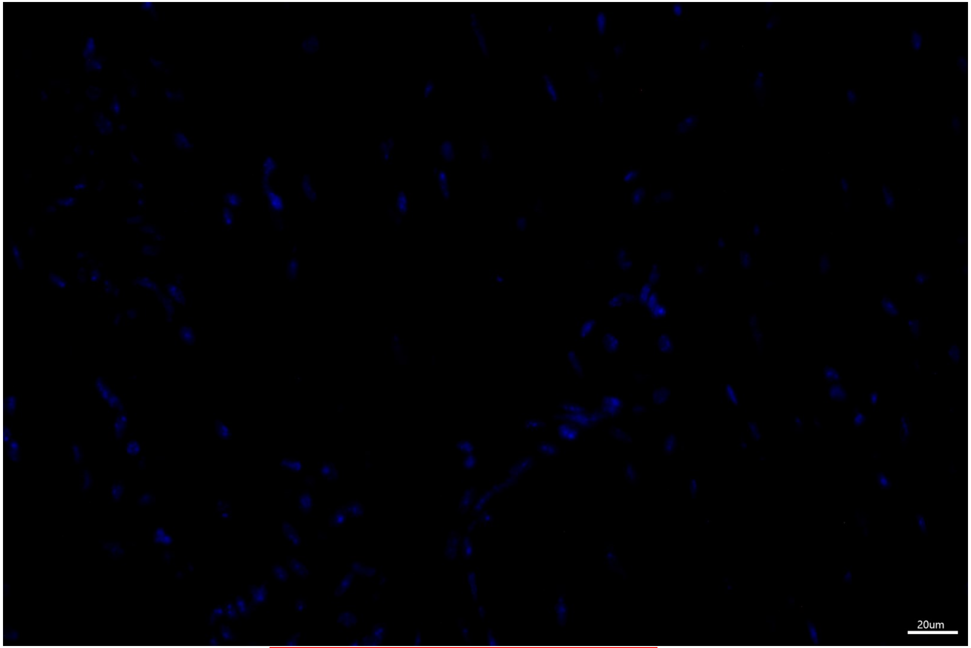

Original Image for Fig 10
(Con, DAPI)

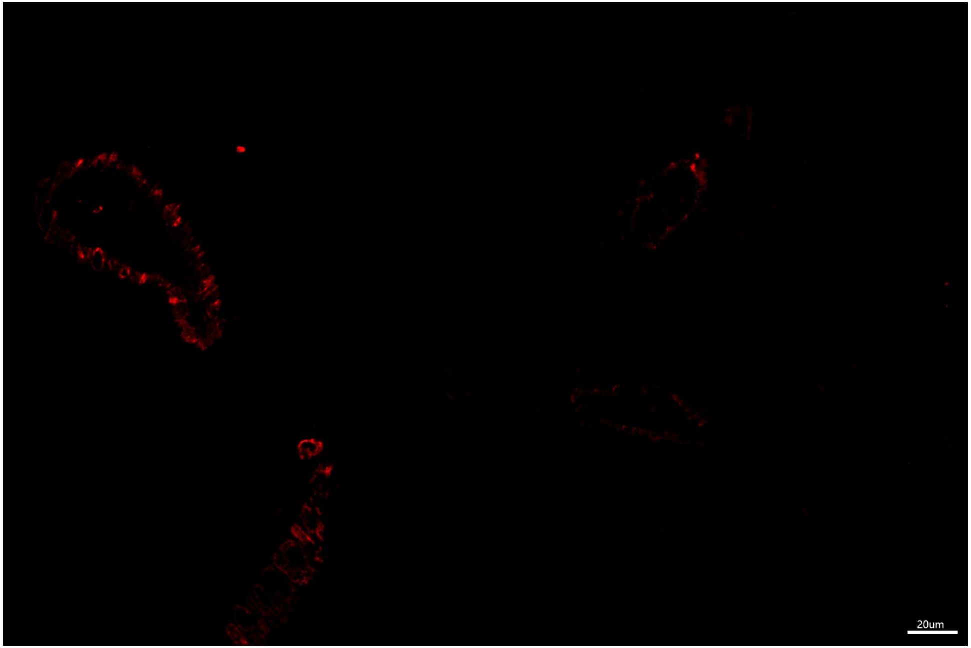

Original Image for Fig 10

(Con, α-SMA)

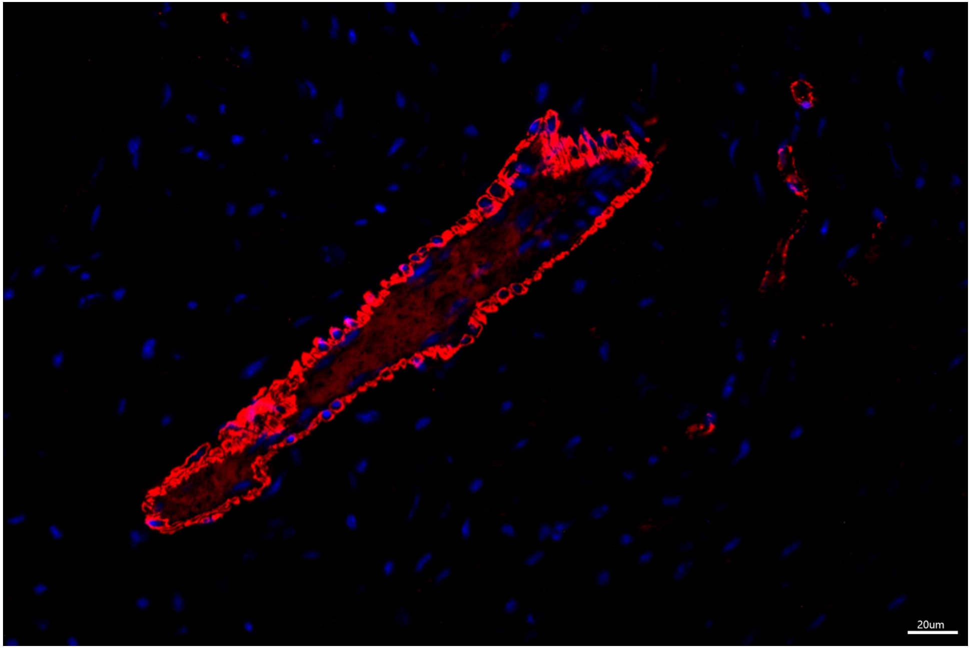

Original Image for Fig 10

(DCM, Merge)

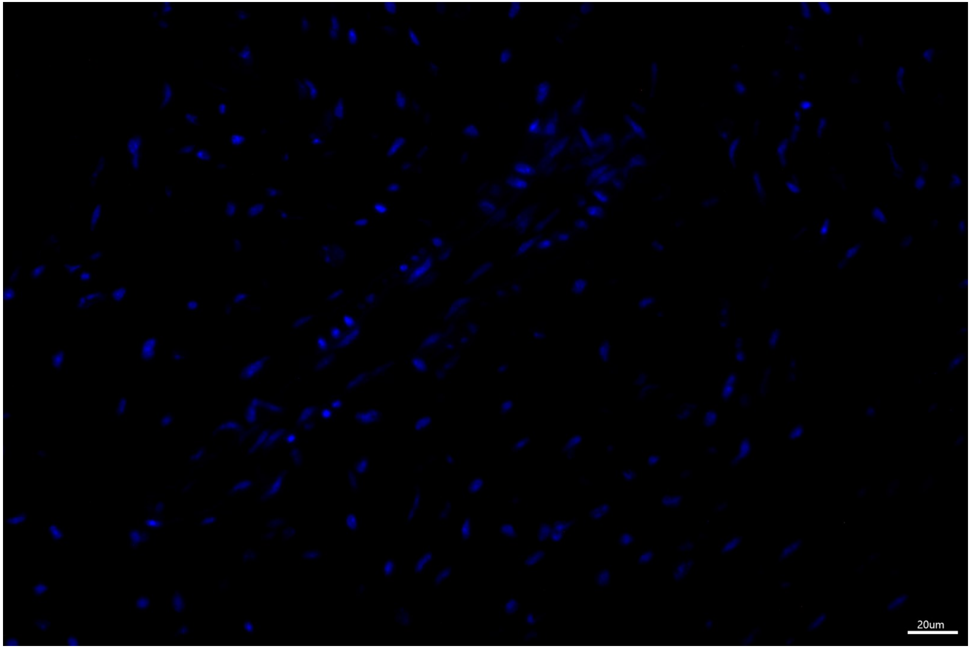

Original Image for Fig 10

(DCM, DAPI)

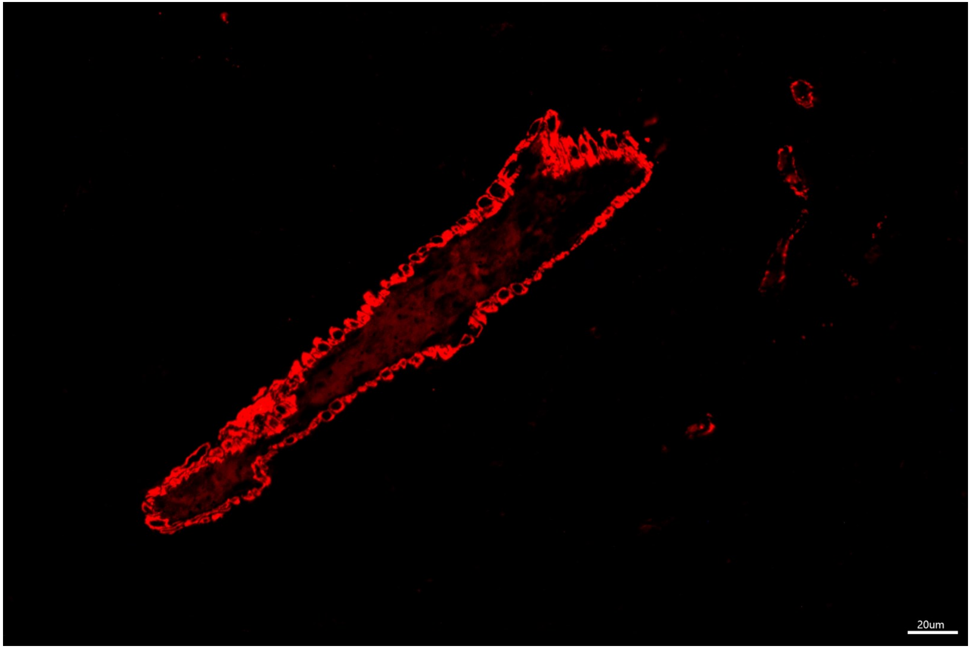

Original Image for Fig 10
(DCM, α-SMA)

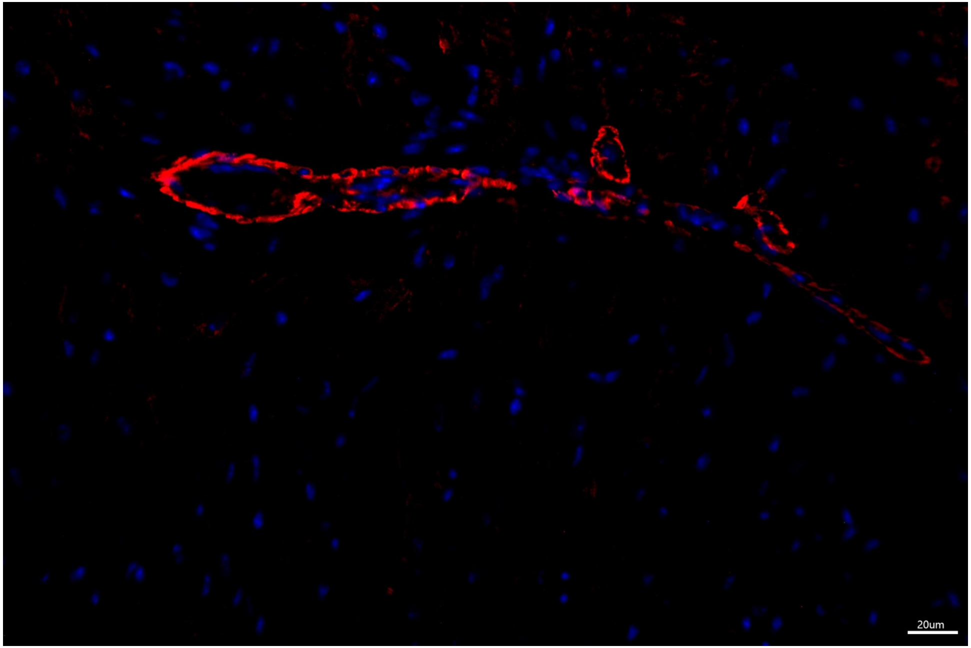

Original Image for Fig 10

(DCM+ZL, Merge)

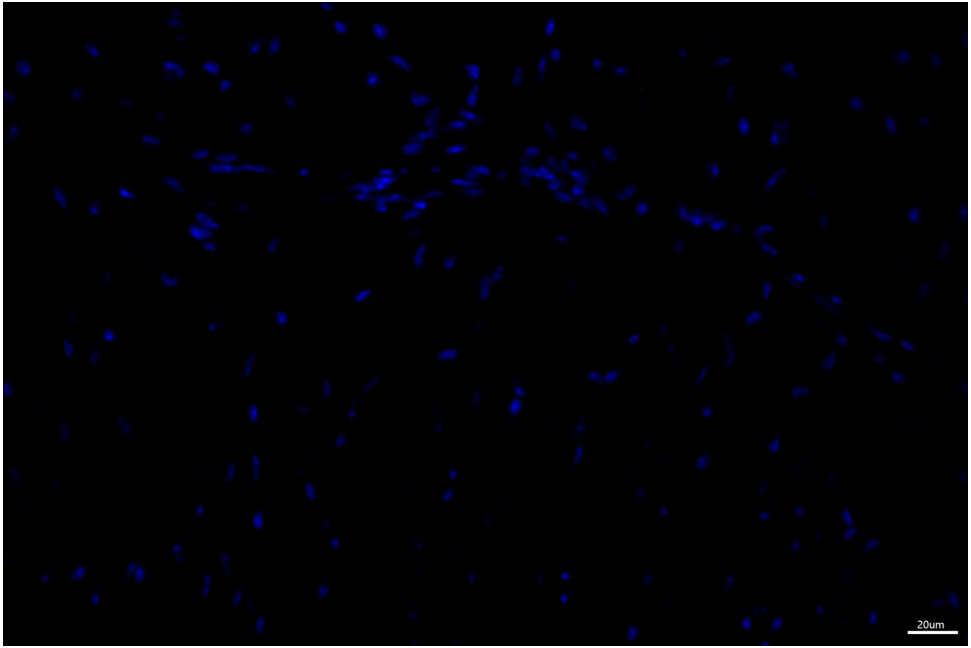

Original Image for Fig 10

(DCM+ZL, DAPI)

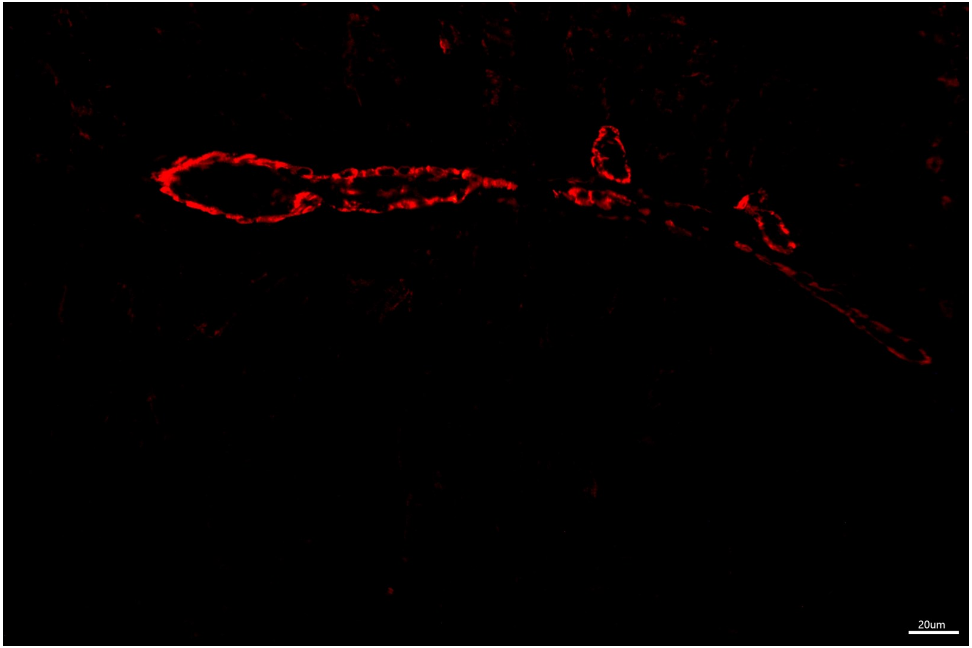

Original Image for Fig 10

(DCM+ZL, α-SMA)

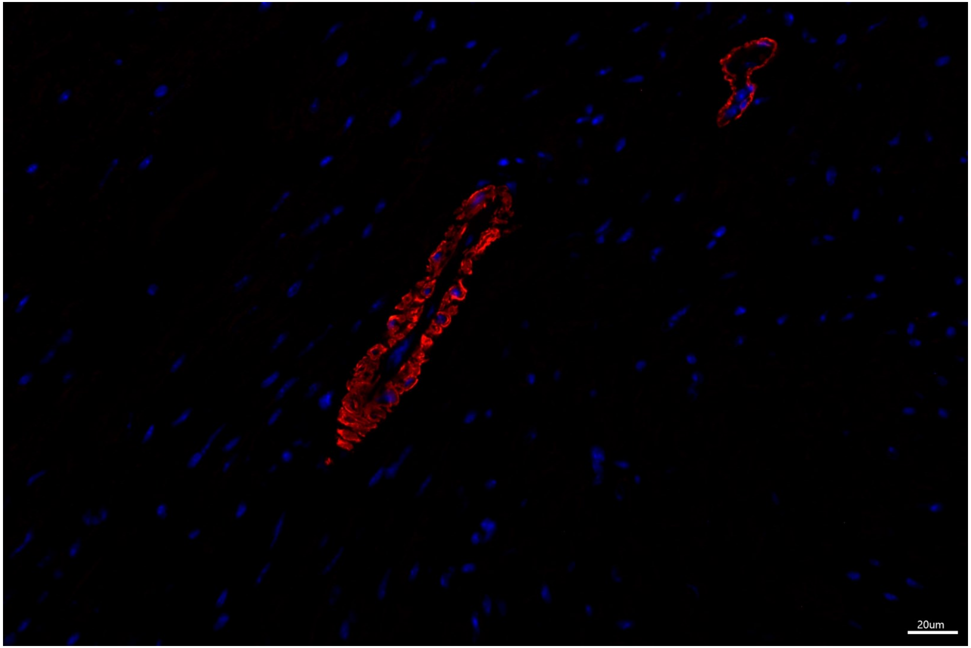

Original Image for Fig 10
(SB203580, Merge)

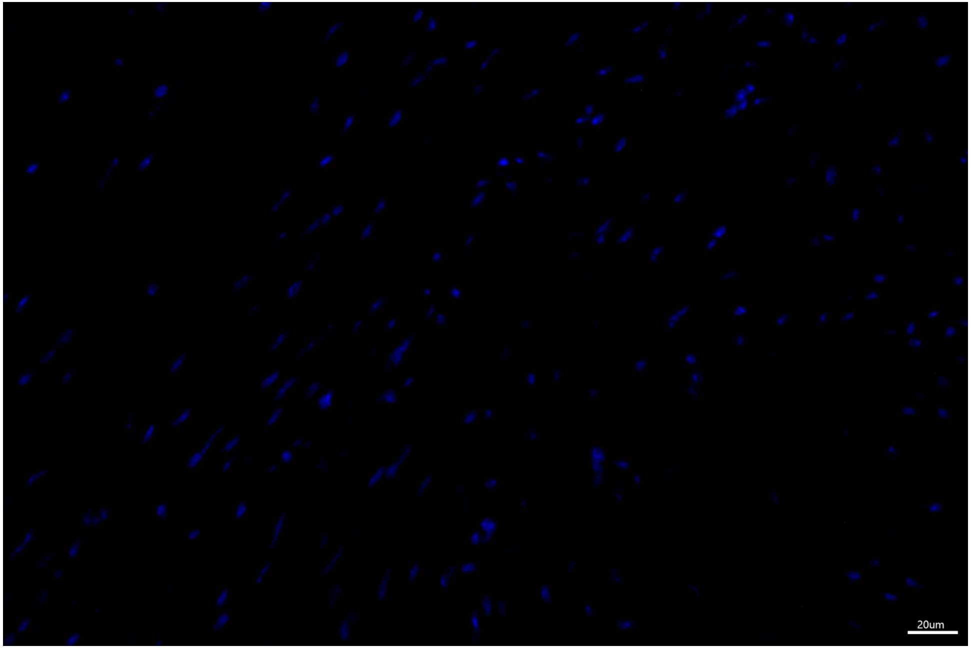

Original Image for Fig 10

(SB203580, DAPI)

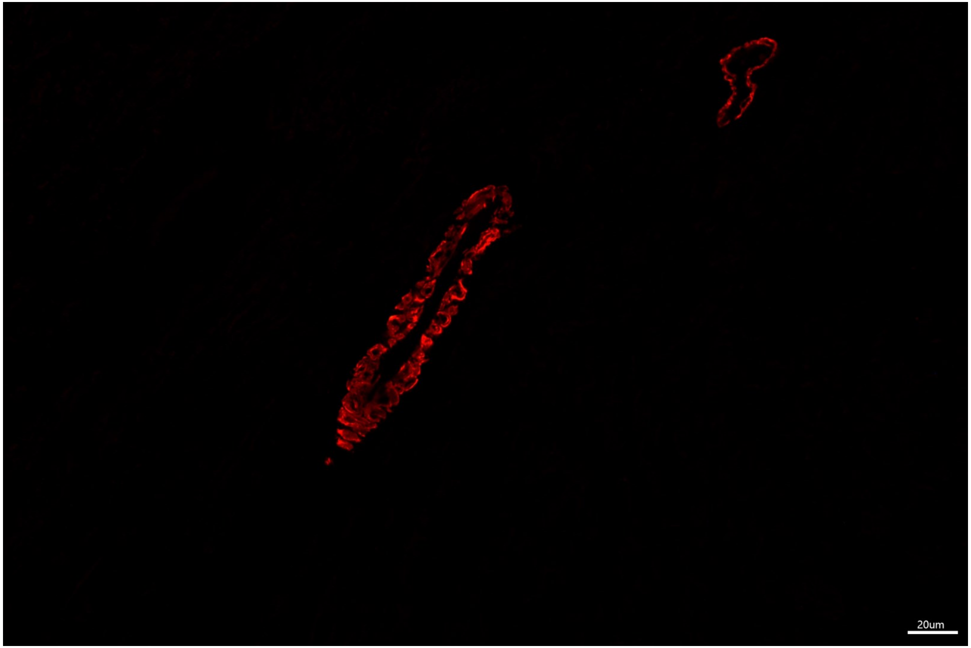

Original Image for Fig 10

(SB203580, α-SMA)

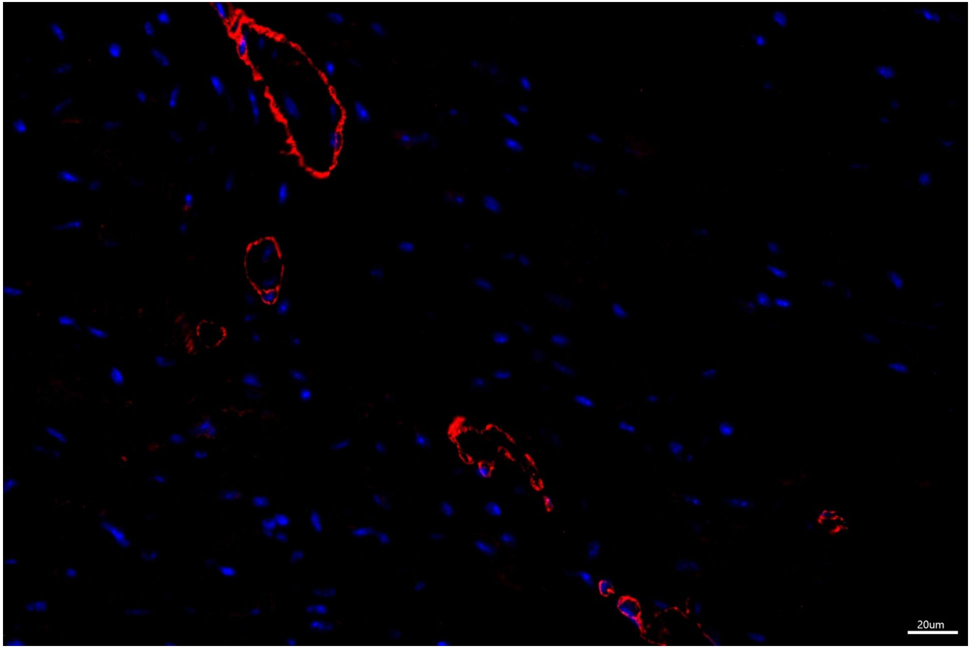

Original Image for Fig 10

(DCM+R, Merge)

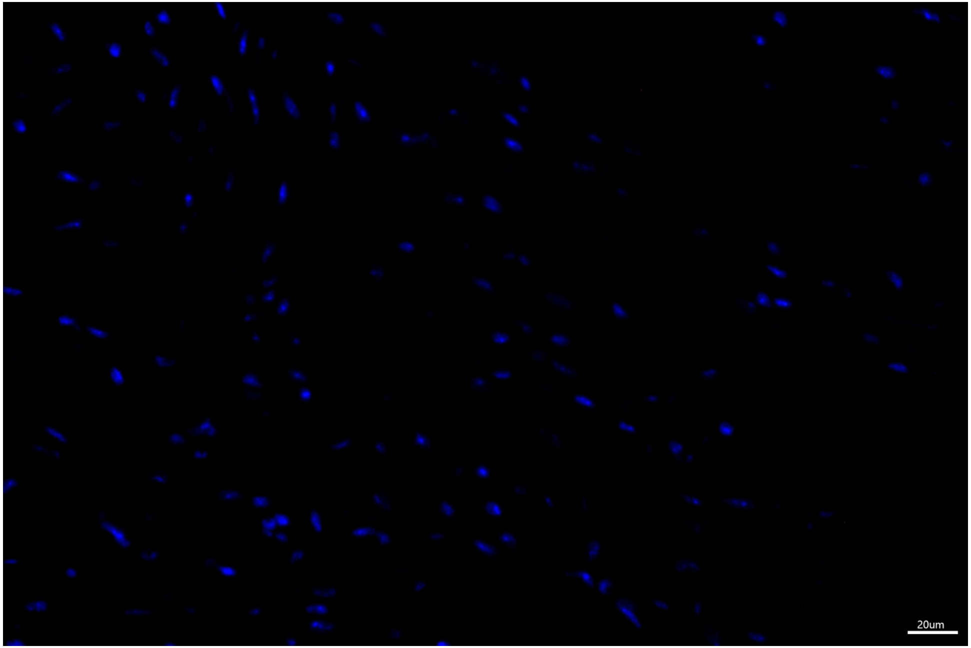

Original Image for Fig 10

(DCM+R, DAPI)

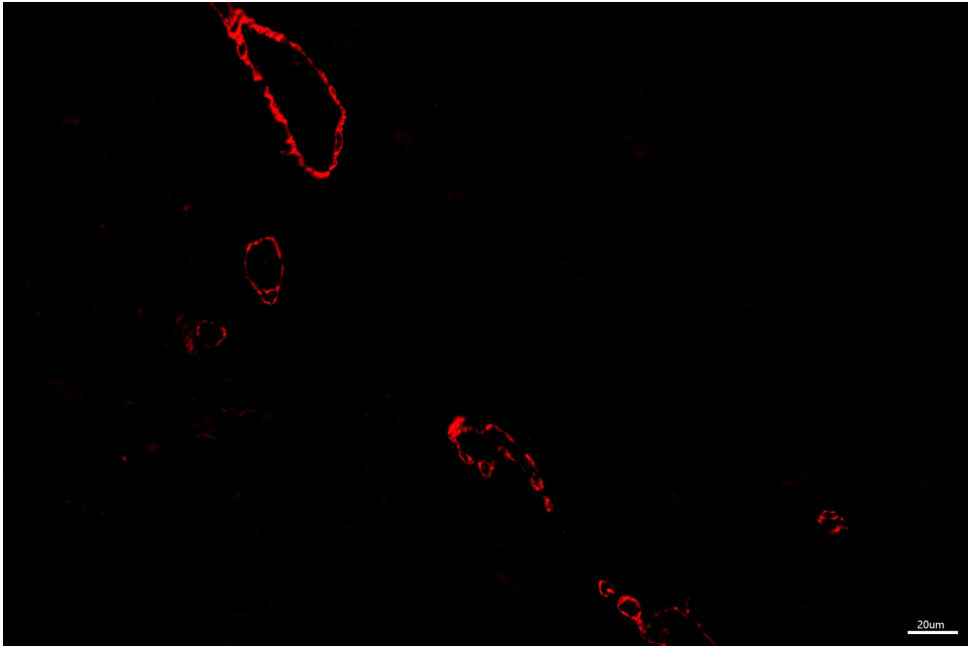

Original Image for Fig 10

(DCM+R, α-SMA)

Collagen-I

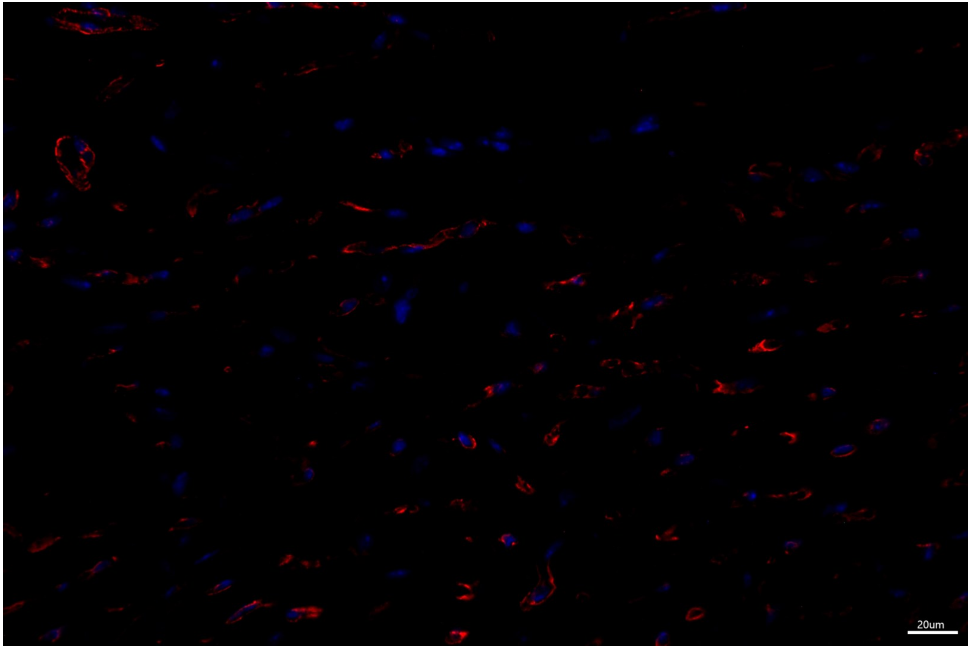

Original Image for Fig 10

(Con, Merge)

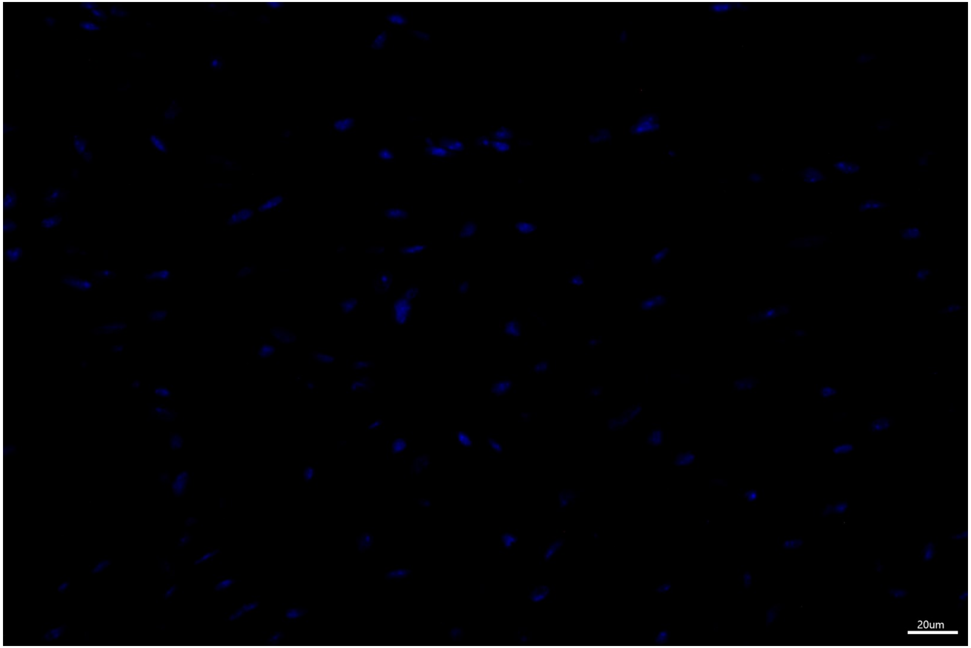

Original Image for Fig 10

(Con, DAPI)

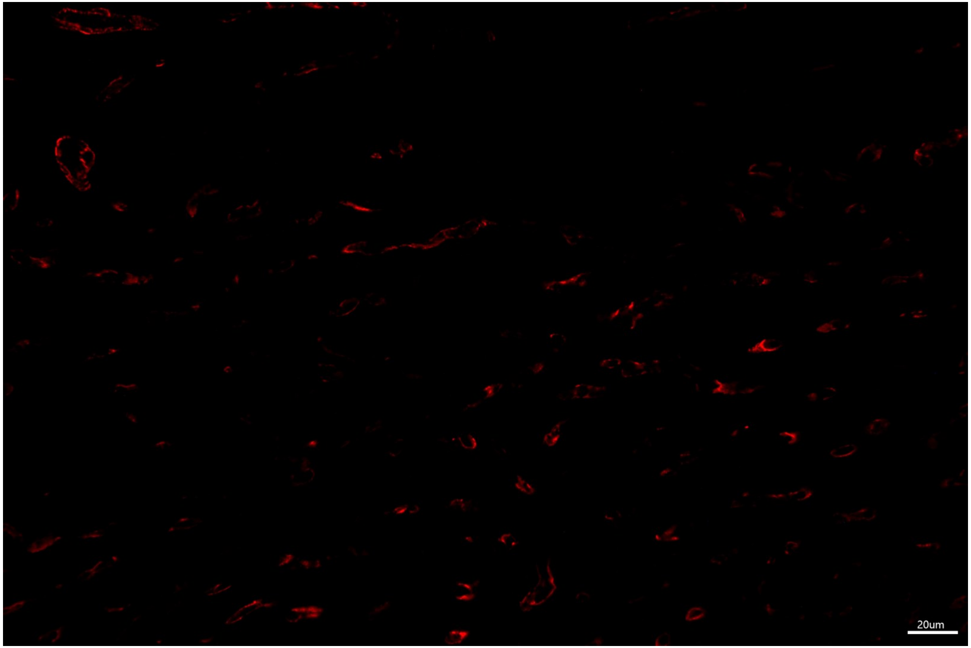

Original Image for Fig 10

(Con, Collagen-I)

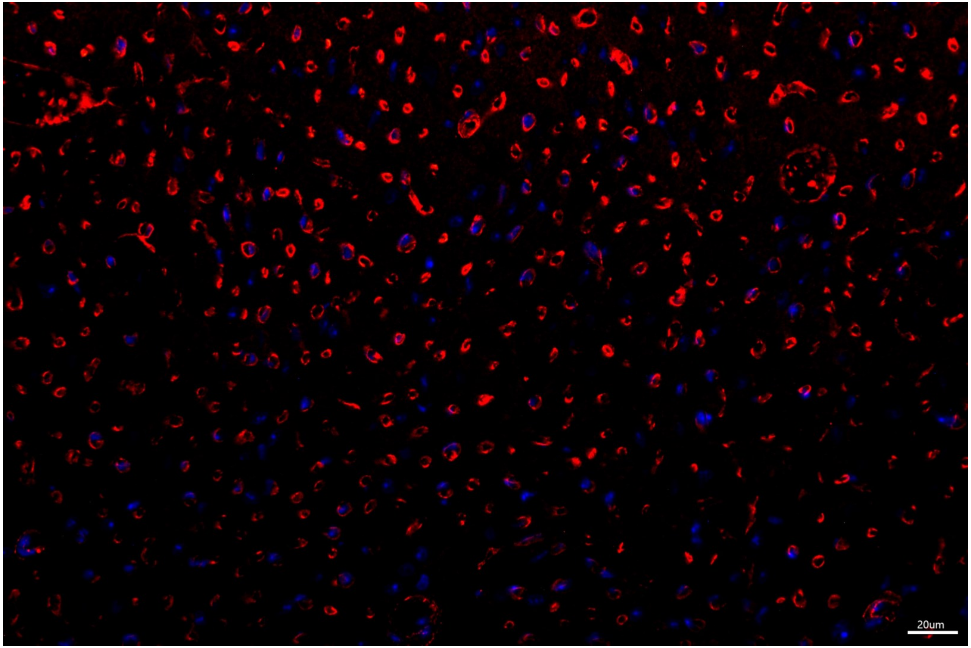

Original Image for Fig 10

(DCM, Merge)

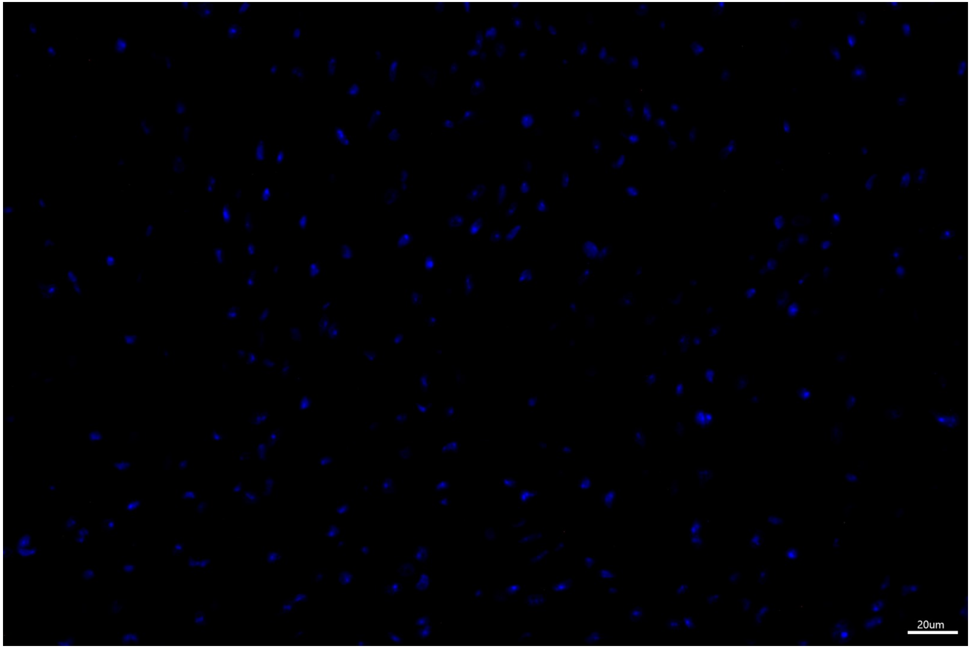

Original Image for Fig 10

(DCM, DAPI)

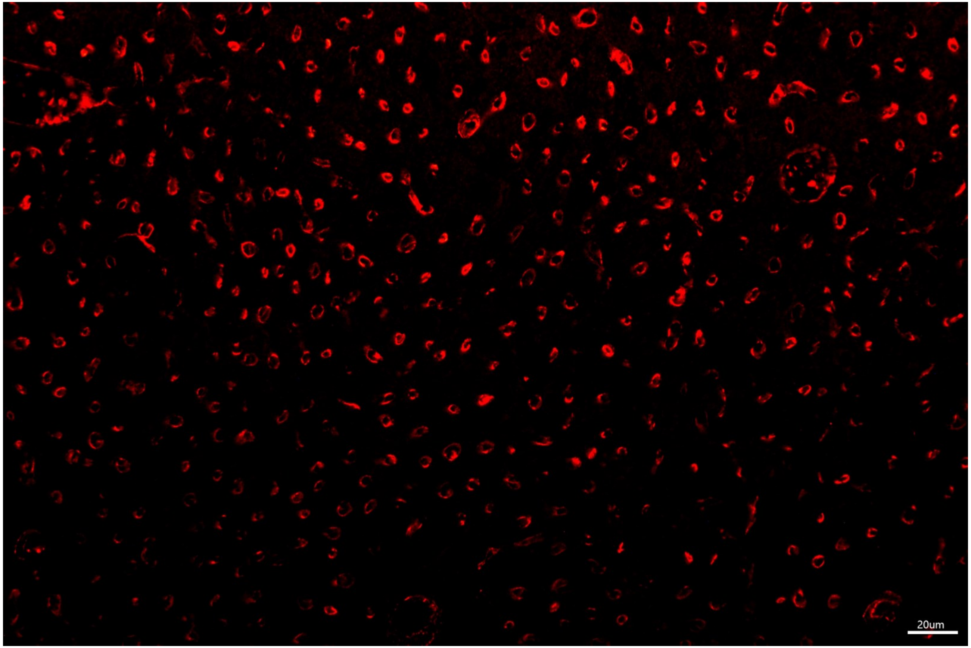

Original Image for Fig 10

(DCM, Collagen-I)

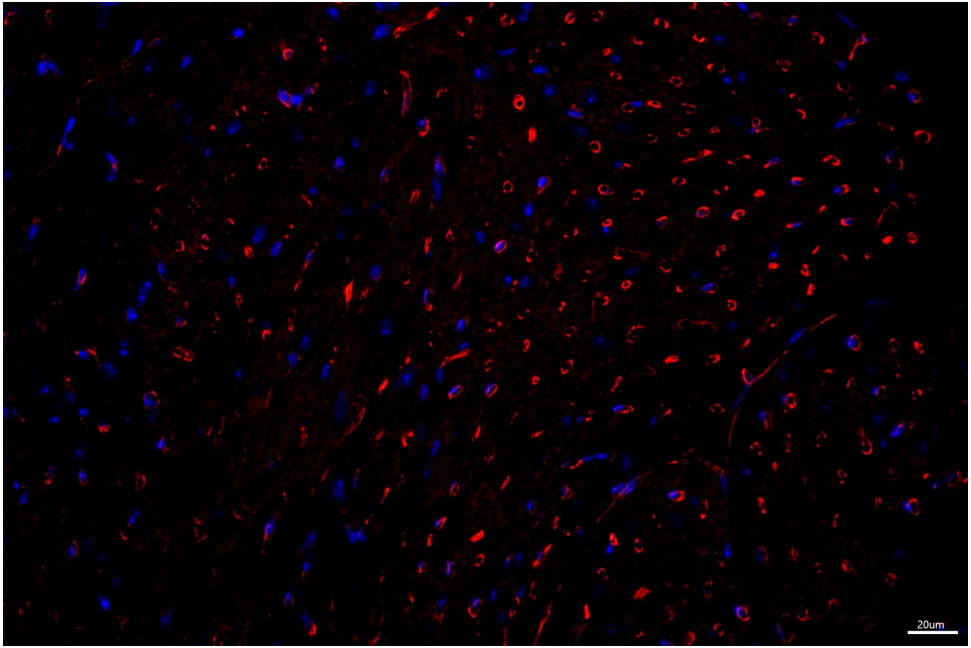

Original Image for Fig 10

(DCM+ZL, Merge)

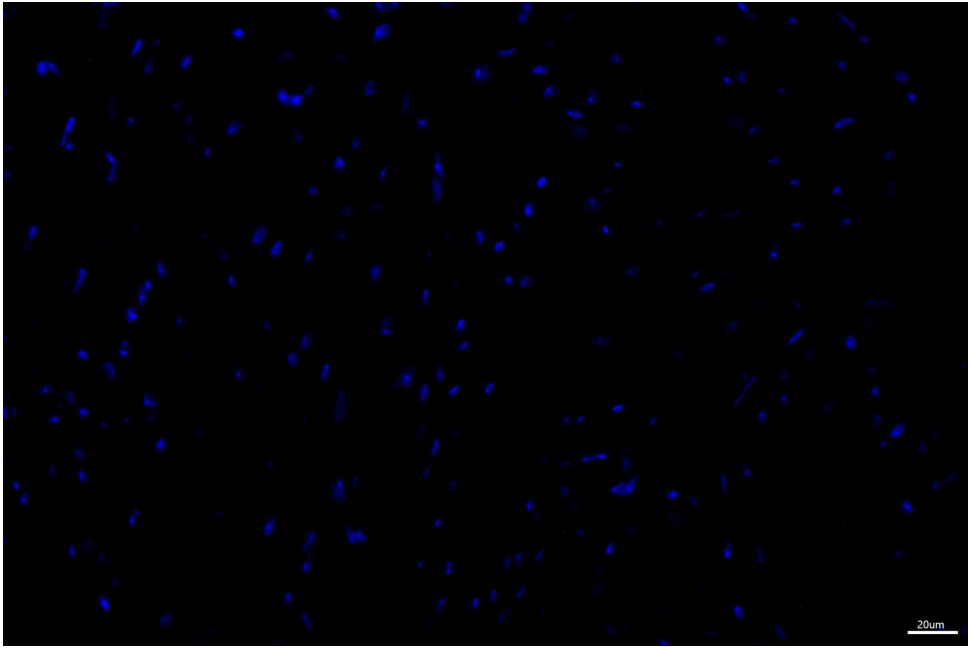

Original Image for Fig 10

(DCM+ZL, DAPI)

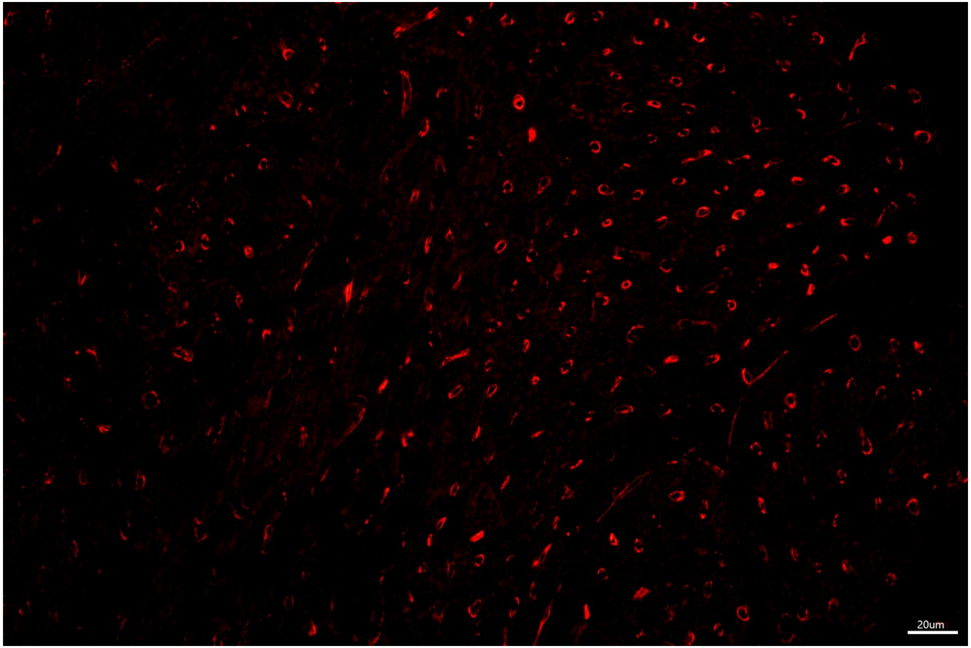

Original Image for Fig 10

(DCM+ZL, Collagen-I)

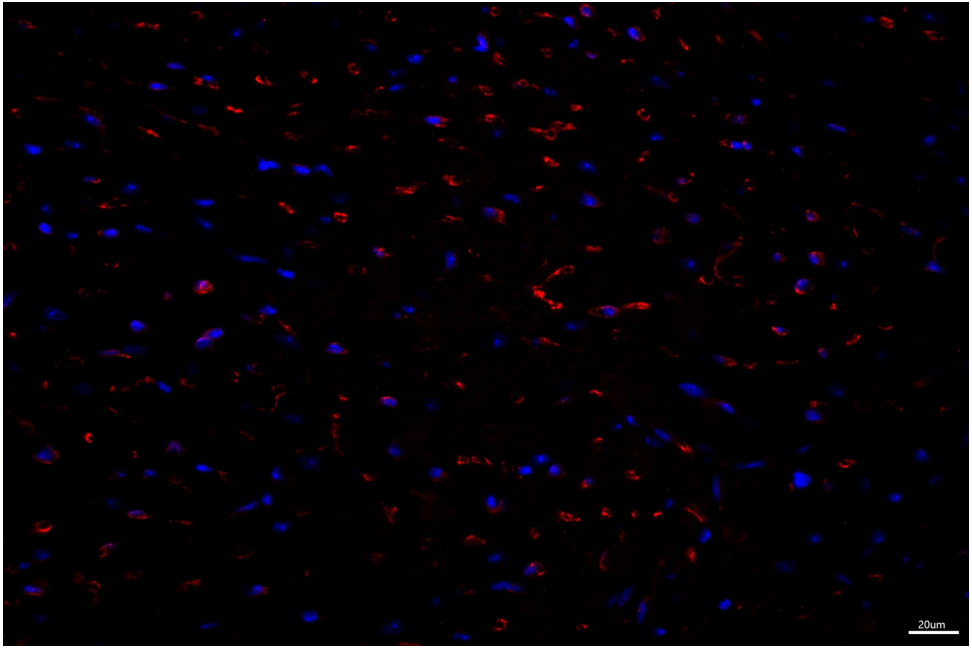

Original Image for Fig 10

(SB203580, Merge)

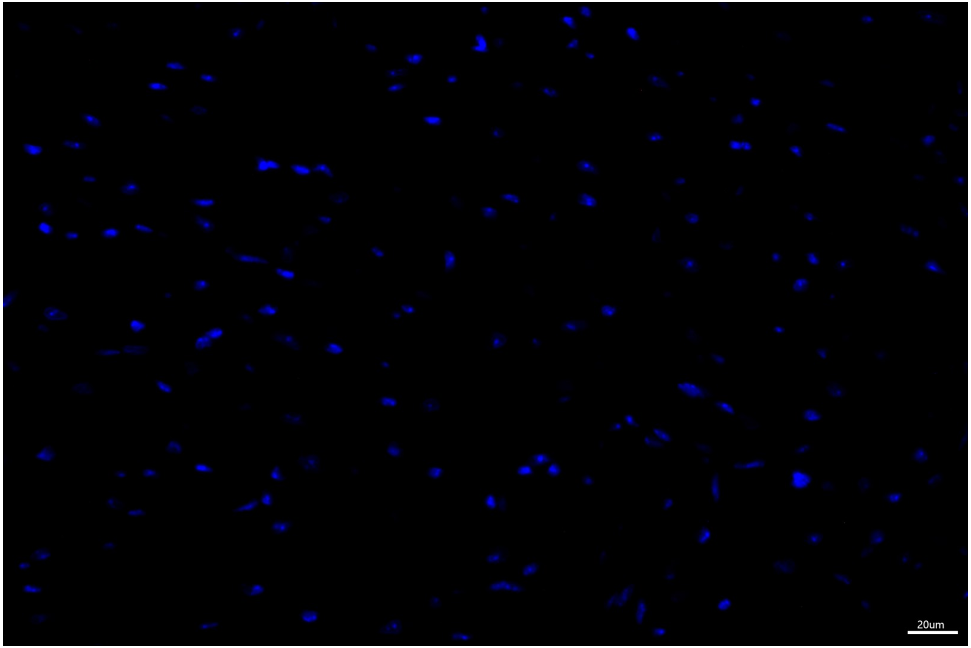

Original Image for Fig 10

(SB203580, DAPI)

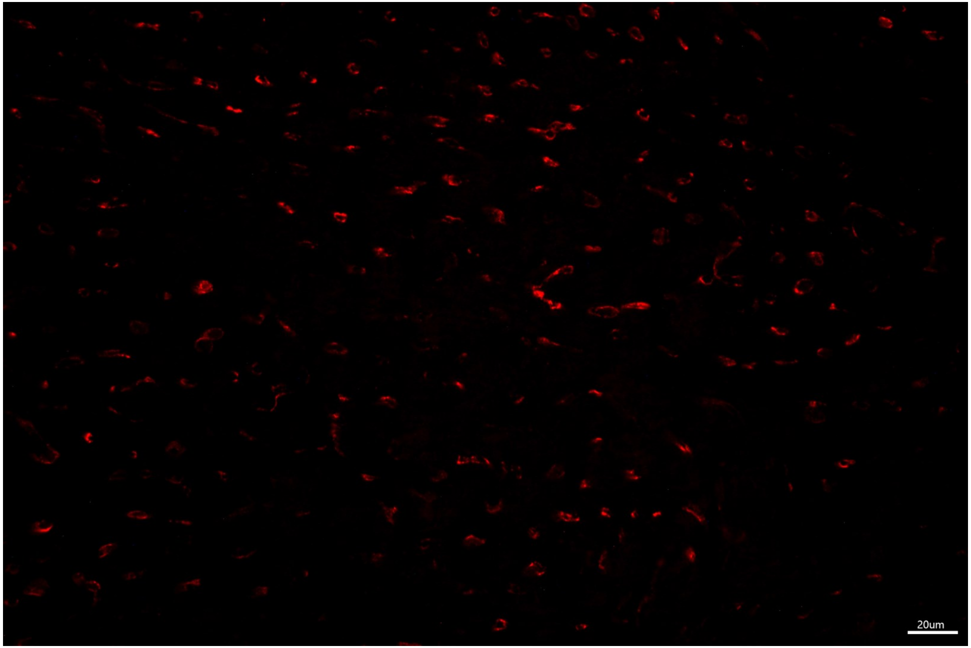

Original Image for Fig 10

(SB203580, Collagen-I)

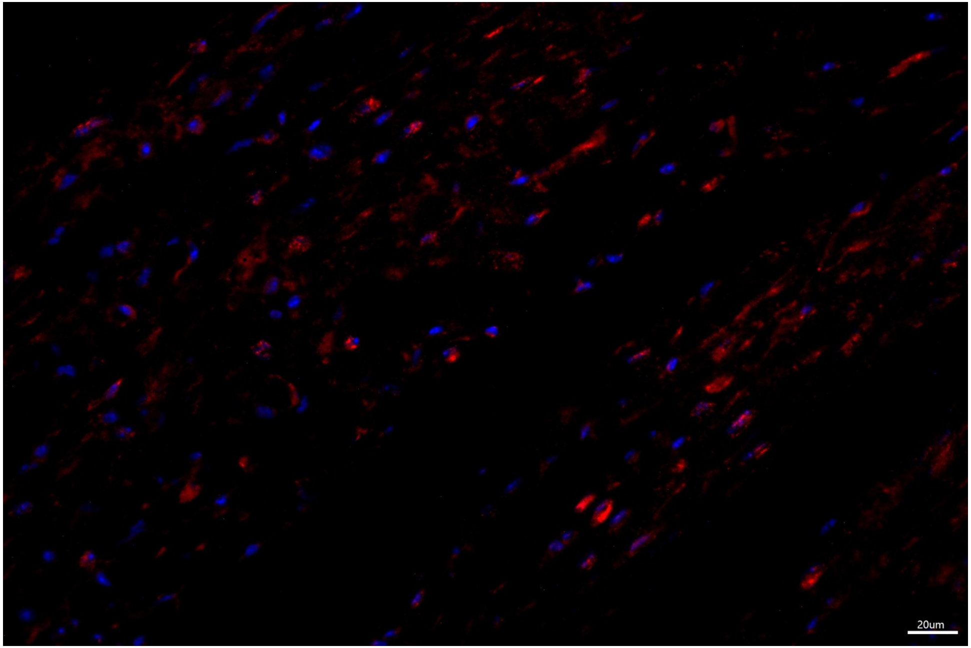

Original Image for Fig 10

(DCM+R, Merge)

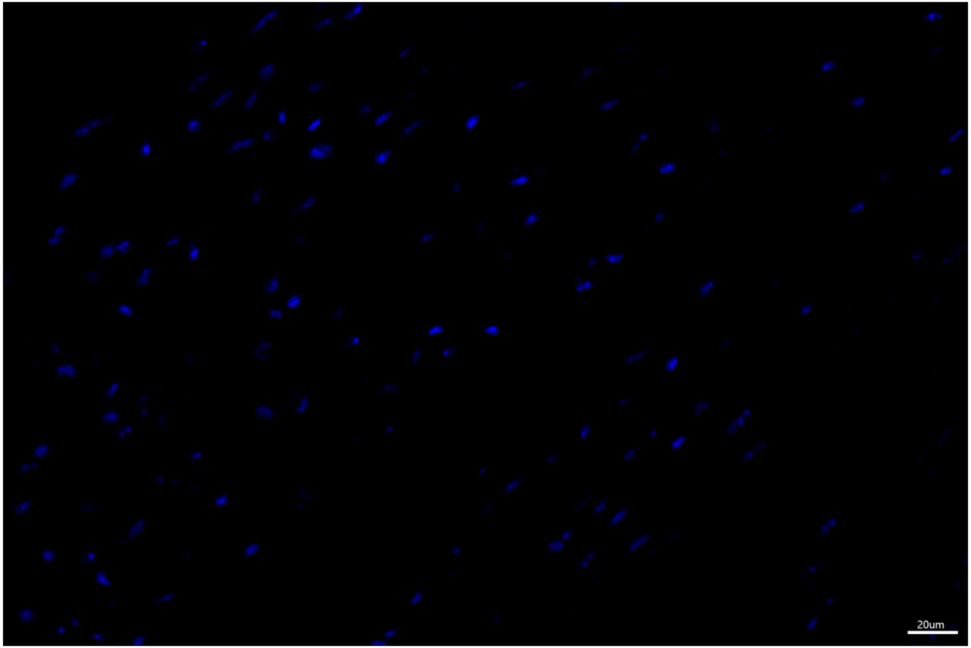

Original Image for Fig 10

(DCM+R, DAPI)

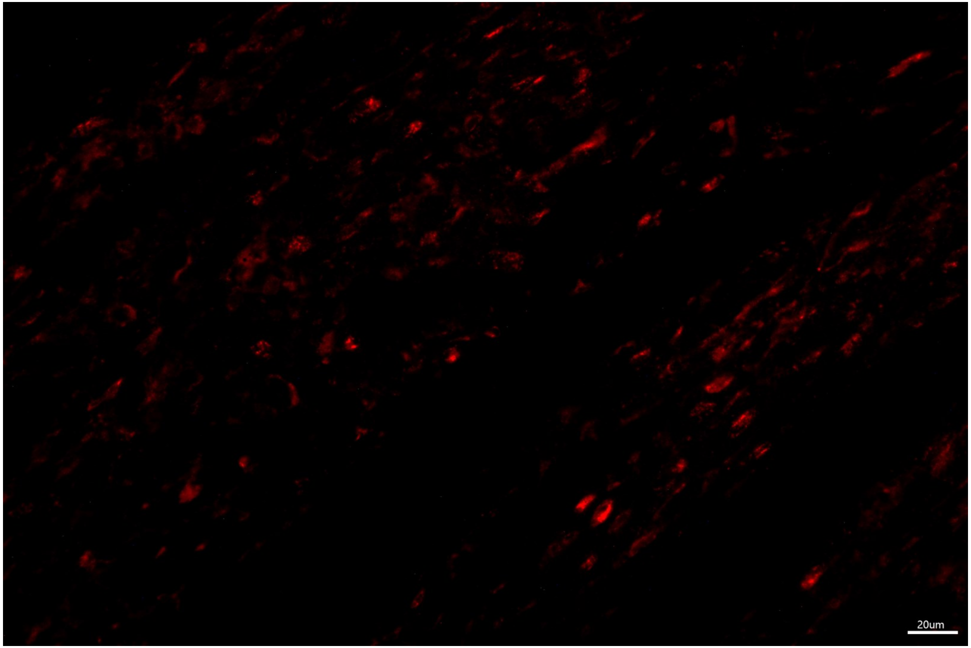

Original Image for Fig 10

(DCM+R, Collagen-I)

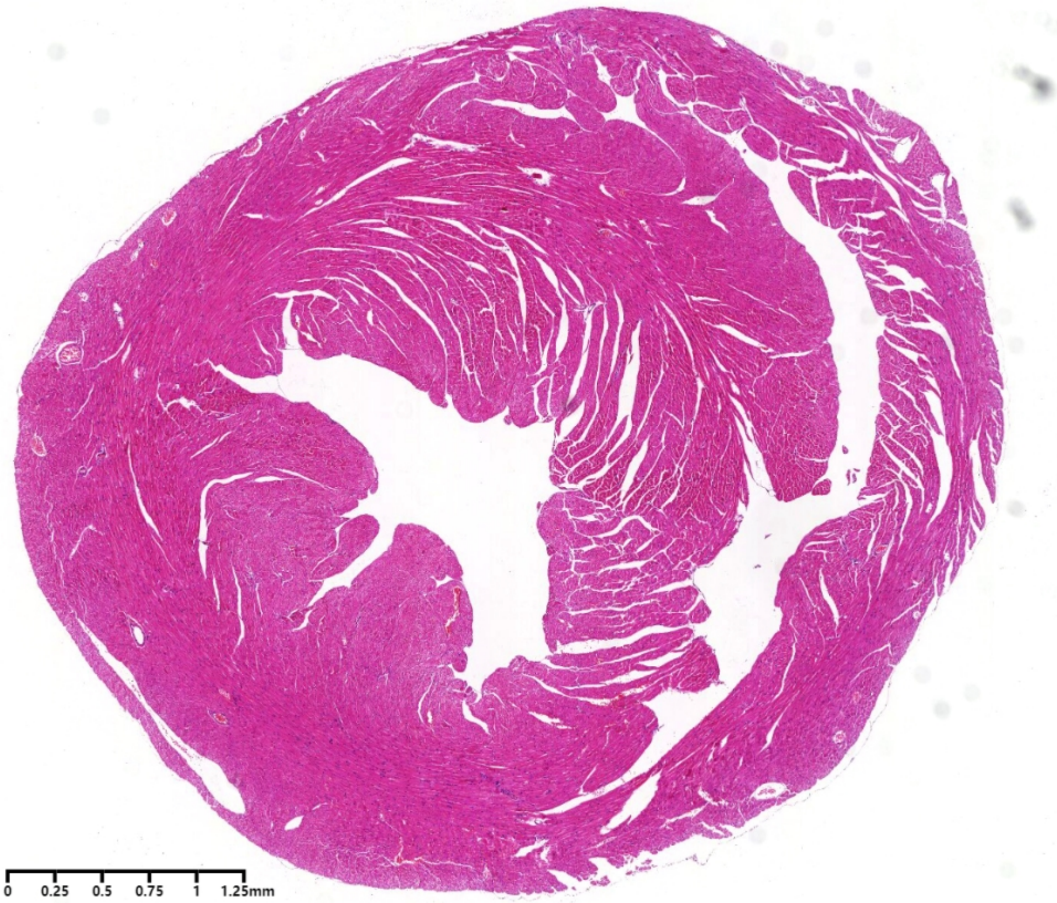

HE: Original Image for Fig 8 (Con) 2X

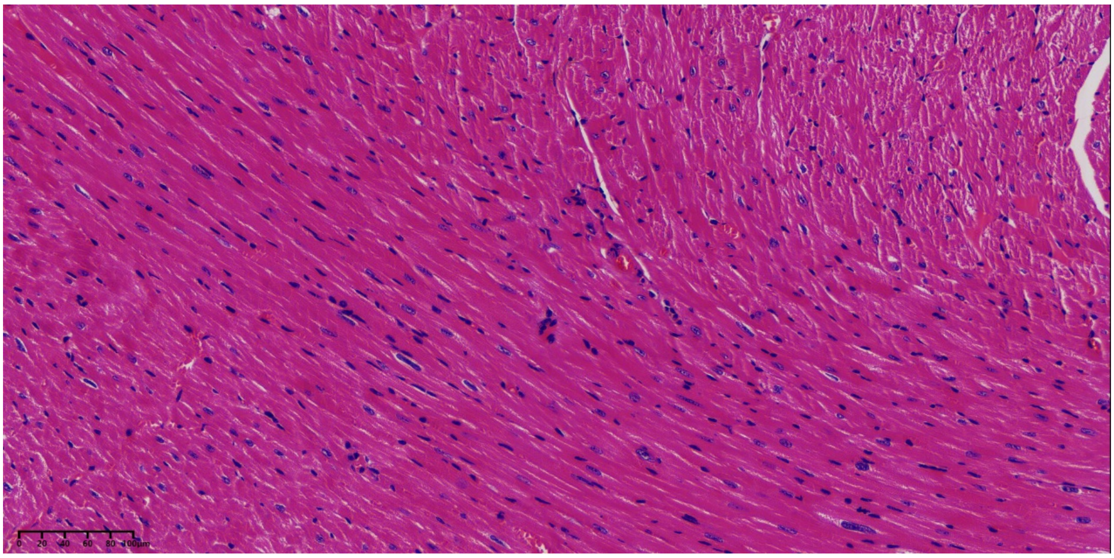

HE: Original Image for Fig 8 (Con) 20X

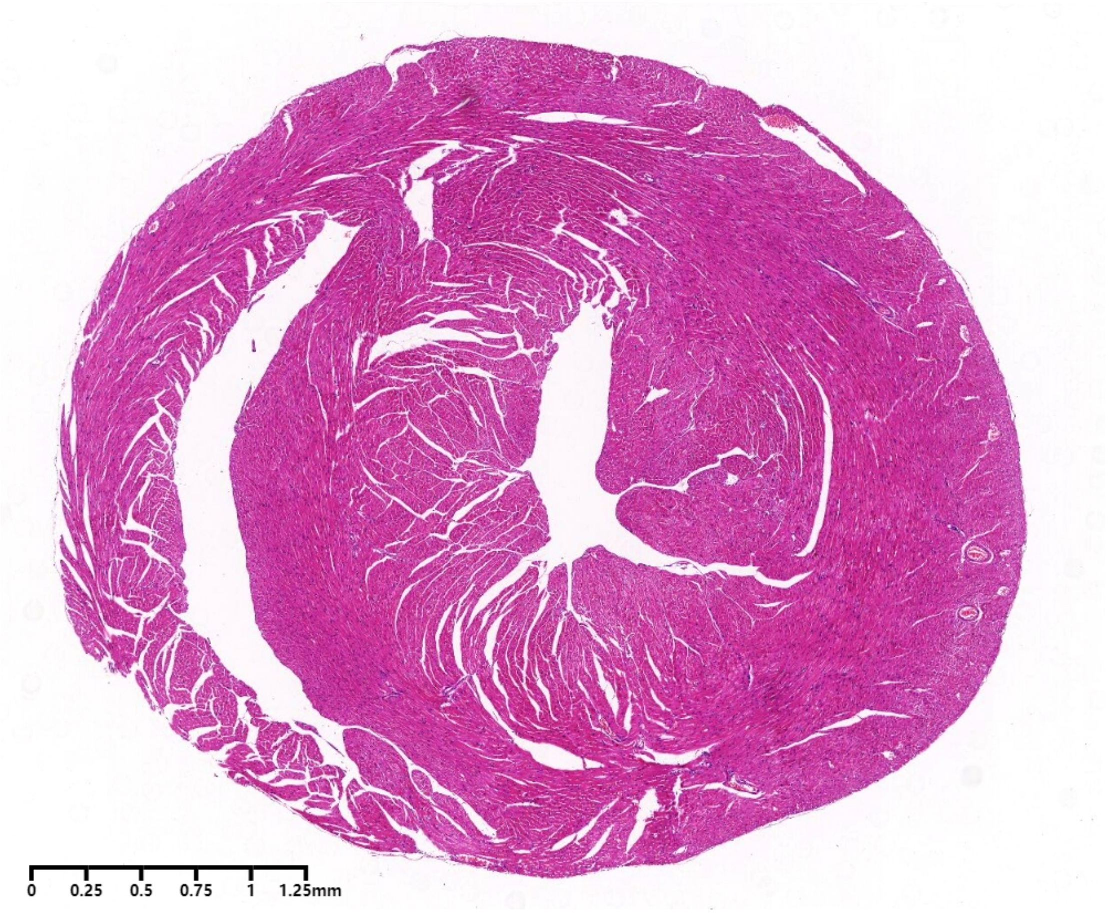

HE: Original Image for Fig 8 (DCM) 2X

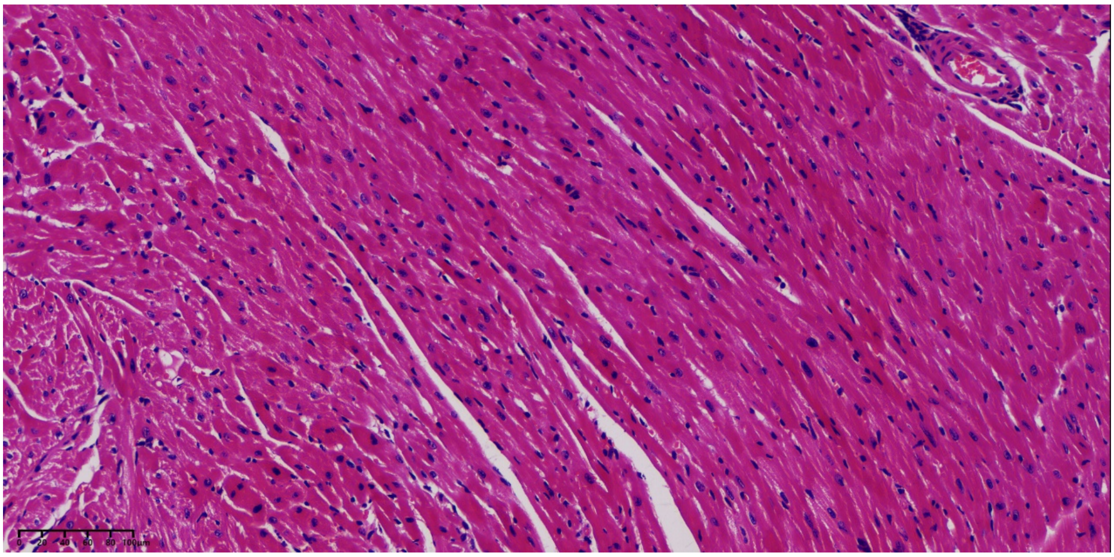

HE: Original Image for Fig 8 (DCM) 20X

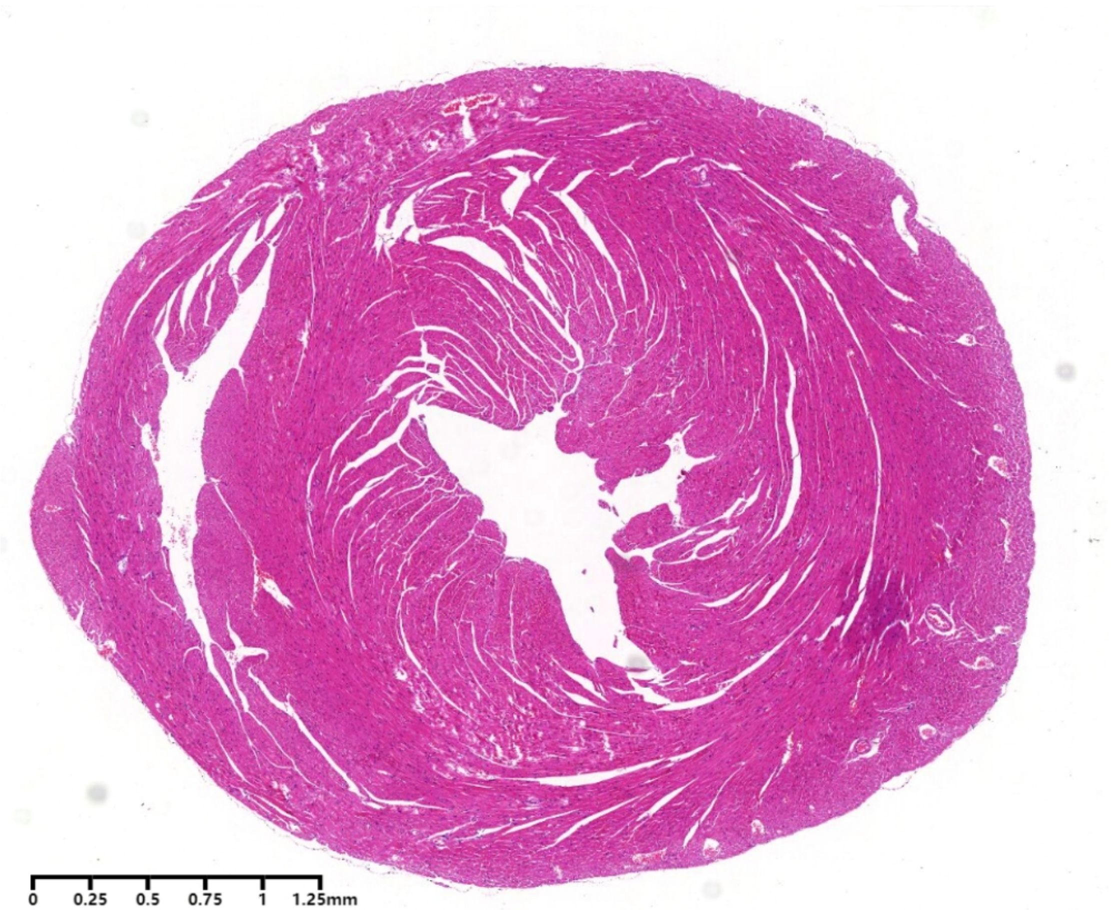

HE: Original Image for Fig 8 (DCM+ZL) 2X

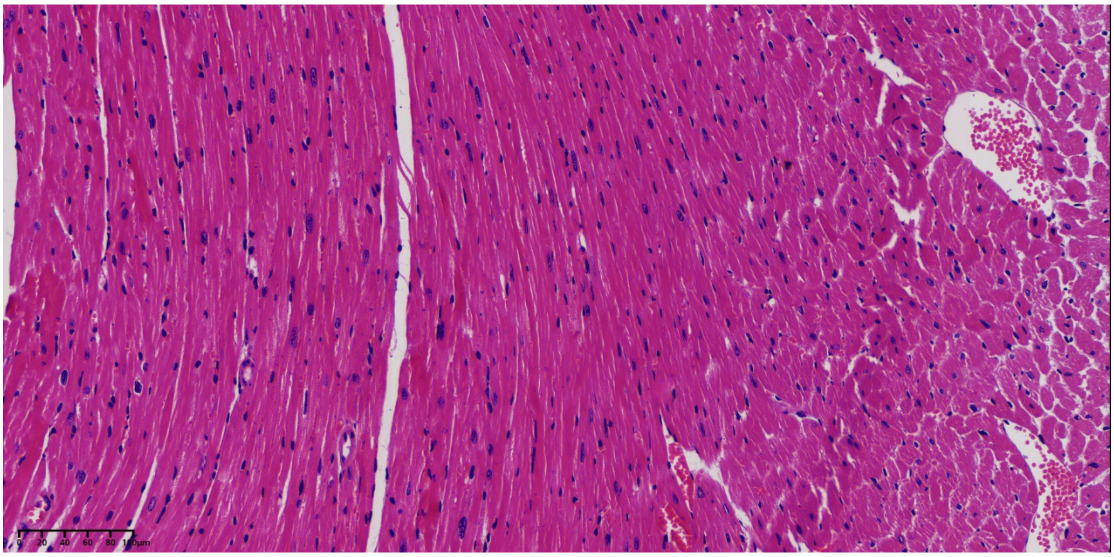
HE: Original Image for Fig 8 (DCM+ZL) 20X

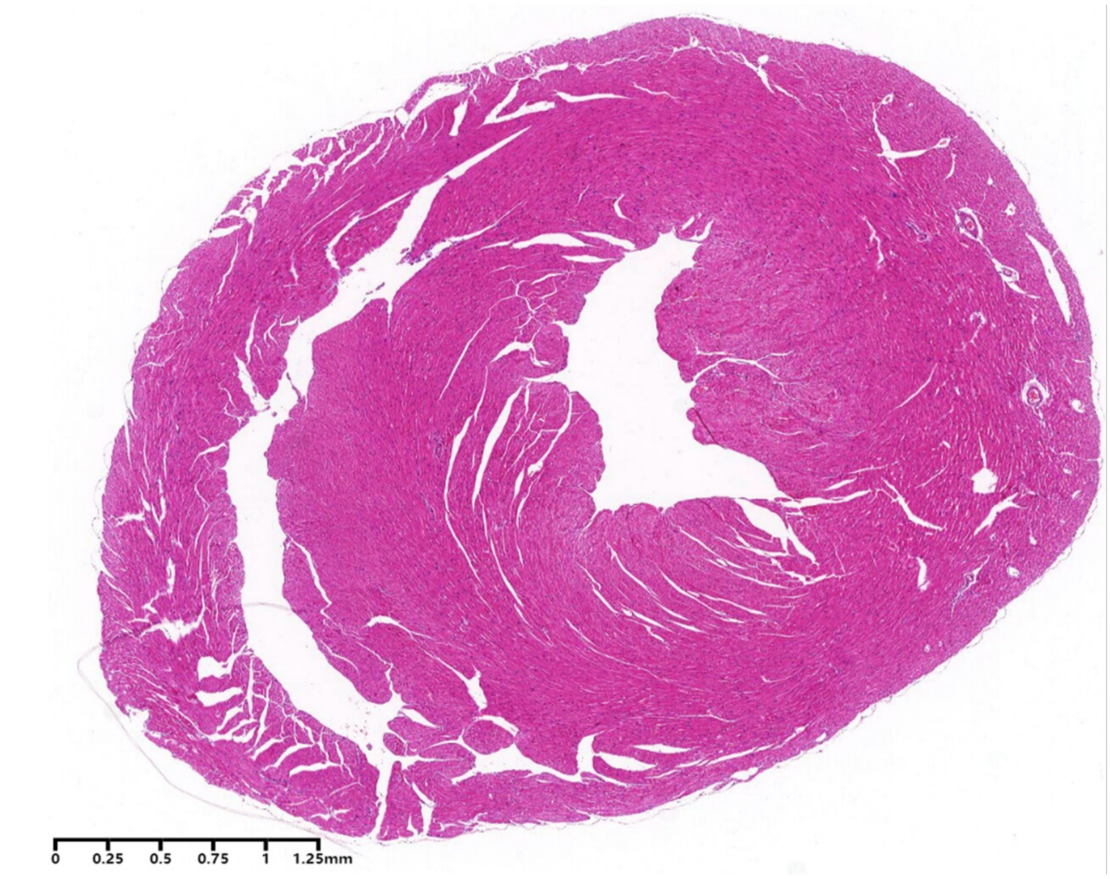

HE: Original Image for Fig 8 (SB203580) 2X

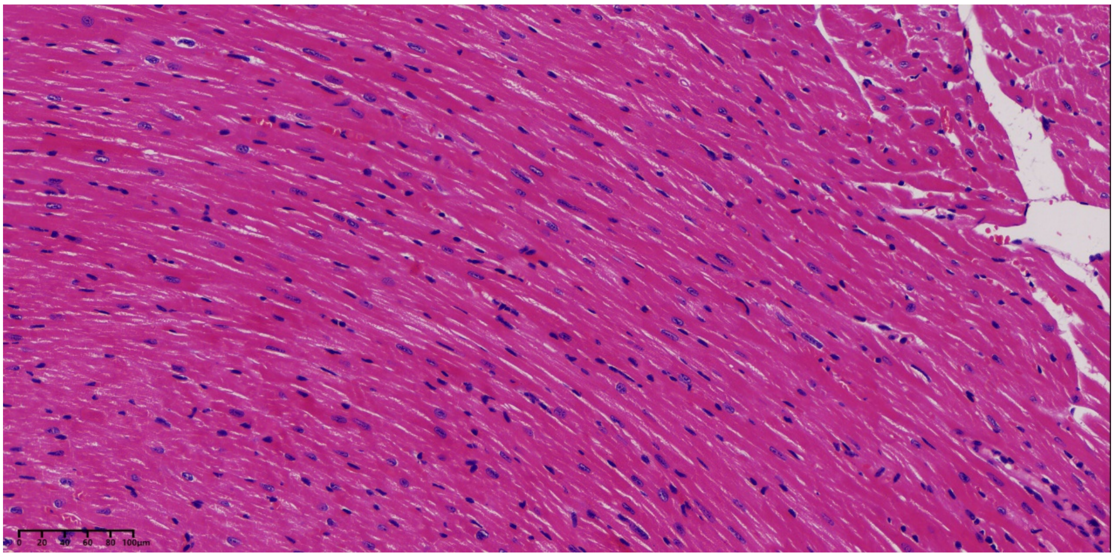
HE: Original Image for Fig 8 (SB203580) 20X

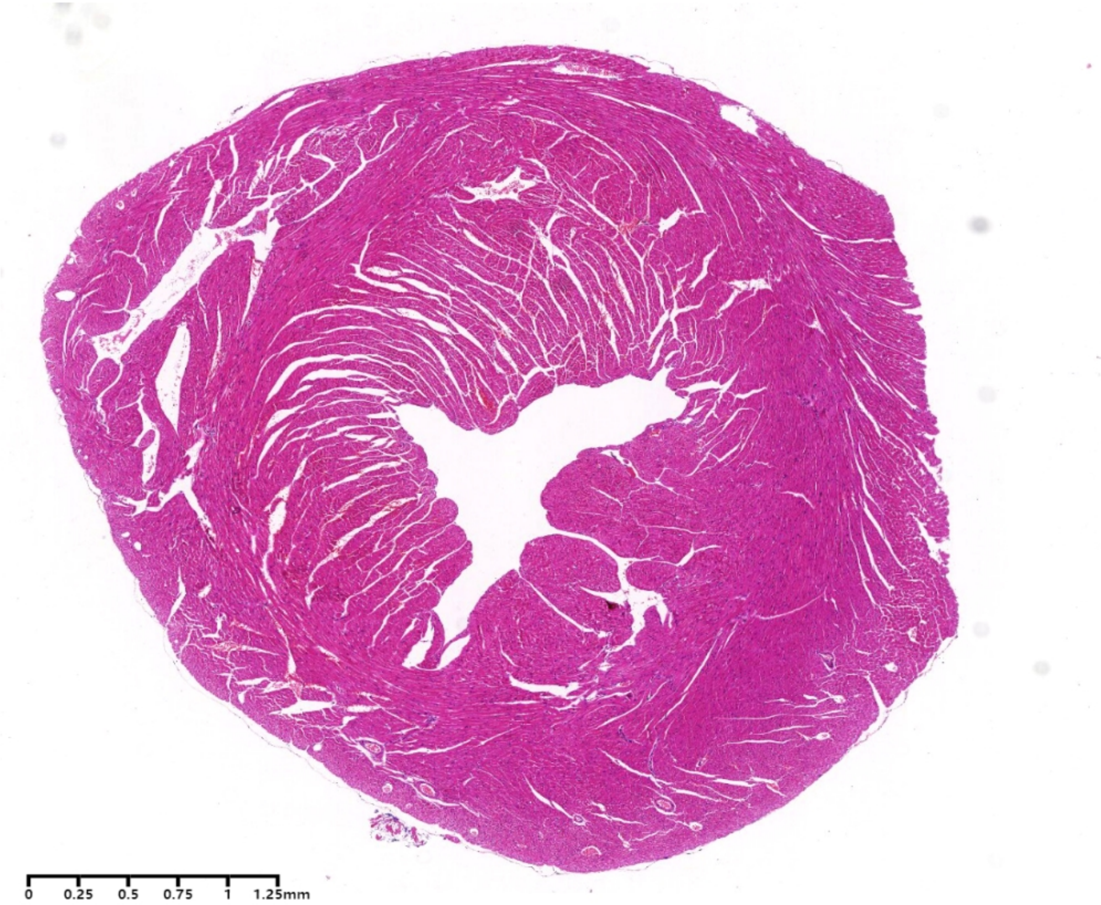

HE: Original Image for Fig 8 (DCM+R) 2X

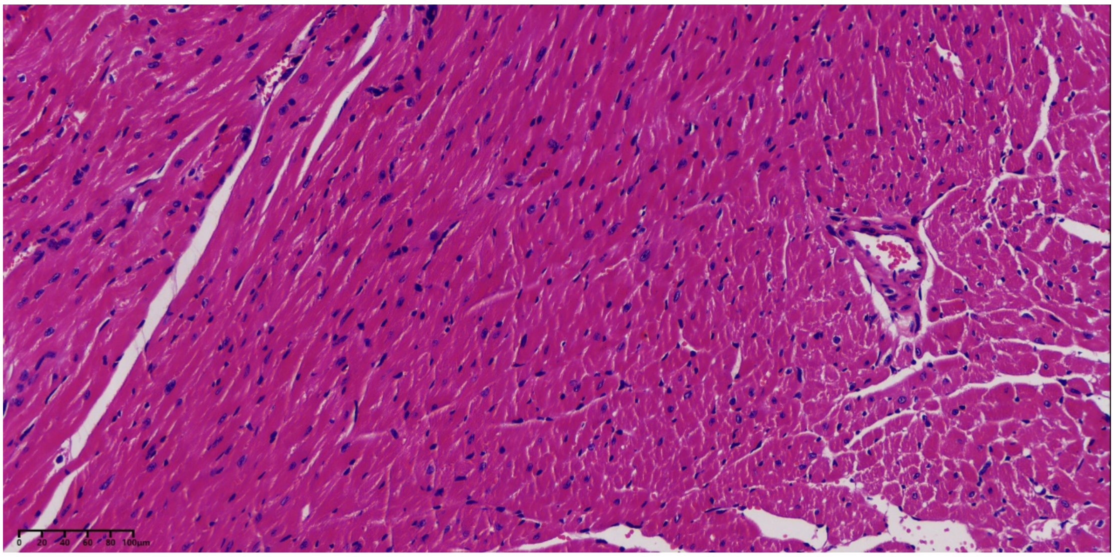

HE: Original Image for Fig 8 (DCM+R) 20X

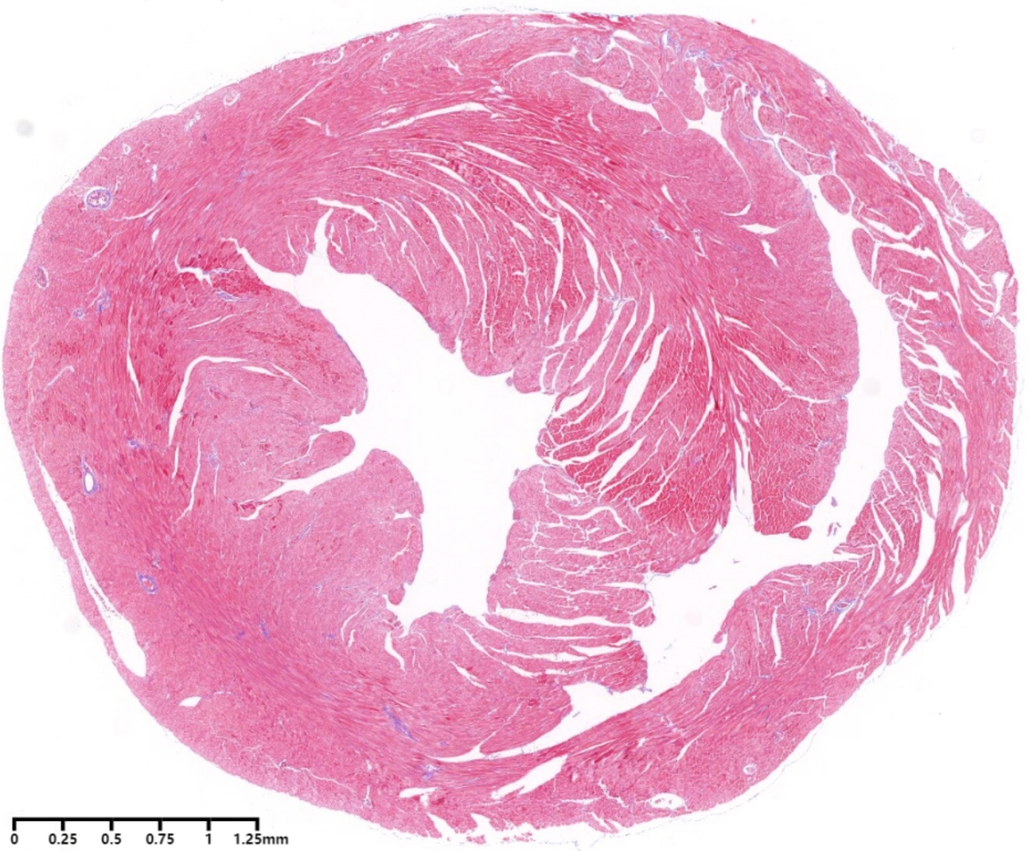

MASSON: Original Image for Fig 8 (Con) 2X

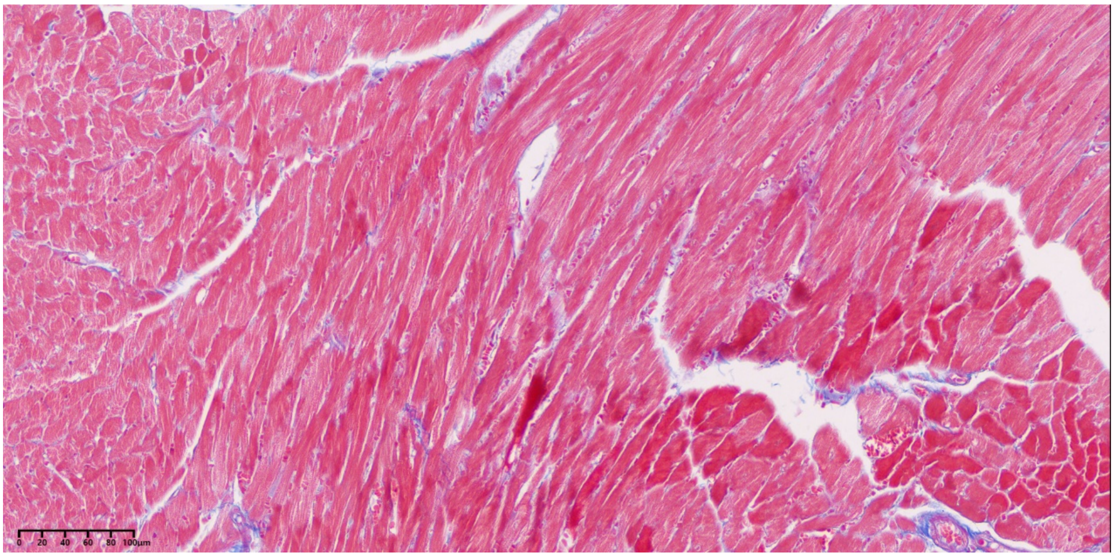

MASSON: Original Image for Fig 8 (Con) 20X

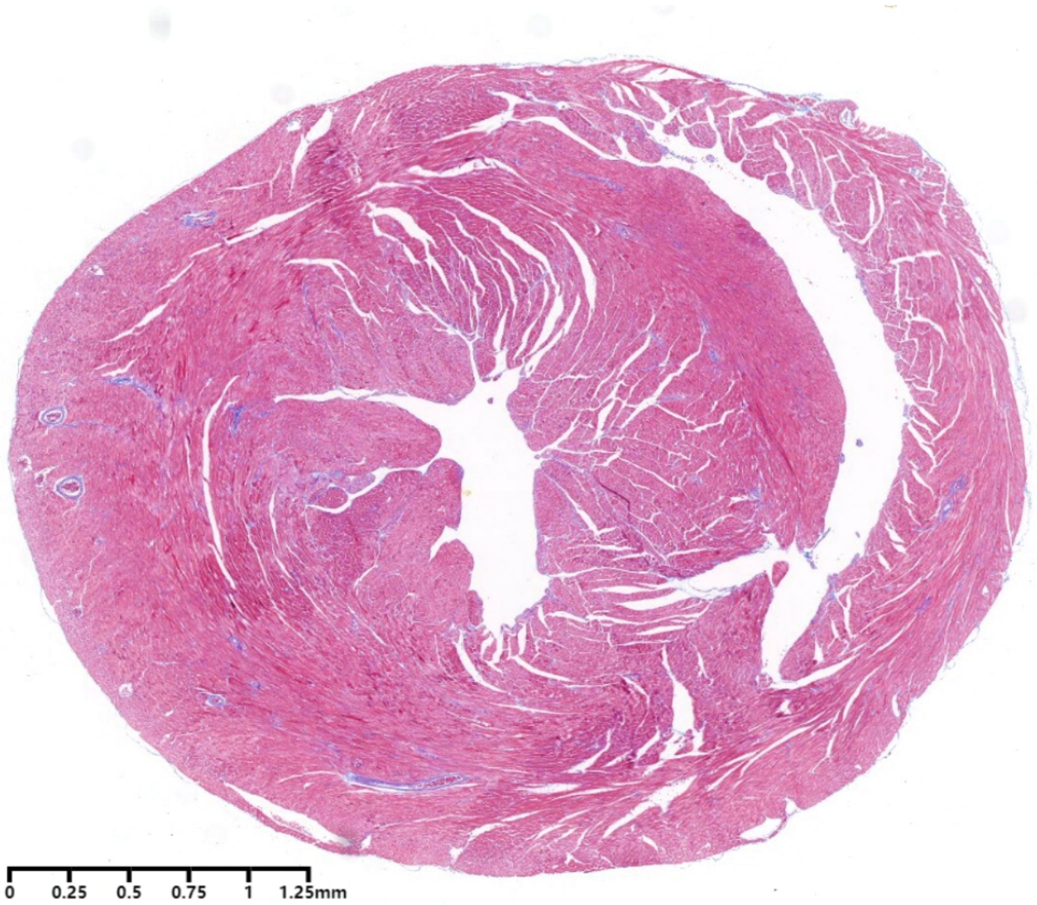

MASSON: Original Image for Fig 8 (DCM) 2X

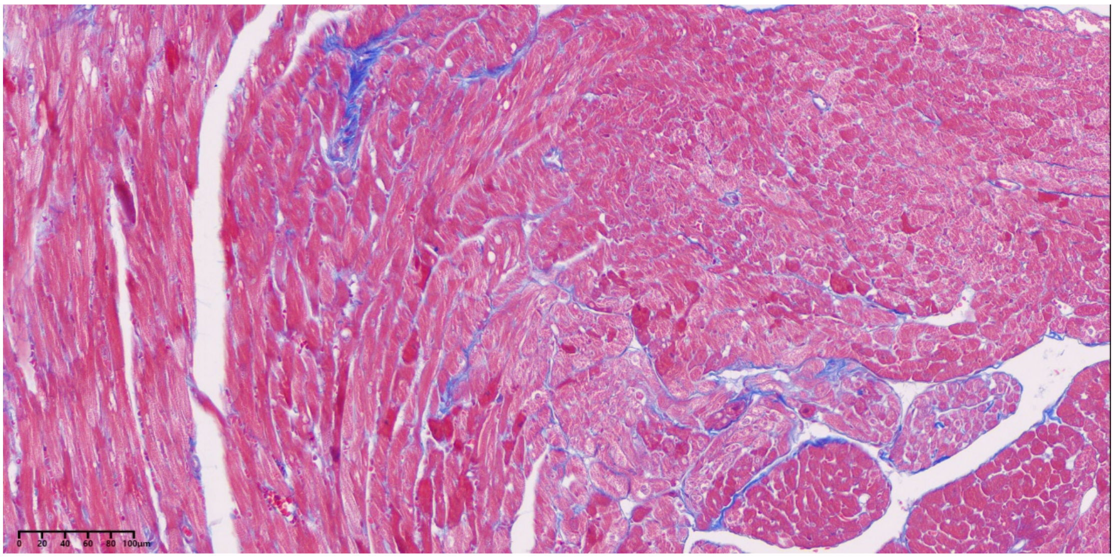

MASSON: Original Image for Fig 8 (DCM) 20X

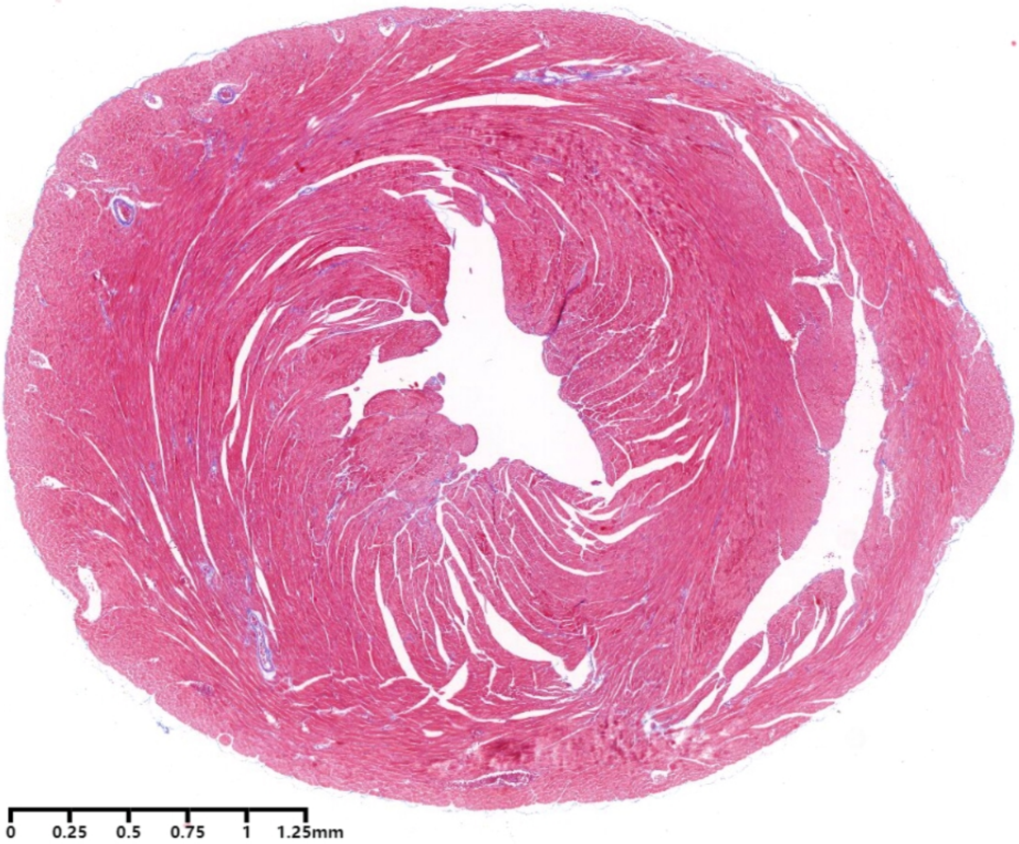

MASSON: Original Image for Fig 8 (DCM+ZL) 2X

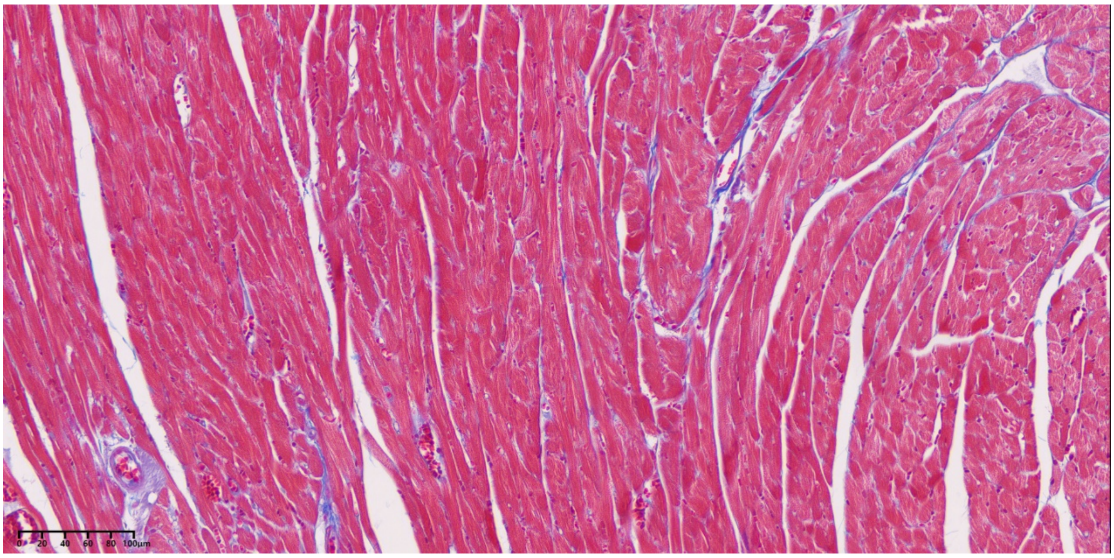

MASSON: Original Image for Fig 8 (DCM+ZL) 20X

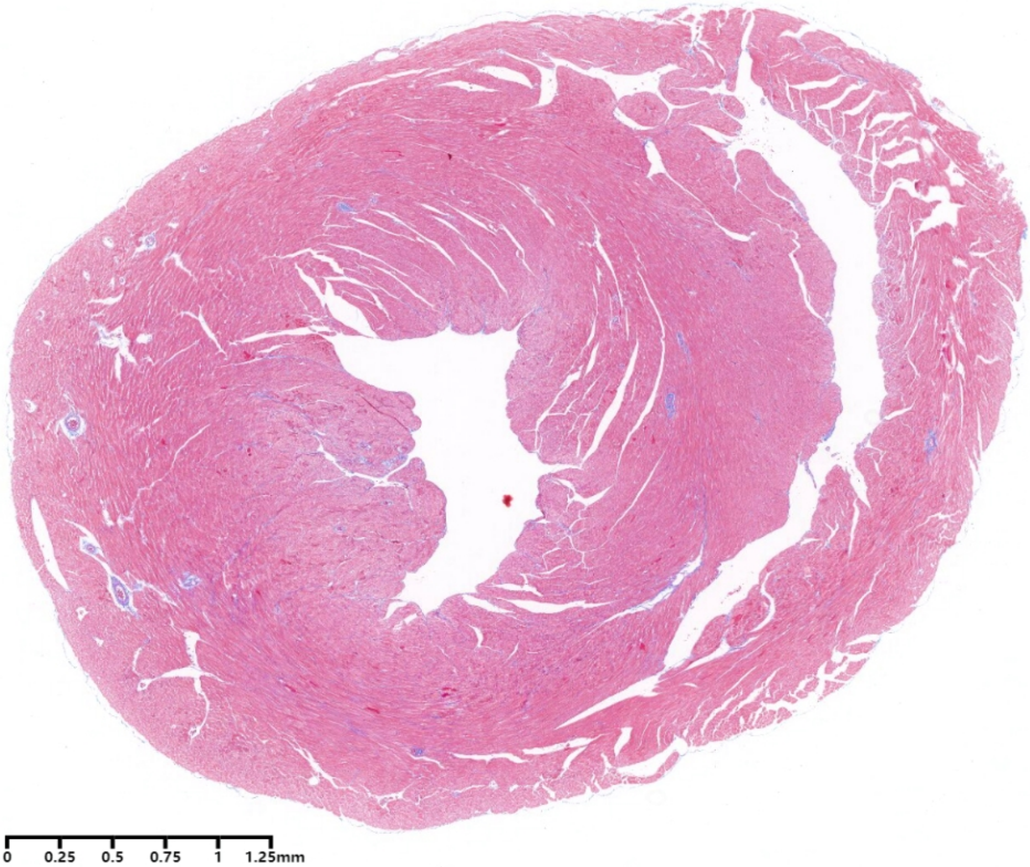

MASSON: Original Image for Fig 8 (SB203580) 2X

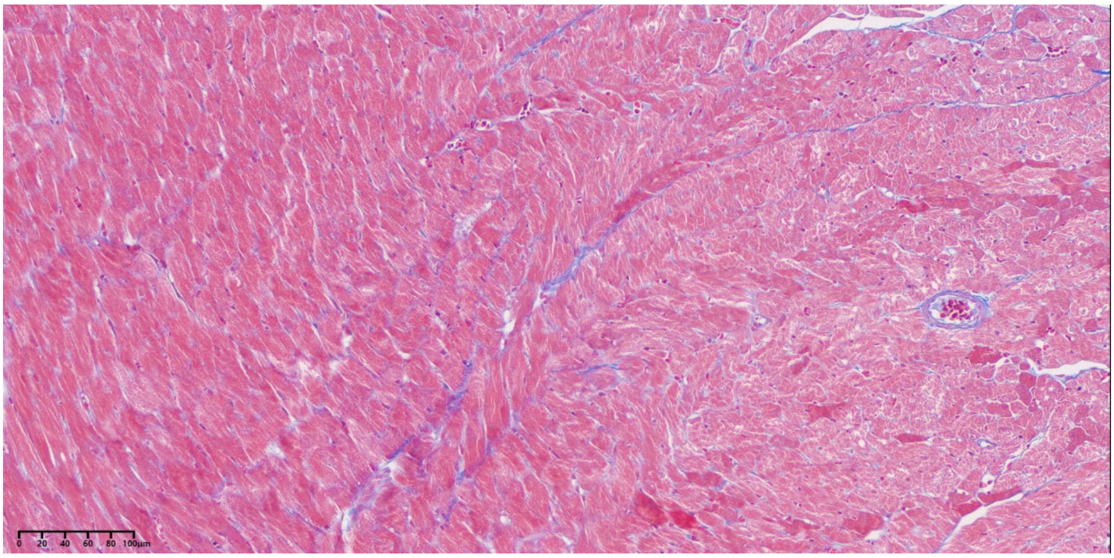

MASSON: Original Image for Fig 8 (SB203580) 20X

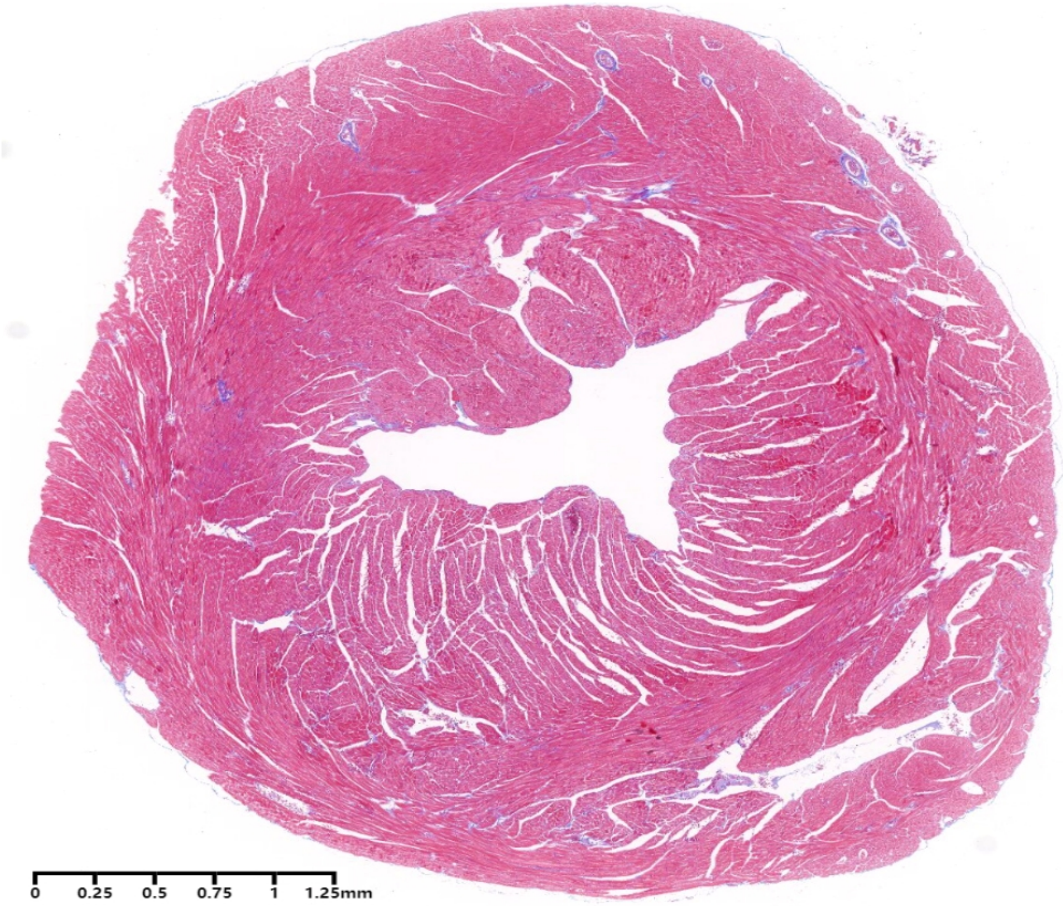

MASSON: Original Image for Fig 8 (DCM+R) 2X

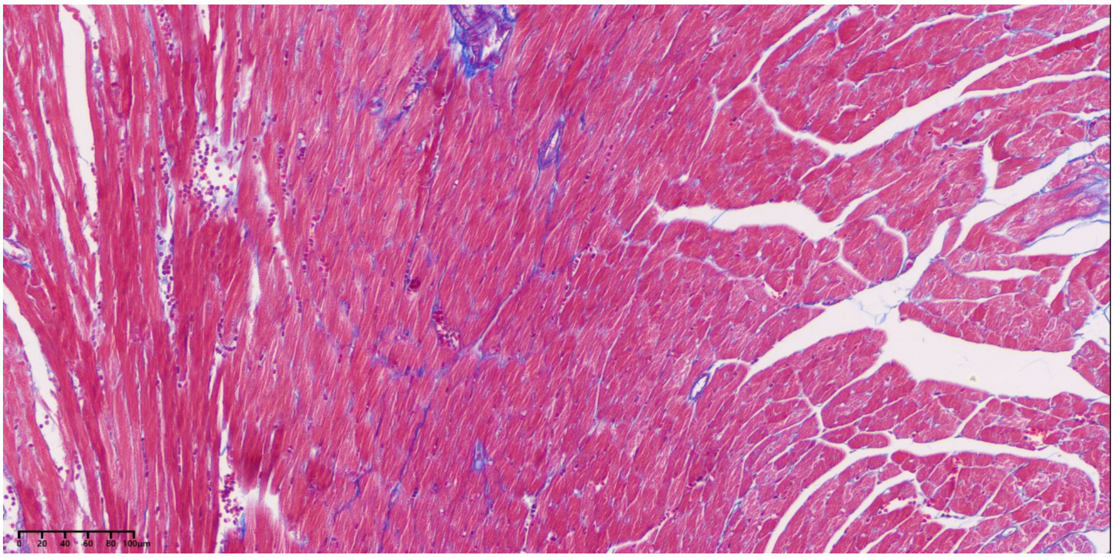
MASSON: Original Image for Fig 8 (DCM+R) 20X
