# Supplementary material for: Network pharmacology and experimental validation to investigate the mechanism of action of Zhilong Huoxue Tongyu capsule in the prevention and treatment of diabetic cardiomyopathy
Source: PLoS One. 2025 May 15;20(5):e0323745. doi: 10.1371/journal.pone.0323745 (PMC12080927; doi:10.1371/journal.pone.0323745)
Supplement: S3 — (PDF) [file pone.0323745.s003.pdf]

```
scoreFile1="score1.txt"
scoreFile2="score2.txt"
setwd("E:\\Network pharmacology\\10、cytoNCA\\filter1")
score1=read.table(scoreFile1, header=T, sep="\t",check.names =FALSE,row.names=1)
nCol=ncol(score1)
mat=data.frame()
for(i in colnames(score1)){
	print(paste0("filter1,",i,": ",median(score1[,i])))
	value=ifelse(score1[,i]>median(score1[,i]),1,0)
	mat=rbind(mat,value)
}
mat=t(mat)
colnames(mat)=colnames(score1)
row.names(mat)=row.names(score1)
geneName=row.names(mat[rowSums(mat)==nCol,])
score2=score1[geneName,]
score2out=cbind(name=row.names(score2),score2)
write.table(score2out, file=scoreFile2, sep='\t', quote=F, row.names=F)
write.table(geneName, file="score2.gene.txt", sep='\t', quote=F, row.names=F,col.names=F)


scoreFile2="score2.txt"
scoreFile3="score3.txt"
setwd("E:\\Network pharmacology\\10、cytoNCA\\filter2")
score2=read.table(scoreFile2, header=T, sep="\t",check.names =FALSE,row.names=1)
nCol=ncol(score2)
mat=data.frame()
for(i in colnames(score2)){
	print(paste0("filter2,",i,": ",median(score2[,i])))
	value=ifelse(score2[,i]>median(score2[,i]),1,0)
	mat=rbind(mat,value)
}
mat=t(mat)
colnames(mat)=colnames(score2)
row.names(mat)=row.names(score2)
geneName=row.names(mat[rowSums(mat)==nCol,])
score3=score2[geneName,]
score3out=cbind(name=row.names(score3),score3)
write.table(score3out, file=scoreFile3, sep='\t', quote=F, row.names=F)
write.table(geneName, file="score3.gene.txt", sep='\t', quote=F, row.names=F,col.names=F)
```
